# Synthesis, Antifungal Activity and Molecular Docking Studies of Novel 2-Acyloxybenzamides

**DOI:** 10.3390/molecules31132261

**Published:** 2026-06-26

**Authors:** Zilong Tang, Qianyi He, Minyi Yao, Ying Ye, Jiangtao Cai, Yichao Wan, Lifen Peng

**Affiliations:** 1 Key Laboratory of Theoretical Organic Chemistry and Functional Molecule of Ministry of Education, School of Chemistry and Chemical Engineering, Hunan University of Science and Technology, Xiangtan 411201, China22010601047@mail.hnust.edu.cn (M.Y.); 2406070328@mail.hnust.edu.cn (Y.Y.); 1060137@hnust.edu.cn (L.P.); 2 School of Life Sciences and Health, Hunan University of Science and Technology, Xiangtan 411201, China; 24010901032@mail.hnust.edu.cn

**Keywords:** 2-acyloxybenzamide, synthesis, antifungal activity, molecular docking

## Abstract

A series of 2-acyloxybenzamides were synthesized. The structures of the synthesized compounds were characterized by IR, ^1^H NMR, ^13^C NMR, and HRMS. Their antifungal activity against plant pathogenic fungi was evaluated. Compound **3i** exhibited a 100.0% inhibition rate against *M. oryzae*, while compound **3b** showed 90.0% inhibition against *P. capsici*. The corresponding EC_50_ values of compounds **3b** (against *P. capsici*) and **3i** (against *M. oryzae*) were determined to be 3.90 and 8.21 μg/mL, respectively. Molecular docking and MD simulations studies suggested that SCD enzyme may serve as a potential target for compound **3i** and its related analogues.

## 1. Introduction

Fungal pathogens pose a serious threat to global agricultural production, leading to substantial crop losses and jeopardizing food security [1,2]. The prolonged overuse of conventional fungicides has resulted in widespread resistance, diminishing their efficacy and hindering sustainable agricultural development [3,4]. Therefore, there is an urgent need to develop novel fungicides (e.g., targeting previously unexploited enzymes) with high activity, and low environmental impact [5].

Scytalone dehydratase (SCD) is widely present in various plant pathogenic fungi and serves as a key enzyme in the DHN melanin biosynthesis pathway [6]. Loss or impairment of SCD function blocks melanin synthesis, rendering pathogenic fungi non-pathogenic and unable to infect host plants. Since neither animals nor plants possess the DHN melanin biosynthesis pathway, they lack SCD and its homologous genes [7]. Consequently, SCD represents a highly selective target for fungicide development, and SCD inhibitors are considered highly safe for both animals and plants [6]. Currently available commercial SCD inhibitors mainly include carpropamid, fenoxanil, and diclocymet, all of which contain an amide bond [7]. However, resistance to existing SCD inhibitors has emerged, highlighting the critical need to develop new SCD inhibitors.

Amide compounds, due to their unique structure, exhibit a wide range of biological activity, including antifungal [5], antiviral [8,9], antibacterial [10,11,12], anti-inflammatory [13], antitumor [14,15,16], anticancer [17,18], antiprotozoal [19], as well as insecticidal and acaricidal [20,21] activity. Among them, salicylamide derivatives have demonstrated significant antibacterial and antifungal activity. For instance, Kubanová et al. [22] reported that *N*-benzylsalicylamide derivatives exhibited potent antifungal activity against Trichophyton mentagrophytes with MIC values ≤ 7.8 μmol/L. Ma et al. [23] demonstrated that oxyclozanide, a salicylamide anthelmintic, showed strong antibacterial activity against phytopathogenic bacteria such as Xanthomonas oryzae (MIC = 0.78 μg/mL) and could induce plant defense responses. These findings underscore the use of the salicylamide scaffold in antimicrobial agent development.

Our research group has long been dedicated to the design and synthesis of amide compounds with potent antifungal activity [24,25]. We observed that 2-((2-hydroxyphenyl)methylamino)acetamides possess excellent antifungal activity [25].

**Scheme 1:**
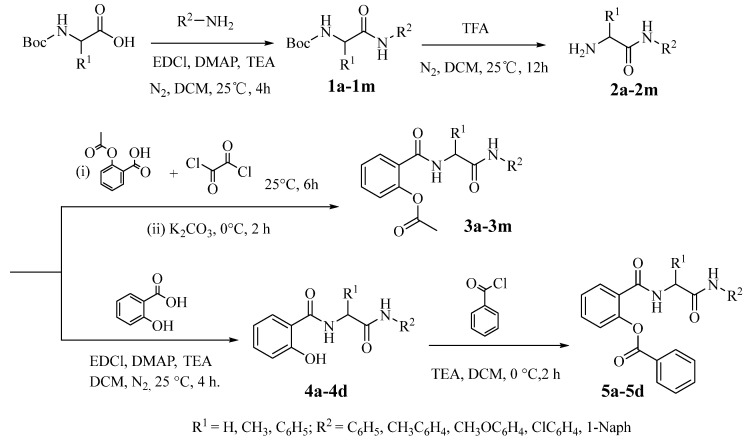
Synthesis of 2-acyloxybenzamide compounds **3** and **5**.

Based on the above analysis, a series of novel 2-acyloxybenzamide compounds **3** and **5** (Scheme 1) were designed to target SCD. The synthesis, antifungal activity, and molecular docking studies of the active target compounds with SCD will be reported in detail.

## 2. Results and Discussion

### 2.1. Synthesis

The synthetic route to 2-acyloxybenzamide compounds is outlined in Scheme 1. Initially, Boc-protected amino acids were condensed with various amines to afford *N*-Boc-aminoacyl amides **1**. Subsequent Boc deprotection yielded the corresponding aminoamides **2** (see Supplementary Materials). These key intermediates were then elaborated into the final target compounds **3** and **5** via two distinct pathways.

#### 2.1.1. Synthesis of 2-Acetoxybenzamide Derivatives **3**

Acetylsalicylic acid was converted to acetylsalicyloyl chloride by treatment with oxalyl chloride in dry CH_2_Cl_2_ at room temperature. The resulting acyl chloride was then added dropwise to a mixture of aminoacyl amine **2** and K_2_CO_3_ in an ice-water bath and stirred for 2 h to afford the desired products **3a**–**3m** in 46–62% yield (Table 1).

For intermediates **1**, the influence of R^2^ on yields followed the order CH_3_OC_6_H_4_ ≈ CH_3_C_6_H_4_ > C_6_H_5_ > ClC_6_H_4_ > 1-Naph (Table 1). This trend can be rationalized by the electronic effects of the substituents on the benzene ring. Since the reaction involves a nucleophilic addition process, electron-donating groups increase the electron density on the amino nitrogen atom, thereby enhancing its nucleophilicity and promoting the reaction. Conversely, electron-withdrawing groups decrease the electron density on the nitrogen atom, reducing its nucleophilicity and thus diminishing the reaction activity.

As for target compounds **3**, when the substituent on the benzene ring of R^2^ remained the same, the reactivity followed the order: *para* > *meta* > *ortho*. This order is primarily governed by steric hindrance, even though the R^2^ group is relatively distant from the reaction center. The *ortho* substituent is located in close proximity to the amino group, creating significant steric crowding during the acylation reaction and resulting in the lowest yield. The *meta* substituent imposes moderate hindrance, whereas the *para* substituent, being farthest from the reaction site, experiences the least steric interference and thus gives the highest yield among the three isomers. In addition, the 1-naphthyl group, which is bulkier than the phenyl ring, introduces the greatest steric hindrance, consistent with the lowest yield observed for this analogue. In contrast, although the R^1^ group is closer to the reaction center, its size had only a minor influence on the reaction yield; substituents R^1^ such as H, methyl, or phenyl groups exhibited no significant differences in yield.

#### 2.1.2. Synthesis of 2-Benzoyloxybenzamide Derivatives **5**

The intermediates salicylamide **4** were synthesized in 51–59% yield by condensation reaction of salicylic acid with aminoacyl amines **2**, using EDCI as the condensation reagent, DMAP and Et_3_N as catalyst and base (Table 2) (see Supplementary Materials). Subsequently, these intermediates **4** were subjected to react with benzoyl chloride in the presence of Et_3_N at 0 °C, furnishing the target compounds **5a**–**5d** in 38–74% yield finally (Table 2).

For intermediates **4** and the target compounds **5**, the effects of the substituent on the benzene ring of R^2^ followed a similar trend: compounds bearing a substituent on the benzene ring afforded higher yields than those without a substituent. Moreover, the position of substituent on the benzene ring exerted the same effect on both intermediates **4** and target compounds **5**, following the order: *para* > *meta*. This order is primarily governed by steric hindrance.

### 2.2. Antifungal Activity Assay

According to the standardized method NY/T1156.5-2006 [25], the inhibitory activity of 2-acyloxybenzamide derivatives **3** and **5** were evaluated at a concentration of 50 μg/mL (Table 3). As shown in Table 3, the compounds exhibited moderate to excellent activity against all tested pathogens, with the strongest activity observed against *M. oryzae* nd *P. capsici*. For instance, compound **3i** displayed 100.0% inhibition rate against *M. oryzae*, while compounds **3d** and **3j** showed inhibition rates of 80.6% and 86.5%, respectively—all higher than that of the positive control chlorothalonil (53.8%). Against *P. capsici*, compound **3b** exhibited a 90.0% inhibition rate, surpassing that of chlorothalonil (89.3%); compounds **3j** and **3m** also showed 83.3% and 80.0% inhibitory activity, respectively. Additionally, compound **3m** displayed 73.2% activity against *R. solani*, and compound **5a** exhibited 72.5% activity against *S. sclerotiorum*.

A certain structure–activity relationship was observed: compounds with R^1^ as a hydrogen exhibited lower activity than those R^1^ as methyl or aryl groups (except for *B. cinerea*). For R^2^, compounds bearing a substituent on the benzene ring generally show higher activity than those without a substituent (except for *B. cinerea*). No clear structure–activity relationships were observed for the remaining compounds. For example, compound **3i** (R^1^ = methyl) showed 100% inhibition activity against *M. oryzae*, which higher than the activity of compound **5b** (R^1^ = phenyl); however, compound **3h** (R^1^ = methyl) displayed lower inhibition activity against *S. sclerotinia* than compound **5a** (R^1^ = phenyl).

Table 4 presents the results of the second screening for compounds **3b** and **3i**, which exhibited excellent activity against *P. capsica* and *M. oryzae*, respectively. The EC_50_ value of compound **3b** against *P. capsica* is close to those of the reference fungicide chlorothalonil. Notably, the EC_50_ of compound **3i** against *M. oryzae* is 8.21 µg/mL, which is substantially lower than that of chlorothalonil (33.56 µg/mL). These results suggest that compounds **3b** and **3i** hold promise as potential fungicidal candidates or lead compounds.

### 2.3. Molecular Docking and Molecular Dynamics (MD) Simulation

Molecular docking studies of inhibitors with their target enzymes or proteins in pathogenic fungi can greatly assist in evaluating the potential of inhibitors to suppress enzyme activity. In silico analyses, including molecular docking and protein–ligand interaction studies of antifungal metabolites with their target enzymes, are also important for understanding the mechanism of antifungal action and assessing their potential as novel fungicide candidates. Recently, Khan et al. employed molecular docking and molecular dynamics analysis to identify potent natural fungicides targeting the SCD of *M. oryzae* [26].

**Figure 1:**
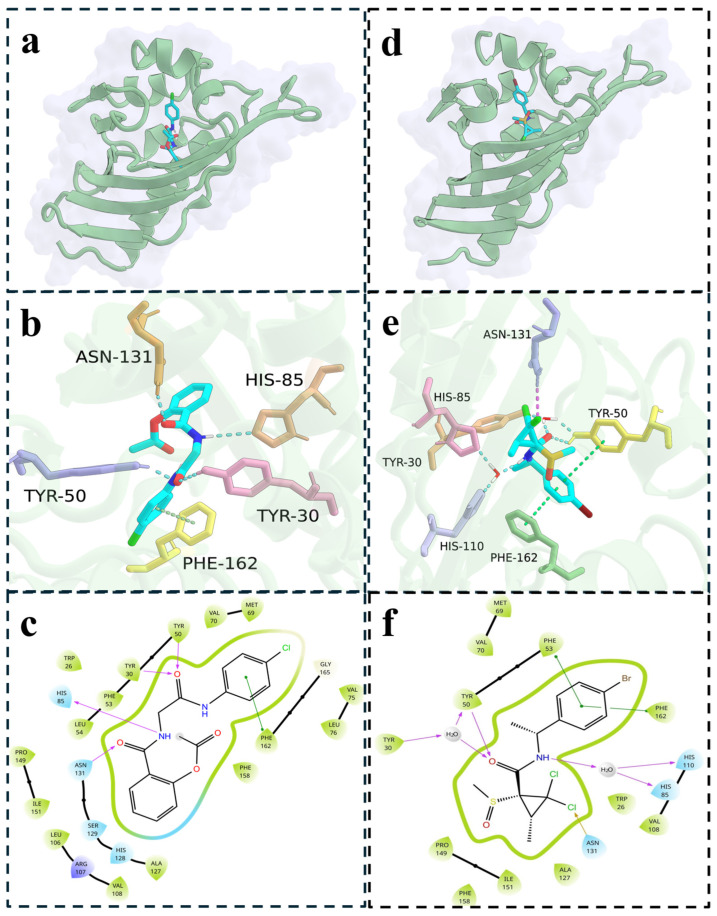
Co–crystal structure of SCD with inhibitor MS2 (**a**–**c**) and molecular docking of compound **3i** (**d**–**f**) with the target enzyme SCD (PDB ID: 6STD). (**a**,**d**) Interactions represented as a combined protein surface transparency model and cartoon model. (**b**,**e**) 3D representations of the interactions with surrounding amino acid residues in the ligand–binding site of SCD. (**c**,**f**) 2D schematic illustrations of these interactions.

In this study, we investigated the potential interactions between our newly synthesized compounds and the SCD enzyme. Compound **3i**, which exhibited excellent antifungal activity, was selected as a representative derivative for molecular docking studies targeting the active site of SCD (PDB ID: 6STD) using the AutoDock Vina 1.1.2 program. The co-crystal structure of SCD with inhibitor MS2 (2,2-dichloro-1-methanesulfinyl-3-methyl-cyclopropanecarboxylic acid (1-(4-bromophenyl)ethyl)amide) is shown in Figure 1a,c [27].

Docking results revealed that compound **3i** adopted conformations and binding orientations similar to those of MS2 within the active site of SCD, with a docking score of −8.50 kcal/mol. In the SCD-MS2 complex, MS2 formed direct hydrogen bonds with residues Tyr50 and Asn131, as well as water-mediated hydrogen bonds with His85, His110, and Tyr30 (Figure 1b,c). Additionally, π-stacking interactions were observed with Phe53 and Phe162. In comparison, compound **3i** formed direct hydrogen bonds with Asn131, His85, Tyr50, and Tyr30 (Figure 1e,f), along with a π-stacking interaction with Phe162. These findings demonstrate that compound **3i** exhibits a favorable binding mode and strong interaction with the active site of SCD, particularly highlighting the critical role of the amide bond.

**Figure 2:**
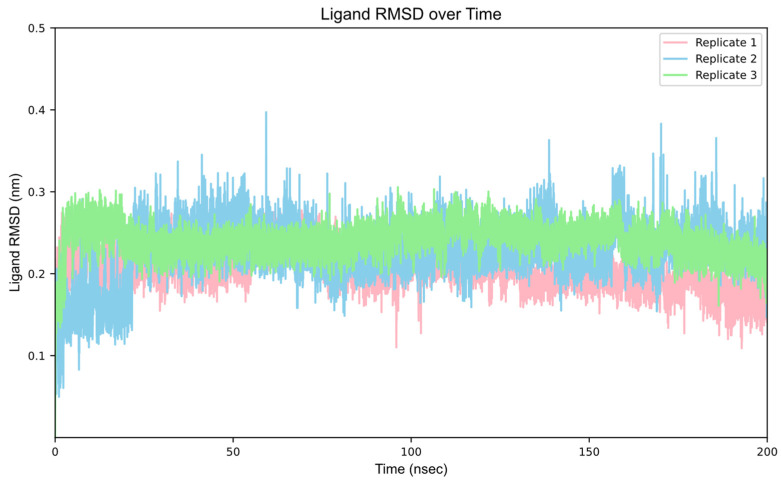
Molecular dynamics simulation of SCD-**3i** complex.

Moreover, to elucidate the stability of the inhibitor–protein interaction, MD simulations of the inhibitor–protein complex were conducted using GROMACS 2023.4 program. The root mean square deviation (RMSD) derived from the MD simulation was employed to examine the stability of the interaction between compound **3i** and SCD. Greater protein stability is indicated by smaller RMSD fluctuations. It was observed that the SCD-**3i** complex maintained a constant RMSD value of approximately 0.24 nm from the beginning to approximately 200 ns (Figure 2). These results indicate strong conformational stability of the SCD-**3i** complex. Additionally, the binding free energies of the SCD–compound **3i** and SCD-MS2 complexes were calculated using the MM-GBSA method, yielding values of −77.79 kcal/mol and −68.54 kcal/mol, respectively.

Taken together, the above results strongly support the hypothesis that SCD may serve as a potential target for compound **3i** and the related 2-acyloxybenzamide derivatives, and provide valuable insights for rational design of future antifungal agents targeting SCD.

## 3. Materials and Methods

### 3.1. Materials and Reagents

^1^H and ^13^C NMR spectra were recorded on a Bruker AVANCE NEO 400 MHz spectrometer (Bruker, Fällanden, Switzerland; 400 MHz for ^1^H and 100 MHz for ^13^C), with tetramethylsilane (TMS) as the internal standard. The chemical shifts (*δ*) and coupling constants (*J*) are given in ppm and Hertz (Hz), respectively. Infrared spectra (IR) spectra were recorded on a Thermo Scientific Nicolet-6700 FT-IR spectrometer (Thermo Fisher Scientific Inc., Dover, DE, USA). High-resolution mass spectra (HRMS) were recorded on Waters ACQUITY UPLC H-Class/Waters Xevo G2-S QTof instrument (Waters, Milford, MA, USA). Melting points were determined on a WRS-2 digital melting point apparatus (Shanghai Instrument Physical Optics Instrument Co., Ltd., Shanghai, China) and were uncorrected. Column chromatography was performed using silica gel with a particle size of 200–300 mesh. Reagents were purchased from MERYER or Energy Chemical Company (Shanghai, China) as analytical grade and used without further purification. All anhydrous solvents were dried by standard procedures.

### 3.2. Chemical Synthesis

#### 3.2.1. Synthesis of 2-Acetoxybenzamide Derivatives **3a**–**3m**

*General Procedure:* In a 50 mL round-bottom flask, acetylsalicylic acid (0.181 g, 1.0 mmol) and 10 mL of dry dichloromethane were added. Under nitrogen atmosphere, oxalyl chloride (0.254 g, 2.0 mmol) was added dropwise at room temperature while stirring. After reacting for 0.5 h, a drop of *N*,*N*-dimethylformamide (DMF) was added, and the reaction was continued for 6 h to obtain 2-acetylsalicyloyl chloride. In a 100 mL round-bottom flask, *N*-phenyl-2-aminoacetamide (**2a**) (0.300 g, 2.0 mmol), K_2_CO_3_ (0.166 g, 1.2 mmol), and 10 mL of dry dichloromethane were added, and the mixture was placed in an ice-water bath. 2-Acetylsalicyloyl chloride was added dropwise while stirring, and the reaction was continued for 2 h. After the reaction was complete (monitored by TLC), the solvent was removed under reduced pressure, 30 mL of distilled water was added, and the mixture was extracted with dichloromethane (30 mL × 3). The combined organic phases were dried over anhydrous Na_2_SO_4_, filtered, and the solvent was removed under reduced pressure. The remaining residue was purified by column chromatography (eluent: petroleum ether/ethyl acetate = 3:1, R_f_ = 0.25) to obtain product **3a** as a white solid with a yield of 48%.

*N-(Phenylcarbamoylmethyl)-2-acetoxybenzamide* (**3a**): Yield 48%, white solid, mp 172.2–174.2 °C. ^1^H NMR (400 MHz, DMSO-*d*_6_) *δ*: 10.04 (s, 1H), 8.51 (t, *J* = 5.7 Hz, 1H), 7.72 (d, *J* = 6.1 Hz, 1H), 7.61 (d, *J* = 8.0 Hz, 2H), 7.55 (t, *J* = 6.8 Hz, 1H), 7.39-7.30 (m, 3H), 7.21 (d, *J* = 8.0 Hz, 1H), 7.06 (t, *J* = 7.4 Hz, 1H), 4.05 (d, *J* = 5.7 Hz, 2H), 2.27 (s, 3H). ^13^C NMR (100 MHz, DMSO-*d*_6_) *δ:* 169.15, 167.50, 165.34, 148.13, 139.93, 131.69, 129.46, 128.86(2C), 128.46, 125.96, 123.55, 123.33, 119.12 (2C), 43.14, 20.91. IR (KBr, cm^−1^): 3398, 3283, 3145, 2997, 2842, 1773,1701, 1640, 1602, 1558, 1523, 1480, 1445, 1369, 1286, 1256, 1176, 1047, 910, 757. HRMS(ESI) calcd for C_17_H_16_N_2_O_4_Na (M + Na)^+^: 335.1008, found: 335.1010.

*N-((2-methylphenylamino)carbonylmethyl)-2-acetoxybenzamide* (**3b**): Yield 46%, white solid, mp 157.1–157.8 °C. ^1^H NMR (400 MHz, DMSO-*d*_6_) *δ*: 9.35 (s, 1H), 8.55 (t, *J* = 5.8 Hz, 1H), 7.76-7.73 (m, 1H), 7.55-7.53 (m, 1H), 7.46 (t, *J* = 8.1 Hz, 1H), 7.39-7.35 (m, 1H), 7.23-7.18 (m, 3H), 7.09 (t, *J* = 7.4 Hz, 1H), 4.09 (d, *J* = 5.7 Hz, 2H), 2.28 (s, 3H), 2.27 (s, 3H). ^13^C NMR (100 MHz, DMSO-*d*_6_) *δ*: 169.07, 167.55, 165.35, 138.14, 136.05, 131.67, 131.58, 130.35, 129.46, 128.35, 126.02, 125.91, 125.22, 124.72, 123.50, 43.04, 20.85, 17.80. IR (KBr, cm^−1^): 3419, 3278, 3066, 2929, 2856, 1747, 1671, 1655, 1606, 1589, 1528, 1457, 1373, 1286, 1249, 1221, 1192, 760. HRMS (ESI) calcd for C_18_H_18_N_2_O_4_Na (M + Na)^+^: 349.1164, found: 349.1166.

*N-((3-methylphenylamino)carbonylmethyl)-2-acetoxybenzamide* (**3c**): Yield 48%, white solid, mp 156.6–157.6 °C. ^1^H NMR (400 MHz, DMSO-*d*_6_) *δ*: 9.93 (s, 1H), 8.48 (t, *J* = 5.7 Hz, 1H), 7.73 (d, *J* = 7.7 Hz, 1H), 7.57-7.52 (m, 1H), 7.44-7.35 (m, 3H), 7.20 (t, *J* = 8.1 Hz, 2H), 6.88 (d, *J* = 7.5Hz, 1H), 4.04 (d, *J* = 5.9 Hz, 2H), 2.28 (s, 3H), 2.27 (s, 3H). ^13^C NMR (100 MHz, DMSO-*d*_6_) *δ*: 169.11, 167.39, 165.32, 148.13, 138.84, 138.00, 131.66, 129.44, 128.66, 128.44, 125.93, 124.03, 123.52, 119.65, 116.33, 43.15, 21.23, 20.88. IR (KBr, cm^−1^): 3404, 3291, 3093, 2987, 2922, 1772, 1701, 1631, 1612, 1552, 1522, 1482, 1433, 1352, 1257, 1178, 756. HRMS (ESI) calcd for C_18_H_18_N_2_O_4_Na (M + Na)^+^: 349.1164, found: 349.1163.

*N-((4-methylphenylamino)carbonylmethyl)-2-acetoxybenzamide* (**3d**): Yield 49%, white solid, mp 182.4–183.7 °C. ^1^H NMR (400 MHz, DMSO-*d*_6_) *δ*: 9.94 (s, 1H), 8.49 (t, *J* = 5.8 Hz, 1H), 7.73-7.71 (m, 1H), 7.56-7.46 (m, 3H), 7.38-7.34 (m, 1H), 7.21 (d, *J* = 8.1 Hz, 1H), 7.12 (d, *J* = 8.1 Hz, 2H), 4.04 (d, *J* = 5.6 Hz, 2H), 2.27 (s, 3H), 2.25 (s, 3H). ^13^C NMR (100 MHz, DMSO-*d*_6_) *δ*: 169.07, 167.55, 165.35, 148.14, 136.05, 131.67, 131.58, 130.35, 129.46, 128.36, 126.02, 125.91, 125.22, 124.72, 123.50,43.04, 20.85, 17.80. IR (KBr, cm^−1^): 3405, 3275, 3124, 2923, 1776, 1700, 1631, 1600, 1566, 1514, 1478, 1429, 1351, 1296, 1215, 1174, 910, 756. HRMS (ESI) calcd for C_18_H_18_N_2_O_4_Na (M + Na)^+^: 349.1164, found: 349.1169.

*N-((2-methoxyphenylamino)carbonylmethyl)-2-acetoxybenzamide* (**3e**): Yield 49%, white solid, mp 80.4–81.6 °C. ^1^H NMR (400 MHz, DMSO-*d*_6_) *δ*: 9.18 (s, 1H), 8.63 (t, *J* = 5.8 Hz, 1H), 8.04 (d, *J* = 8.0 Hz, 1H), 7.73-7.70 (m, 1H), 7.57-7.53 (m, 1H), 7.41-7.39 (m, 1H), 7.23-7.19 (m, 1H), 7.10-7.03 (m, 2H), 6.96-6.89 (m, 1H), 4.08 (d, *J* = 5.9 Hz, 2H), 3.83 (s, 3H), 2.24 (s, 3H). ^13^C NMR (100 MHz, DMSO-*d*_6_) *δ*: 169.00, 167.52, 165.47, 149.03, 148.11, 131.70, 129.25, 128.37, 127.03, 125.93, 124.26, 123.55, 120.91, 120.37, 111.11, 55.72, 43.47, 20.79. IR (KBr, cm^−1^): 3404, 3325, 3076, 2965, 2839, 1763, 1689, 1661, 1605, 1524, 1489, 1351, 1174, 1191, 1029, 910, 741. HRMS(ESI) calcd for C_18_H_18_N_2_O_5_Na (M + Na)^+^: 365.1113, found: 365.1118.

*N-((3-methoxyphenylamino)carbonylmethyl)-2-acetoxybenzamide* (**3f**): Yield 51%, white solid, mp 183.8–184.9 °C. ^1^H NMR (400 MHz, DMSO-*d*_6_) *δ*: 10.03 (s, 1H), 8.49 (t, *J* = 5.7 Hz, 1H), 7.72-7.70 (m, 1H), 7.56-7.52 (m, 1H), 7.38-7.34 (m, 1H), 7.31 (s, 1H), 7.24-7.19 (m, 2H), 7.13 (d, *J* = 7.8 Hz, 1H), 7.65-6.63 (m, 1H), 4.03 (d, *J* = 5.8 Hz, 2H), 3.72 (s, 3H), 2.27 (s, 3H). ^13^C NMR (100 MHz, DMSO-*d*_6_) *δ*: 169.26, 167.62, 165.49, 159.67, 148.19, 140.13, 131.79, 129.76, 129.49, 128.51, 126.06, 123.62, 111.52, 108.81, 105.09, 55.09, 43.23, 20.95. IR (KBr, cm^−1^): 3394, 3317, 3281, 2935, 1769, 1702, 1631, 1603, 1561, 1527, 1482, 1413, 1351, 1231, 1176, 1093, 773. HRMS(ESI) calcd for C_18_H_18_N_2_O_5_Na (M + Na)^+^: 365.1113, found: 365.1115.

*N-((4-methoxyphenylamino)carbonylmethyl)-2-acetoxybenzamide* (**3g**): Yield 52%, white solid, mp 160.3–161.5 °C. ^1^H NMR (400 MHz, DMSO-*d*_6_) *δ*: 9.91 (s, 1H), 8.96-8.94 (m, 1H), 8.84 (t, *J* = 4.8 Hz, 1H), 8.61-8.54 (m, 3H), 8.41- 8.37k (m, 1H), 8.24 (d, *J* = 7.1 Hz, 1H), 7.93-7.89(m, 2H), 5.41 (d, *J* = 4.8 Hz, 2H), 4.85 (s, 3H), 3.47 (s, 3H). ^13^C NMR (100 MHz, DMSO-*d*_6_) *δ*: 169.10, 166.93, 165.23, 155.22, 148.10, 132.04, 131.64, 129.44, 128.43, 125.91, 123.50, 120.64(2C), 113.91 (2C), 55.15, 42.99, 20.88. IR (KBr, cm^−1^): 3388, 3269, 3097, 2921, 1776, 1690, 1630, 1610, 1563, 1511, 1479, 1372, 1242, 1182, 1035, 824, 756. HRMS(ESI) calcd for C_18_H_18_N_2_O_5_Na (M + Na)^+^: 365.1113, found: 365.1110.

*N-((3-chlorophenylamino)carbonylmethyl)-2-acetoxybenzamide* (**3h**): Yield 46%, white solid, mp 162.1–163.1 °C. ^1^H NMR (400 MHz, DMSO-*d*_6_) *δ*: 10.25 (s, 1H), 8.54 (t, *J* = 5.8 Hz, 1H), 7.82 (s, 1H), 7.73-7.71 (m, 1H), 7.57-7.53 (m, 1H), 7.47 (d, *J* = 7.2 Hz, 1H), 7.35 (q, *J* = 7.9 Hz, 2H), 7.22-7.20 (m, 1H), 7.13-7.11 (m, 1H), 4.05 (d, *J* = 5.8 Hz, 2H), 2.27 (s, 3H). ^13^C NMR (100 MHz, DMSO-*d*_6_) *δ*: 169.08, 167.93, 165.40, 148.13, 140.35, 133.14, 131.68, 130.56, 129.40, 128.40, 125.93, 123.53, 123.53, 123.03, 118.57, 117.48, 43.18, 20.88. IR (KBr, cm^−1^): 3393, 3323, 3201, 2923, 1782, 1691, 1631, 1604, 1524, 1481, 1351, 1240, 1187, 1096, 700. HRMS(ESI) calcd for C_17_H_15_ClN_2_O_4_Na (M + Na)^+^: 369.0618, found: 369.0621.

*N-((4-chlorophenylamino)carbonylmethyl)-2-acetoxybenzamide* (**3i**): Yield 48%, white solid, mp 166.8–167.1 °C. ^1^H NMR (400 MHz, DMSO-*d*_6_) *δ*: 10.19 (s, 1H), 8.52 (t, *J* = 5.8 Hz, 1H), 7.73-7.70(m, 1H), 7.64 (d, *J* = 8.5 Hz, 2H), 7.56-7.52 (m, 1H), 7.39-7.36 (m, 3H), 7.21 (d, *J* = 8.0 Hz, 1H), 4.05 (d, *J* = 5.7 Hz, 2H), 2.27 (s, 3H). ^13^C NMR (100 MHz, DMSO-*d*_6_) *δ*: 169.10, 167.69, 165.36, 148.12, 137.88, 131.68, 129.43, 128.75(2C), 128.42, 126.86, 125.94, 123.53, 120.65 (2C), 43.16, 20.90. IR (KBr, cm^−1^): 3313, 3280, 2981, 2926, 1774, 1660, 1631, 1612, 1553, 1494, 1442, 1351, 1184, 1080, 754. HRMS(ESI) calcd for C_17_H_15_ClN_2_O_4_Na (M + Na)^+^: 369.0618, found: 369.0620.

*N-((1-(2-Methoxyphenylamino)carbonyl)-1-phenylmethyl)-2-acetoxybenzamide* (**3j**): Yield 49%, white solid, mp 146.8–147.2 °C. ^1^H NMR (400 MHz, DMSO-*d*_6_) *δ*: 9.59 (s, 1H), 8.85 (d, *J* = 7.7 Hz, 1H), 7.94-7.92 (m, 1H), 7.74-7.72(m, 1H), 7.61-7.55 (m, 3H), 7.41-7.32 (m, 4H), 7.23 (d, *J* = 8.1 Hz, 1H), 7.09-7.02 (m, 2H), 6.93- 6.89 (m, 1H), 6.06 (d, *J* = 7.7 Hz, 1H), 3.80 (s, 3H), 2.18 (s, 3H). ^13^C NMR (100 MHz, DMSO-*d*_6_) *δ*: 168.93, 168.68, 164.46, 149.73, 148.05, 138.20, 131.79, 129.79, 128.50(2C), 128.15, 127.88, 126.76, 127.59(2C), 125.95, 124.93, 123.40, 121.73, 120.36, 111.36, 57.14, 55.76, 20.68. IR (KBr, cm^−1^): 3327, 3265, 2939, 2833, 1753, 1656, 1632, 1604, 1528, 1493, 1460, 1365, 1288, 1261, 1220, 1029, 752. HRMS(ESI) calcd for C_24_H_22_N_2_O_5_Na (M + Na)^+^: 441.1426, found: 441.1427.

*N-((1-(3-Methoxyphenylamino)carbonyl)-1-ethyl)-2-acetoxybenzamide* (**3k**): Yield 51%, transparent oil. ^1^H NMR (400 MHz, DMSO-*d*_6_) *δ*: 10.03 (s, 1H), 8.45 (d, *J* = 7.0 Hz, 1H), 7.71-7.68 (m, 1H), 7.55-7.51 (m, 1H), 7.37-7.33 (m, 2H), 7.24-7.15 (m, 3H), 6.66-6.63 (m, 3H), 4.56 (p, *J* = 7.1 Hz, 1H), 3.73 (s, 3H), 2.24 (s, 3H), 1.38 (d, *J* = 7.1 Hz, 3H). ^13^C NMR (100 MHz, DMSO-*d*_6_) *δ*: 171.13, 169.00, 164.85, 159.56, 148.05, 140.18, 131.58, 129.61, 129.53, 128.57, 125.87, 123.35, 111.52, 108.84, 105.02, 55.01, 49.65, 20.82, 18.03. IR (KBr, cm^−1^): 3375, 3286, 2937, 2833, 2654, 1752, 1697, 1631, 1605, 1546, 1453, 1381, 1288, 1246, 1158, 1042, 756. HRMS(ESI) calcd for C_19_H_20_N_2_O_5_Na (M + Na)^+^: 379.1270, found: 379.1272.

*N-(phenylcarbamoyl)-1-ethyl-2-acetoxybenzamide* (**3l**): Yield 62%, white solid, mp 125.1–127.1 °C. ^1^H NMR (400 MHz, DMSO-*d*_6_) *δ*: 10.05 (s, 1H), 8.45 (d, *J* = 7.2 Hz, 1H), 7.72-7.70k (m, 3H), 7.63 (d, *J* = 7.2 Hz, 2H), 7.55-7.51 (m, 1H), 7.37-7.30 (m, 3H), 7.20 (d, *J* = 8.2 Hz, 1H), 7.06 (t, *J* = 7.4 Hz, 1H), 4.59 (p, *J* = 7.1 Hz, 1H), 2.24 (s, 3H), 1.40 (d, *J* = 7.2 Hz, 3H). ^13^C NMR (100 MHz, DMSO-*d*_6_) *δ*: 171.06, 168.97, 164.81, 148.04, 138.99, 131.57, 129.55, 128.78(2C), 128.55, 125.85, 123.38, 123.34, 119.24(2C), 49.60, 20.80, 18.08. IR (KBr, cm^−1^): 3311, 3219, 2979, 2937, 1760, 1662, 1642, 1602, 1549, 1492, 1440, 1351, 1326, 1283, 1199, 1080, 764. HRMS(ESI) calcd for C_18_H_18_N_2_O_4_Na (M + Na)^+^: 349.1164, found: 349.1165.

*N-((1-naphthylaminocarbonyl)-1-ethyl)-2-acetoxybenzamide* (**3m**): Yield 46%, white solid, mp 132.4–133.3 °C. ^1^H NMR (400 MHz, DMSO-*d*_6_) *δ*: 10.04 (s, 1H), 8.54 (d, *J* = 7.0 Hz, 1H), 8.10-8.07 (m, 1H), 7.96-7.94 (m, 1H), 7.81-7.74 (m, 2H), 7.66 (d, *J* = 7.4 Hz, 1H), 7.56-7.49 (m, 4H), 7.39-7.37 (m, 1H), 7.22 (d, *J* = 8.0 Hz, 1H), 4.80 (p, *J* = 7.0 Hz, 1H), 2.22 (s, 3H), 1.52 (d, *J* = 7.1 Hz, 3H). ^13^C NMR (100 MHz, DMSO-*d*_6_) *δ*: 171.82, 168.93, 164.95, 148.07, 133.71, 133.31, 131.58, 129.59, 128.49, 128.12, 128.06, 126.08, 125.94, 125.83, 125.56(2C), 123.34, 122.76, 121.96, 49.47, 20.79, 18.12. IR (KBr, cm^−1^): 3264, 3053, 2973, 2924, 2812, 1752, 1658, 1631, 1608,1531, 1450, 1350, 1293, 1249, 1202, 1097, 1014, 793. HRMS(ESI) calcd for C_22_H_20_N_2_O_4_Na (M + Na)^+^: 399.1321, found: 399.1321.

#### 3.2.2. Synthesis of 2-Benzoyloxybenzamide Derivatives **5a**–**5d**

*General Procedure:* In a 50 mL round-bottom flask, *N*-((3-chloroanilino)carbonylmethyl)salicylamide (**4a**) (0.365 g, 1.2 mmol), triethylamine (0.121 g, 1.2 mmol), and 10 mL of dry dichloromethane were added. The mixture was placed in an ice-water bath, and benzoyl chloride (0.140 g, 1.0 mmol) was added dropwise while stirring. The reaction was allowed to proceed for 2 h. After the reaction was complete (monitored by TLC), the solvent was removed under reduced pressure. Distilled water (20 mL) was added, and the mixture was then extracted with dichloromethane (20 mL × 3). The combined organic layers were dried over anhydrous Na_2_SO_4_, filtered, and the solvent was removed under reduced pressure. The resulting residue was purified by column chromatography (eluent: petroleum ether/ethyl acetate = 2:1, Rf = 0.28) to obtain product **5a** as a yellow solid with a yield of 64%.

*N-((3-chlorophenylamino)carbonylmethyl)-2-benzoyloxybenzamide* (**5a**): Yield 64%, yellow solid, mp 150.9–152.5 °C. ^1^H NMR (400 MHz, DMSO-*d*_6_) *δ*: 10.14 (s, 1H), 8.76 (t, *J* = 5.9 Hz, 1H), 8.12-8.09 (m, 2H), 7.79 (s, 1H), 7.74-7.67 (m, 2H), 7.61 (t, *J* = 7.7 Hz, 1H), 7.53 (t, *J* = 7.7 Hz, 2H), 7.45-7.37 (m, 3H), 7.33 (t, *J* = 8.0 Hz, 1H), 7.11 (dd, *J* = 7.8, 2.1 Hz, 1H), 3.95 (d, *J* = 5.8 Hz, 2H). ^13^C NMR (100 MHz, DMSO-*d*_6_) *δ*: 167.75, 165.77, 164.46, 148.19, 140.30, 133.74, 133.08, 131.48, 130.42, 129.97, 129.11, 129.05, 128.96, 128.66, 126.02, 123.60, 122.92, 118.53, 117.41, 43.19. IR (KBr, cm^−1^): 3388, 3282, 3201, 2925, 1776, 1701, 1633, 1600, 1521, 1440, 1368, 1308, 1253, 1174, 1097, 755. HRMS(ESI) calcd for C_22_H_17_ClN_2_O_4_Na (M + Na)^+^: 431.0775, found: 431.0777.

*N-((4-chlorophenylamino)carbonylmethyl)-2-benzoyloxybenzamide* (**5b**): Yield 74%, white solid, mp 183.6–184.0 °C. ^1^H NMR (400 MHz, DMSO-*d*_6_) *δ*: 10.07 (s, 1H), 8.73 (t, *J* = 5.9 Hz, 1H), 8.11 (d, *J* = 6.9 Hz, 2H), 7.74-7.68 (m, 2H), 7.60-7.52 (m, 5H), 7.45-7.41 (m, 1H), 7.39-7.34 (m, 3H), 3.94 (d, *J* = 5.9 Hz, 2H). ^13^C NMR (100 MHz, DMSO-*d*_6_) *δ*: 167.51, 165.74, 164.46, 148.20, 137.82, 133.75, 131.48, 129.96, 129.10, 129.06, 128.95, 128.70, 128.62, 126.76, 126.02, 123.59, 120.59, 43.15. IR (KBr, cm^−1^): 3377, 3313, 3289, 3196, 2937, 1749, 1697, 1647, 1602, 1550, 1516, 1424, 1402, 1353, 1256, 1176, 1088, 1051, 823, 747. HRMS(ESI) calcd for C_22_H_17_ClN_2_O_4_Na (M + Na)^+^: 431.0775, found: 431.0776.

*N-(phenylcarbamoylmethyl)-2-benzoyloxybenzamide* (**5c**): Yield 45%, white solid, mp 63.8–64.8 °C. ^1^H NMR (400 MHz, DMSO-*d*_6_) *δ*: 9.93 (s, 1H), 8.73 (t, *J* = 5.9 Hz, 1H), 8.11 (d, *J* = 6.9 Hz, 2H), 7.75-7.67 (m, 2H), 7.60-7.51 (m, 5H), 7.45-7.37 (m, 2H), 7.30 (t, *J* = 7.9 Hz, 2H), 7.05 (t, *J* = 7.4 Hz, 1H), 3.95 (d, *J* = 6.0 Hz, 2H). ^13^C NMR (100 MHz, DMSO-*d*_6_) *δ*: 167.36, 165.77, 164.53, 148.23, 138.89, 133.80, 131.53, 130.01, 129.10, 129.10, 129.01, 128.75, 128.75, 126.08, 123.63, 123.26, 119.10, 43.16. IR (KBr, cm^−1^): 3412, 3304, 3071, 2944, 1736, 1672, 1651, 1598, 1533, 1480, 1445, 1351, 1269, 1165, 1063, 774. HRMS(ESI) calcd for C_22_H_18_N_2_O_4_Na (M + Na)^+^: 397.1164, found: 397.1169.

*N-((phenylcarbamoyl)-1-ethyl)-2-benzoyloxybenzamide* (**5d**): Yield 38%, white solid, mp 134.6–135.7 °C. ^1^H NMR (400 MHz, DMSO-*d*_6_) *δ*: 9.93 (s, 1H), 8.63 (d, *J* = 7.0 Hz, 1H), 8.09 (d, *J* = 7.9 Hz, 2H), 7.71-7.66 (m, 2H), 7.60-7.49 (m, 5H), 7.43-7.36 (m, 2H), 7.29 (t, *J* = 7.7 Hz, 2H), 7.04 (t, *J* = 7.3 Hz, 1H), 4.42 (p, *J* = 7.1 Hz, 1H), 1.24 (s, 3H). ^13^C NMR (100 MHz, DMSO-*d*_6_) *δ*: 171.00, 165.20, 164.42, 148.04, 138.98, 133.86, 131.43, 129.97, 129.35, 129.15, 128.97, 128.75, 128.70, 126.01, 123.39, 123.25, 119.15, 49.68, 17.64. IR (KBr, cm^−1^): 3408, 3289, 3065, 2976, 1743, 1670, 1663, 1598, 1533, 1447, 1389, 1270, 1173, 1098, 1060, 752, 704. HRMS(ESI) calcd for C_23_H_20_N_2_O_4_Na (M + Na)^+^: 411.1321, found: 411.1323.

### 3.3. Antifungal Activity Testing

The in vitro inhibition of mycelium in the agar culture medium caused by the target compounds against six phytopathogenic fungi: *Sclerotonia sclerotiorum*, *Botrytis cinerea*, *Rhizoctonia solani*, *Gibberella zeae*, *Phytophythora capsic*, and *Magnaporthe oryzae* was performed according to the standard method NY/T1156.5-2006 [25]. The antifungal activity assays adopted the drug-containing medium method with chlorothalonil used as a reference compound. The concentration of compound was 50 µg/mL. The operation procedure is detailed in our previous paper [25]. The inhibition percent was calculated according to the following equation:

*I* = [(D_1_ − D_0_)/D_1_ − 4] × 100%

where *I* is the inhibition rate, D_1_ is the average diameter of mycelia the blank test, and D_0_ is the average diameter of mycelia in the presence of compounds. Three replicates were performed for each compound.

### 3.4. Computational Methods

#### 3.4.1. Molecular Docking

To investigate the potential interactions between the newly synthesized compounds and the target protein scytalone dehydratase, molecular docking was performed in this study. The three-dimensional crystal structure of the target protein was retrieved from the Protein Data Bank (PDB, https://www.rcsb.org/ (accessed on 10 June 2026)) using the PDB ID 6STD, and the three-dimensional structures of the ligands were constructed using the MolDraw platform (https://www.moldraw.com/ (accessed on 10 June 2026)). The protein structure was preprocessed as follows: the binding pocket was first predicted using the PrankWeb platform (https://prankweb.cz/ (accessed on 10 June 2026)) to define the docking grid box; subsequently, AutoDockTools 1.5.7 was used to add hydrogen atoms to the protein, assign atomic charges, and set the grid box parameters. Ten independent docking runs were performed using AutoDock Vina 1.1.2, and the complex conformation with the highest binding affinity was selected. Finally, the ligand–receptor interactions (e.g., hydrogen bonds and hydrophobic interactions) were analyzed and visualized using Maestro viewer.

#### 3.4.2. Molecular Dynamics Simulation

To elucidate the stability of the ligand–protein interaction, molecular dynamics (MD) simulations were performed based on the optimal docking pose using GROMACS 2023.4. The geometry of the ligand was optimized at the B3LYP-D3(BJ)/def2-SVP level using Gaussian 16, followed by single-point energy calculation at the def2-TZVP level to derive MK electrostatic potential charges. RESP charges were then fitted using Multiwfn 3.8, and the GAFF force field parameters were assigned, with the ligand topology generated by Sobtop V2026.1.16 [28]. The protein was described with the Amber14sb force field, solvated in a TIP3P water model, and the system was neutralized with Na^+^/Cl^−^ ions. After energy minimization using the steepest descent algorithm, the system was equilibrated under NVT and NPT conditions (100 ps each). Production MD was run for 200 ns with a 2 fs time step, and trajectory frames were saved every 10 ps. Trajectory analysis afforded root-mean-square deviation (RMSD) values to monitor the conformational stability of the complex.

## 4. Conclusions

We have prepared a series of 2-acyloxybenzamide compounds in 38–74% yield. Antifungal activity evaluation revealed that compound **3i** exhibited 100.0% inhibition rate against *M. oryzae*, while compounds **3d** and **3j** showed inhibition rates of 80.6%, 86.5%, respectively, which are all higher than that of the control fungicide chlorothalonil (53.8%). Compounds **3b**, **3j** and **3m** displayed 90.0%, 83.3%, and 80.0% inhibition against *P. capsici*, respectively. The EC_50_ values of compounds **3b** and **3i** were 3.90 and 8.21 μg/mL, respectively. Molecular docking and MD simulations studies further suggested that SCD enzyme may act as a potential target for compound **3i** and its related analogues. These findings provide valuable insights for rational design of future antifungal agents.

## Figures and Tables

**Table 1:** Yields of intermediates **1**, **2**, and target compounds **3**.

Entry	R^1^	R^2^	1 (Yield/%)	2 (Yield/%)	3 (Yield/%)
1	H	C_6_H_5_	**1a** (85)	**2a** (86)	**3a** (48)
2	H	2-CH_3_C_6_H_4_	**1b** (86)	**2b** (84)	**3b** (46)
3	H	3-CH_3_C_6_H_4_	**1c** (89)	**2c** (85)	**3c** (48)
4	H	4-CH_3_C_6_H_4_	**1d** (91)	**2d** (87)	**3d** (49)
5	H	2-CH_3_OC_6_H_4_	**1e** (84)	**2e** (82)	**3e** (49)
6	H	3-CH_3_OC_6_H_4_	**1f** (88)	**2f** (85)	**3f** (51)
7	H	4-CH_3_OC_6_H_4_	**1g** (90)	**2g** (86)	**3g** (52)
8	H	3-ClC_6_H_4_	**1h** (80)	**2h** (83)	**3h** (46)
9	H	4-ClC_6_H_4_	**1i** (83)	**2i** (86)	**3i** (48)
10	C_6_H_5_	2-CH_3_OC_6_H_4_	**1j** (84)	**2j** (84)	**3j** (49)
11	CH_3_	3-CH_3_OC_6_H_4_	**1k** (84)	**2k** (84)	**3k** (51)
12	CH_3_	C_6_H_5_	**1l** (83)	**2l** (87)	**3l** (62)
13	CH_3_	1-Naph	**1m** (70)	**2m** (61)	**3m** (46)

**Table 2:** Yields of intermediates **4** and target compounds **5**.

Entry	R^1^	R^2^	4 (Yield/%)	5 (Yield/%)
1	H	3-ClC_6_H_4_	**4a** (57)	**5a** (64)
2	H	4-ClC_6_H_4_	**4b** (63)	**5b** (74)
3	H	C_6_H_5_	**4c** (51)	**5c** (45)
4	CH_3_	C_6_H_5_	**4d** (54)	**5d** (38)

**Table 3:** Antifungal activity of compounds **3** and **5** (inhibitory rate/%) ^a^.

Compound	*G. zeae*	*P. capsici*	*S. sclerotiorum*	*B. cinerea*	*R. solani*	*M. oryzae*
**3a**	25.0	11.1	6.1	29.3	10.6	4.5
**3b**	44.8	90.0	23.5	35.7	48.2	64.7
**3c**	48.3	56.7	56.9	7.1	42.9	64.7
**3d**	44.8	76.7	51.0	32.1	53.6	80.6
**3e**	35.7	54.1	58.5	42.9	41.5	30.8
**3f**	41.4	13.3	29.4	7.1	35.7	52.9
**3g**	37.9	30.0	29.4	7.1	17.9	52.9
**3h**	51.7	53.3	56.9	39.3	39.3	52.9
**3i**	44.8	46.7	58.8	39.3	50.0	100.0
**3j**	51.7	83.3	25.5	25.0	48.2	86.5
**3k**	27.6	30.0	33.3	17.9	21.4	52.9
**3l**	41.4	53.3	58.8	42.9	44.6	74.7
**3m**	51.7	80.0	49.0	67.9	73.2	74.7
**5a**	33.3	32.3	72.5	23.8	23.8	13.9
**5b**	22.2	32.3	50.0	23.8	23.8	11.1
**5c**	4.8	33.3	22.6	18.5	18.5	5.3
**5d**	19.0	5.6	6.5	37.0	37.0	5.3
Chlorothalonil ^b^	73.1	89.3	96.2	94.6	92.6	53.8

^a^ Measured at the concentration of 50 μg/mL. ^b^ Chlorothalonil used as reference compound.

**Table 4:** EC_50_ value of compounds **3i**.

Compound	EC_50_ (μg/mL) Against *P. capsici*	EC_50_ (μg/mL) Against *M. oryzae*
**3b**	3.90	/
**3i**	/	8.21
Chlorothalonil	3.31	33.56

## Data Availability

The data that support the findings of this study are available from the corresponding authors upon reasonable request.

## Supplementary Materials

The following supporting information can be downloaded at https://www.mdpi.com/article/10.3390/molecules31132261/s1, the synthesis of compounds **2** and **4**; ^1^H NMR, ^13^C NMR, HRMS and IR spectra of Compound **3**, **5**; Molecular docking studies of compounds **3a** and **3h** with the SCD enzyme.
