# Supplementary material for: Synthesis, Antifungal Activity and Molecular Docking Studies of Novel 2-Acyloxybenzamides
Source: Molecules. 2026 Jun 26;31(13):2261. doi: 10.3390/molecules31132261 (PMC13363497; doi:10.3390/molecules31132261)
Supplement: Supplementary file 1 [file molecules-31-02261-s001.zip › molecules-4354474-supplementary.pdf]

Electronic Supplementary Material (ESI)

Synthesis, Antifungal Activity and Molecular Docking Studies of Novel 2-Acyloxybenzamides

Zilong Tang [1,*], Qianyi He [1], Minyi Yao [1], Ying Ye [1], Jiangtao Cai [2], Yichao Wan [1,*] and Lifen Peng [1]

[1] Key Laboratory of Theoretical Organic Chemistry and Functional Molecule of Ministry of Education, School of Chemistry and Chemical Engineering, Hunan University of Science and Technology, Xiangtan 411201, China; 25300606106@mail.hnust.edu.cn (Q.H.); 22010601047@mail.hnust.edu.cn (M.Y.); 2406070328@mail.hnust.edu.cn (Y.Y.); 1060137@hnust.edu.cn (L.P.)

[2] School of Life Sciences and Health, Hunan University of Science and Technology, Xiangtan 411201, China; 24010901032@mail.hnust.edu.cn

* Correspondence: zltang@hnust.edu.cn (Z.T.); 07wanyichao@hnust.edu.cn (Y.W.)

Table of contents

1.Synthesis of compound **2**.........2

2.Synthesis of compound **4**.........4

3.^{1}H NMR, ^{13}C NMR, HRMS and IR spectra of Compound **3**, **5**.........5

4.Molecular docking studies of compounds **3a** and **3h** with the SCD enzyme.........39

1. Synthesis of compound **2**

General Procedure for 2a: To a solution of *N*-Phenyl-2-Boc-glycinamide **1a** (0.26 g, 1.0 mmol, 1.0 equiv) in anhydrous CH_2Cl_2 was added trifluoroacetic acid (0.59 g, 5.2 mmol, 5.0 equiv) under nitrogen at 0 °C. The mixture was stirred at room temperature for 12 hours. After removal of the solvent under reduced pressure, the residue was dissolved in CH_2Cl_2, washed with 10% aqueous sodium carbonate solution, dried over anhydrous Na_2SO_4, and concentrated to afford amide **2a** in 86% yield.
Compounds **2b–2m** were synthesized according to the general procedure described for **2a**.

N-phenylglutamylamide (**2a**): Yield 86%. ^{1}H NMR (400 MHz, $CDCl_3$) δ: 9.41 (s, 1H), 7.57 (d, *J* = 8.4 Hz, 2H), 7.31 (t, *J* = 7.9 Hz, 2H), 7.09 (t, *J* = 7.5 Hz, 1H), 3.43 (s, 2H), 1.92 (s, 2H). ^{13}C NMR (100 MHz, $CDCl_3$) δ: 171.02, 137.66, 129.03(2C), 124.22, 119.56(2C), 45.10.
*N-(2-methylphenylamino)*glutamylamide (**2b**): Yield 84%. ^{1}H NMR (400 MHz, $CDCl_3$) δ: 9.50 (s, 1H), 8.09 (dd, *J* = 8.1, 1.3 Hz, 1H), 7.23 - 7.16 (m, 2H), 7.03 (td, *J* = 7.5, 1.3 Hz, 1H), 3.47 (s, 2H), 2.28 (s, 3H), 1.67 (s, 2H). ^{13}C NMR (100 MHz, $CDCl_3$) δ: 170.78, 135.88, 130.38, 127.73, 126.84, 124.40, 121.17,45.36, 17.69.
*N-(3-methylphenylamino)*glutamylamide (**2c**): Yield 85%.^{1}H NMR (400 MHz, $CDCl_3$) δ: 9.36 (s, 1H), 7.40 - 7.35 (m, 2H), 7.17 (t, *J* = 7.8 Hz, 1H), 6.89 (dd, *J* = 7.7, 1.7 Hz, 1H), 3.37 (s, 2H), 2.30 (s, 3H), 1.76 (s, 2H). ^{13}C NMR (100 MHz, $CDCl_3$) δ: 171.01, 138.79, 137.55, 128.73, 124.88, 120.10, 116.56, 45.07, 21.41.
N-(4-methylphenylamino)glutamylamide (**2d**): Yield 87%. ^{1}H NMR (400 MHz, $CDCl_3$) δ: 9.31 (s, 1H), 7.48 - 7.45 (m, 2H), 7.11 (d, *J* = 8.3 Hz, 2H), 3.42 (s, 2H), 2.30 (s, 3H), 1.61 (s, 2H). ^{13}C NMR (100 MHz, $CDCl_3$) δ: 170.82, 135.21, 133.73, 129.53(2C), 119.56(2C), 45.18, 20.92.
N-(2-methoxyphenyl)glutamylamide (**2e**): Yield 82%. ^{1}H NMR (400 MHz, $CDCl_3$) δ: 9.71 (s, 1H), 8.41 (d, *J* = 7.9 Hz, 1H), 7.06 - 6.87 (m, 3H), 3.88 (s, 3H), 3.50 (s, 2H), 1.90 (s, 2H). ^{13}C NMR (100 MHz, $CDCl_3$) δ: 170.79, 148.46, 127.44, 123.81, 119.71, 110.12, 55.79, 45.66.
N-(3-methoxyphenyl)glutamylamide (**2f**): Yield 85%. ^{1}H NMR (400 MHz, $CDCl_3$) δ: 9.39 (s, 1H), 7.34 (t, *J* = 2.3 Hz, 1H), 7.19 (t, *J* = 8.1 Hz, 1H), 7.05 (dd, *J* = 7.9, 2.0 Hz, 1H), 6.64 (dd, *J* = 8.2, 2.5 Hz, 1H), 3.78 (s, 3H), 3.42 (s, 2H), 1.76 (s, 2H). ^{13}C NMR (100 MHz, $CDCl_3$) δ: 170.75, 160.17, 138.90, 129.68, 111.70, 109.95, 105.17, 55.30, 45.13.
N-(4-methoxyphenyl)glutamylamide (**2g**): Yield 86%. ^{1}H NMR (400 MHz, $CDCl_3$) δ: 9.26 (s, 1H), 7.56 - 7.39 (m, 2H), 6.89 - 6.77 (m, 2H), 3.76 (s, 3H), 3.42 (s, 2H), 1.65 (s, 2H). ^{13}C NMR (100 MHz, $CDCl_3$) δ: 170.69, 156.29, 131.00, 121.21(2C), 114.18(2C), 55.52, 45.11.
N-(3-chlorophenyl)glutamylamide (**2h**): Yield 83%. ^{1}H NMR (400 MHz, $CDCl_3$) δ: 9.46 (s, 1H), 7.69 (t, *J* = 2.1 Hz, 1H), 7.41 (dd, *J* = 8.2, 2.1 Hz, 1H), 7.21 (t, *J* = 8.1 Hz, 1H), 7.04 (dd, *J* = 7.8, 2.0 Hz, 1H), 3.43 (s, 2H), 1.63 (s, 2H). ^{13}C NMR (100 MHz, $CDCl_3$) δ: 171.04, 138.92, 134.75, 130.12, 124.26, 119.58, 117.48, 45.16.
N-(4-chlorophenyl)glutamylamide (**2i**): Yield 83%. ^{1}H NMR (400 MHz, $CDCl_3$) δ: 9.41 (s, 1H), 7.49 (d, *J* = 8.5 Hz, 2H), 7.26 (s, 5H), 7.22 (d, *J* = 8.4 Hz, 2H), 3.40 (s, 2H), 1.77 (s, 2H). ^{13}C NMR (100 MHz, $CDCl_3$) δ: 170.99, 136.33, 129.16, 129.11(2C), 120.78(2C), 45.11.
N-(2-methoxyphenyl)-2-phenylglycine (**2j**): Yield 84%. ^{1}H NMR (400 MHz, $CDCl_3$) δ: 9.79 (s, 1H), 8.41 (dd, *J* = 8.1, 1.7 Hz, 1H), 7.48 - 7.46 (m, 2H), 7.38 - 7.35 (m, 2H), 7.34 - 7.30 (m, 1H), 7.04 (td, *J* = 7.8, 1.7 Hz, 1H),

6.94 (td, J = 7.8, 1.4 Hz, 1H), 6.88 (dd, J = 8.1, 1.4 Hz, 1H), 4.65 (s, 1H), 3.89 (s, 3H), 2.04 (s, 2H). ^{13}C NMR (100 MHz, $CDCl_3$) δ: 171.09, 148.47, 140.93, 128.92(2C), 128.10, 127.44, 127.02(2C), 123.84, 121.02, 119.58, 110.05, 60.86, 55.80.

N-(3-methoxyphenyl)alanylamine (**2k**): Yield 84%. ^{1}H NMR (400 MHz, $CDCl_3$) δ: 9.45 (s, 1H), 7.32 (d, J = 2.4 Hz, 1H), 7.13 (td, J = 8.1, 2.6 Hz, 1H), 6.98 (dd, J = 7.9, 2.0 Hz, 1H), 6.58 (dd, J = 8.2, 2.8 Hz, 1H), 3.72 (t, J = 3.2 Hz, 3H), 3.54 - 3.48 (m, 1H), 1.76 (dd, J = 8.7, 4.2 Hz, 3H), 1.35 -1.31 (m, 3H),. ^{13}C NMR (100 MHz, $CDCl_3$) δ: 174.07, 160.11, 139.05, 129.60, 111.64, 109.94, 105.01, 55.24, 51.16, 21.49.

N-phenylalanylamine (**2l**): Yield 87%. ^{1}H NMR (400 MHz, $CDCl_3$) δ: 9.47 (s, 1H), 7.60 - 7.51 (m, 2H), 7.34 - 7.29 (m, 2H), 7.11 - 7.06 (m, 1H), 3.59 (q, J = 7.0 Hz, 1H), 1.71 (s, 2H), 1.41 (d, J = 7.0 Hz, 3H). ^{13}C NMR (100 MHz, $CDCl_3$) δ: 173.98, 137.89, 129.05(2C), 124.12, 119.49(2C), 51.25, 21.67.

N-(1-Naphthyl)alanylamine (**2m**): Yield 61%. ^{1}H NMR (400 MHz, $CDCl_3$) δ: 8.07 - 8.00 (m, 2H), 7.95 (dd, J = 8.0, 1.6 Hz, 1H), 7.74 (d, J = 8.2 Hz, 1H), 7.60 - 7.50 (m, 3H), 3.68 (q, J = 7.0 Hz, 1H), 1.40 (d, J = 7.0 Hz, 3H). ^{13}C NMR (100 MHz, $CDCl_3$) δ: 175.15, 133.75, 133.31, 128.42(2C), 126.77, 126.07(2C), 125.77(2C), 124.61, 121.59, 119.34, 51.12, 21.52.

2. Synthesis of compound **4**

*General Procedure for **4a*** : To a mixture of N-((3-chlorophenylamino)carbonylmethyl)salicylamide (**2h**) (0.184 g, 1.0 mmol), salicylic acid (0.166 g, 1.2 mmol), DMAP (0.013 g, 0.1 mmol), and EDCl (0.230 g, 1.2 mmol) in dry CH_2Cl_2 (10 mL) was added triethylamine (0.122 g, 1.2 mmol) dropwise at 0 °C under nitrogen. The mixture was stirred at room temperature for 4 h, then concentrated under reduced pressure. The residue was diluted with water (20 mL), extracted with CH_2Cl_2 (3 × 20 mL), dried over anhydrous Na_2SO_4, and concentrated. Purification by column chromatography (petroleum ether/ethyl acetate = 3:1) afforded **4a** in 57% yield.

Compounds **4b–4d** were synthesized according to the general procedure described for **4a**.

N-((3-chlorophenylamino)carbonylmethyl)salicylamide (**4a**): Yield 57%. ^{1}H NMR (400 MHz, DMSO-*d*$_6$) δ: 12.20 (s, 1H), 10.34 (s, 1H), 9.19 (t, *J* = 5.7 Hz, 1H), 7.91 (dd, *J* = 8.0, 1.7 Hz, 1H), 7.82 (d, *J* = 2.1 Hz, 1H), 7.48 - 7.41 (m, 2H), 7.34 (t, *J* = 8.1 Hz, 1H), 7.11 (d, *J* = 5.8 Hz, 1H), 6.95 - 6.90 (m, 2H), 4.14 (d, *J* = 5.6 Hz, 2H). ^{13}C NMR (100 MHz, DMSO-*d*$_6$) δ: 168.78, 167.85, 159.49, 140.34, 133.86, 133.21, 130.56, 128.52, 123.13, 118.90, 118.71, 117.59, 117.38, 115.65, 43.16.

N-((4-chlorophenylamino)carbonylmethyl)salicylamide(**4b**): Yield 63%. ^{1}H NMR (400 MHz, DMSO-*d*$_6$) δ: 12.20 (s, 1H), 10.27 (s, 1H), 9.18 (t, *J* = 5.7 Hz, 1H), 7.91 (dd, *J* = 8.0, 1.7 Hz, 1H), 7.64 (d, *J* = 8.8 Hz, 2H), 7.44 - 7.35 (m, 3H), 6.95 – 6.90 (m, 2H), 4.14 (d, *J* = 5.6 Hz, 2H). ^{13}C NMR (100 MHz, DMSO-*d*$_6$) δ: 168.70, 167.53, 159.46, 137.83, 133.79, 128.72, 128.46, 126.92, 120.72, 118.83, 117.33, 115.61, 43.06.

N-(phenylcarbamoylmethyl)salicylamide(**4c**): Yield 51%. ^{1}H NMR (400 MHz, DMSO-*d*$_6$) δ: 12.23 (s, 1H), 10.14 (s, 1H), 9.18 (t, *J* = 5.7 Hz, 1H), 7.92 (dd, *J* = 8.0, 1.7 Hz, 1H), 7.61 (d, *J* = 7.5 Hz, 2H), 7.42 (td, *J* = 8.3, 7.8, 1.7 Hz, 1H), 7.31 (t, *J* = 7.9 Hz, 2H), 7.05 (t, *J* = 7.3 Hz, 1H), 6.93 (t, *J* = 8.5 Hz, 2H), 4.14 (d, *J* = 5.6 Hz, 2H). ^{13}C NMR (101 MHz,) δ 168.62, 167.30, 159.41, 138.87, 133.78, 128.82(2C), 128.46, 123.34, 119.14(2C), 118.84, 117.31, 115.65, 43.01.

N-((phenylcarbamoyl)-1-ethyl)salicylamide(**4d**): Yield 54%. ^{1}H NMR (400 MHz, DMSO-*d*$_6$) δ: 12.09 (s, 1H), 10.15 (s, 1H), 9.01 (d, *J* = 6.9 Hz, 1H), 8.00 (dd, *J* = 7.9, 1.7 Hz, 1H), 7.66 - 7.58 (m, 2H), 7.40 (td, *J* = 7.7, 1.7 Hz, 1H), 7.31 (t, *J* = 7.7 Hz, 2H), 7.06 (t, *J* = 7.4 Hz, 1H), 6.92 (t, *J* = 8.0 Hz, 2H), 4.68 (p, *J* = 7.0 Hz, 1H), 1.46 (d, *J* = 7.0 Hz, 3H). ^{13}C NMR (100 MHz, DMSO-*d*$_6$) δ: 170.99, 167.67, 159.06, 138.95, 133.66, 129.00, 128.76(2C), 123.41, 119.29(2C), 118.81, 117.18, 115.95, 49.55, 18.24.

3. ^{1}H and ^{13}C NMR spectra of Compounds **3**, **5**.

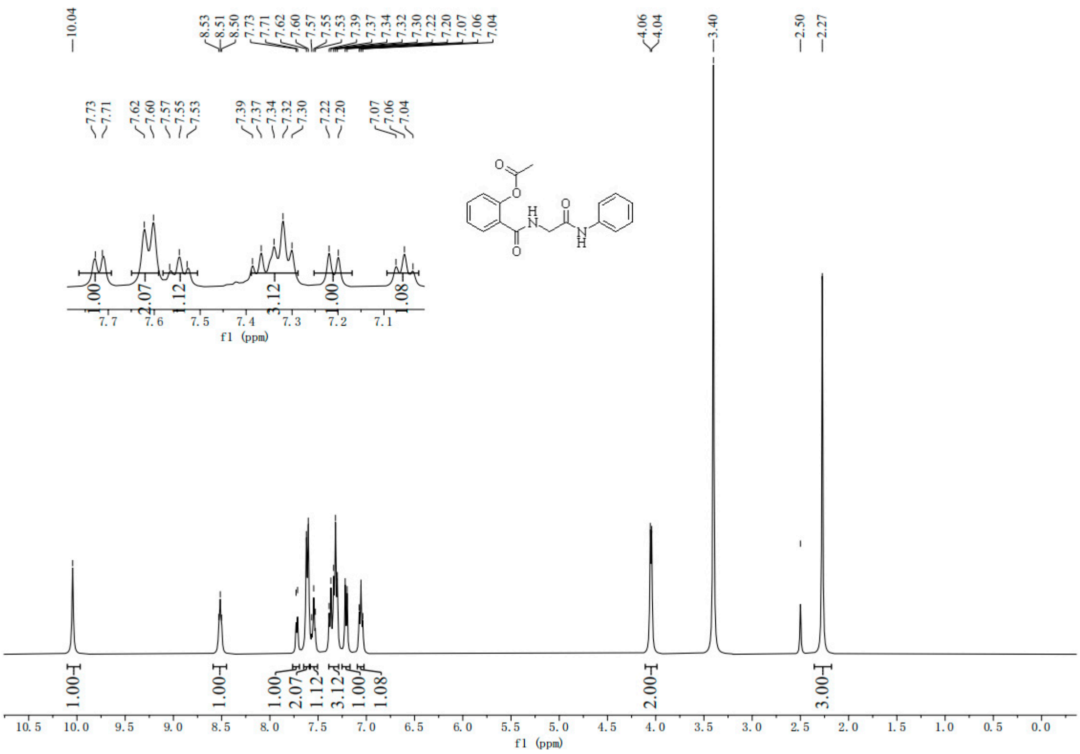

^{1}H NMR of compound **3a** (DMSO-d_6)

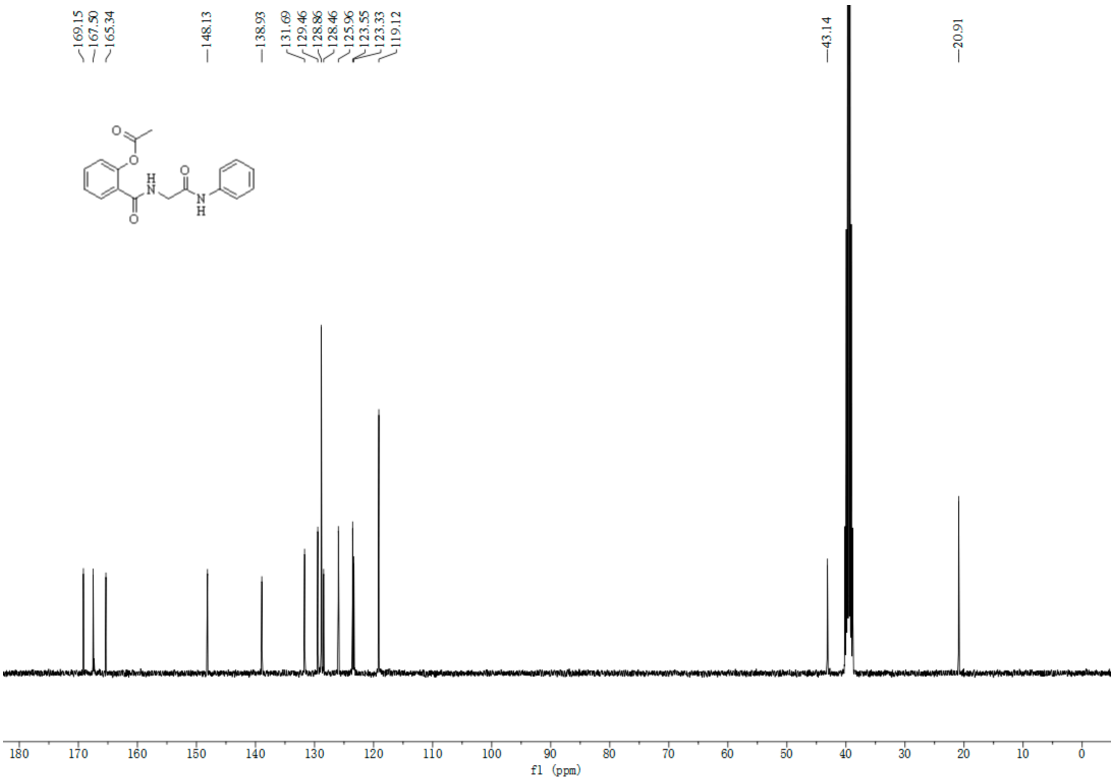

^{13}C NMR of compound **3a** (DMSO-d_6)

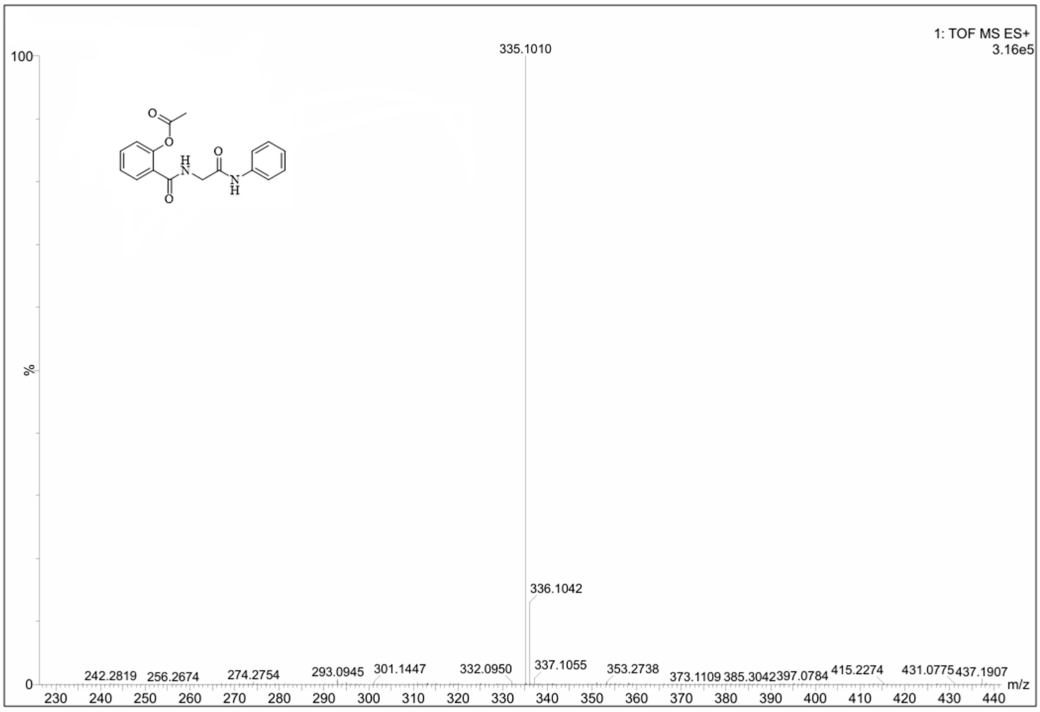

HRMS of compound **3a**

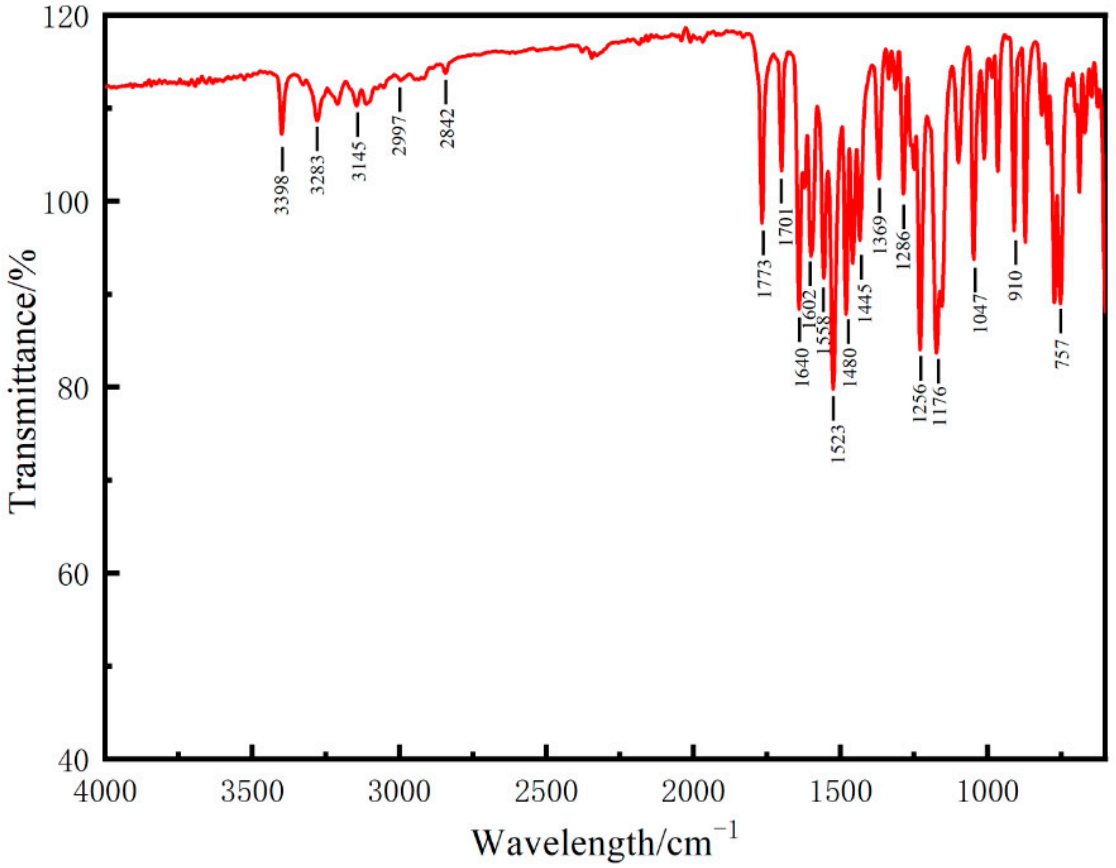

IR of compound **3a**

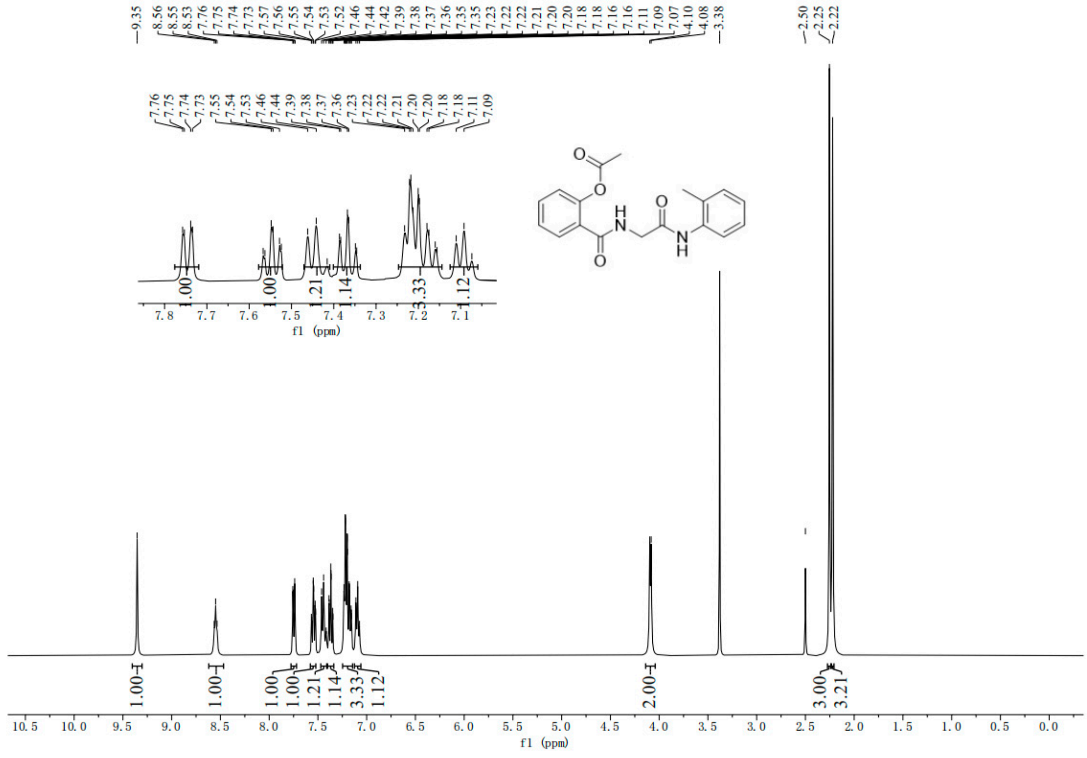

^{1}H NMR of compound **3b** (DMSO-d_6)

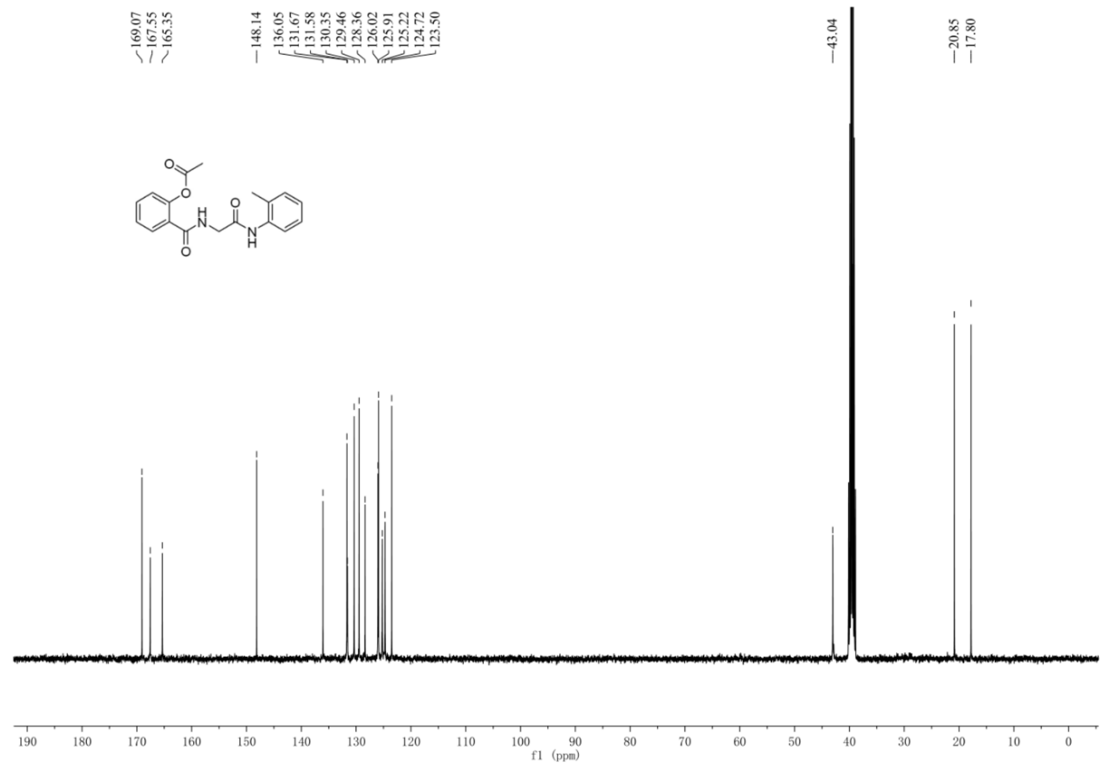

^{13}C NMR of compound **3b** (DMSO-d_6)

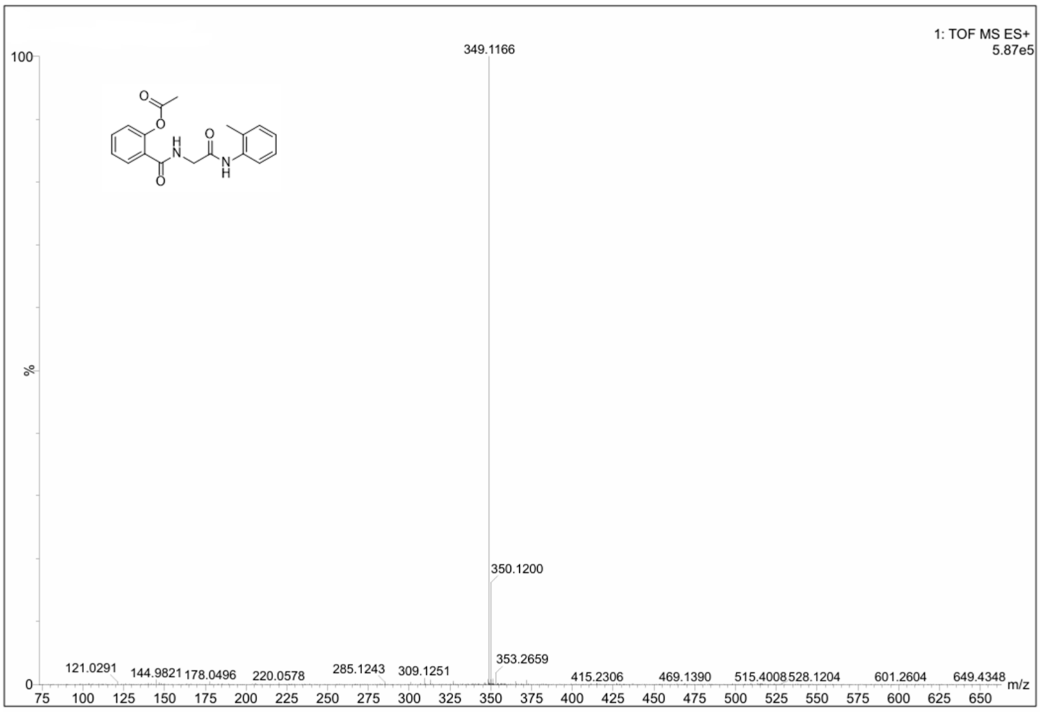

HRMS of compound **3b**

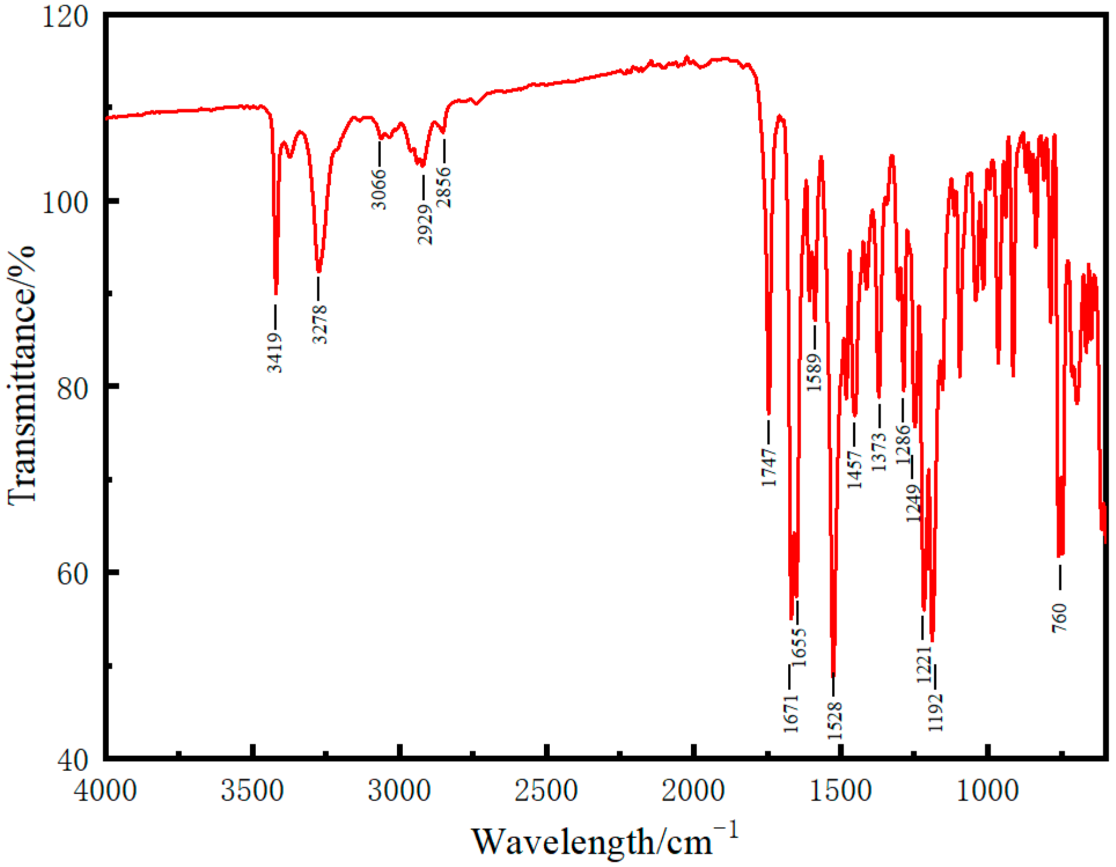

IR of compound **3b**

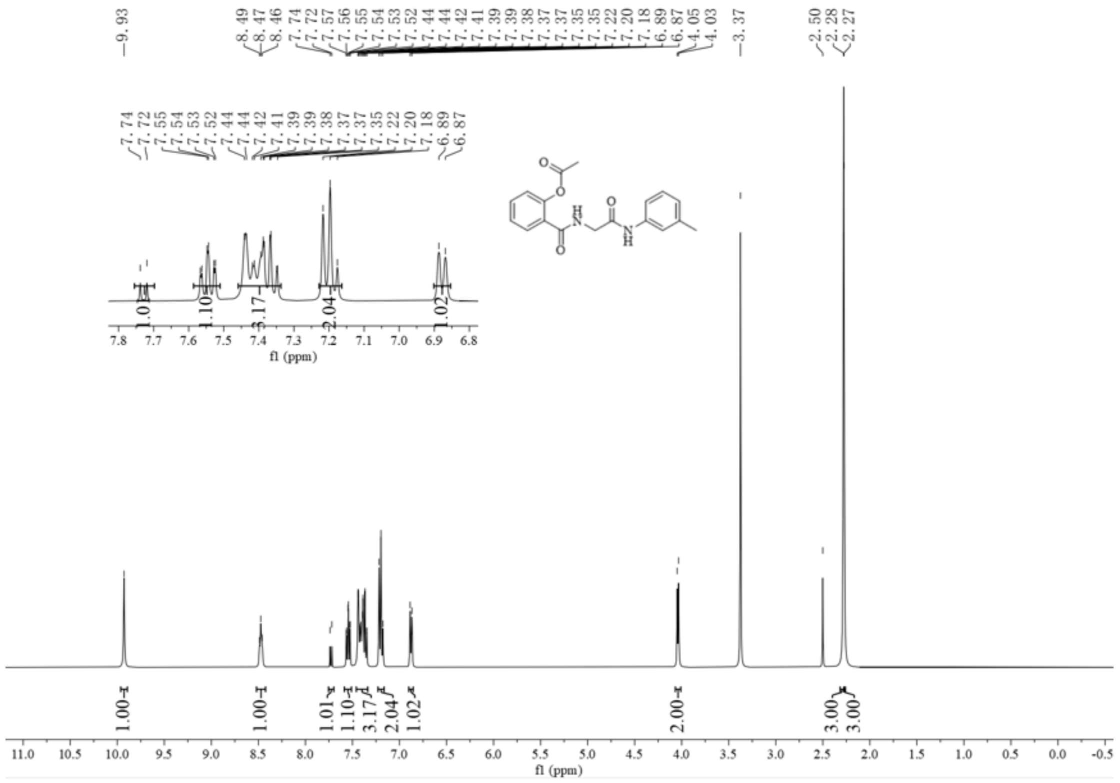

^{1}H NMR of compound **3c** (DMSO-d_6)

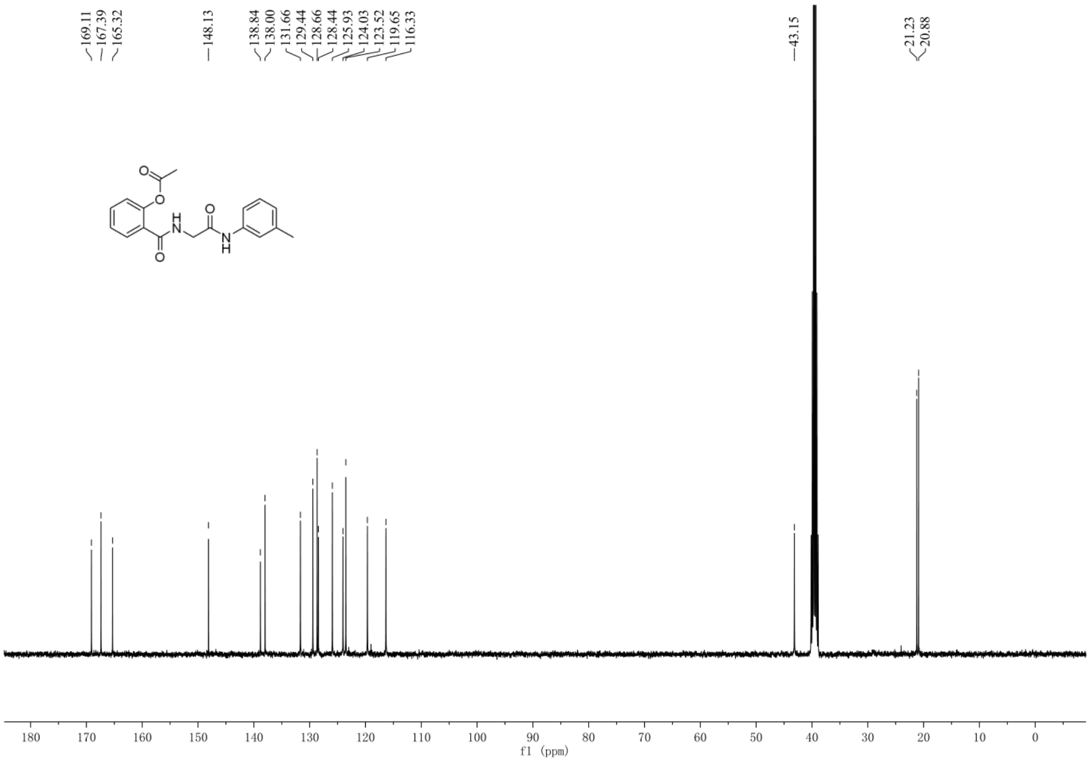

^{13}C NMR of compound **3c** (DMSO-d_6)

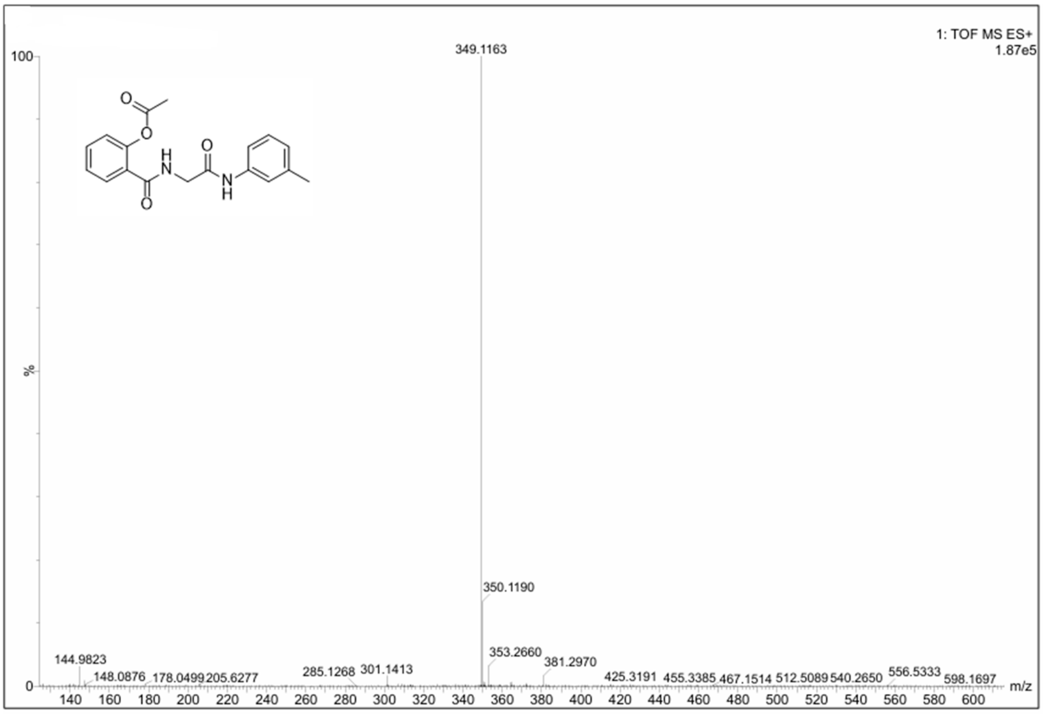

HRMS of compound **3c**

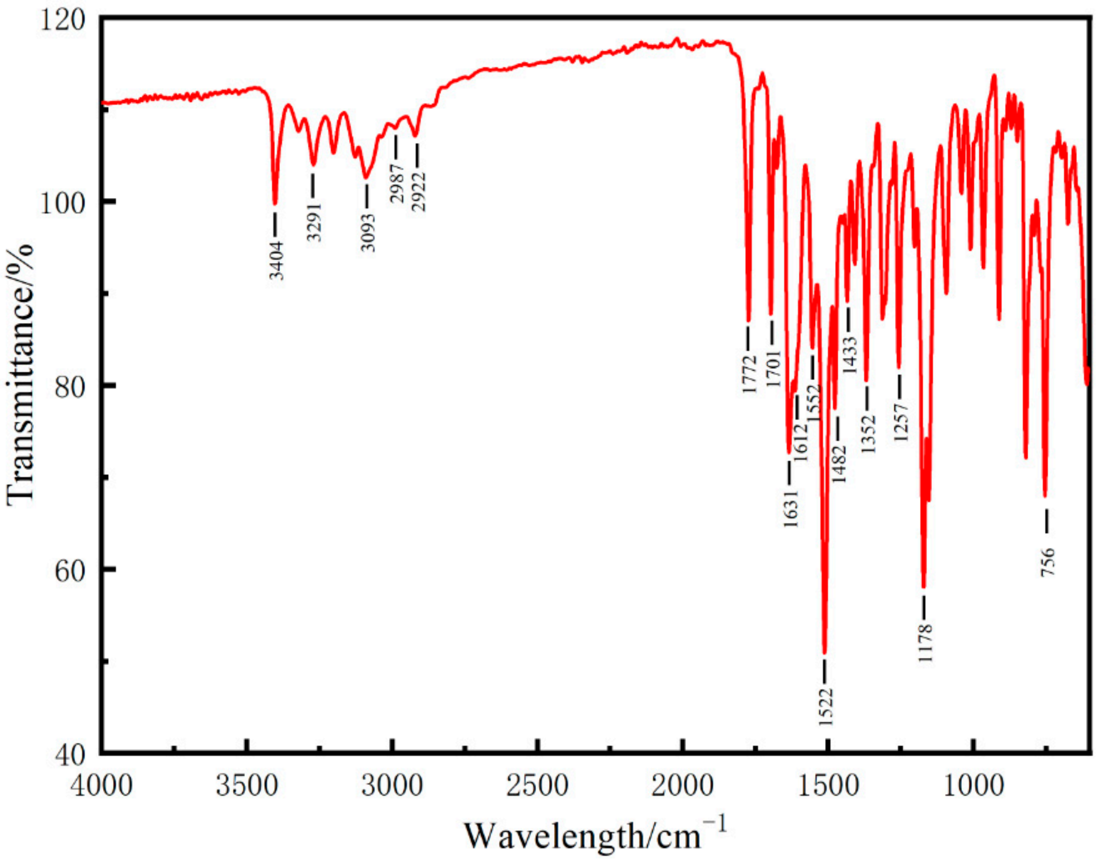

IR of compound **3c**

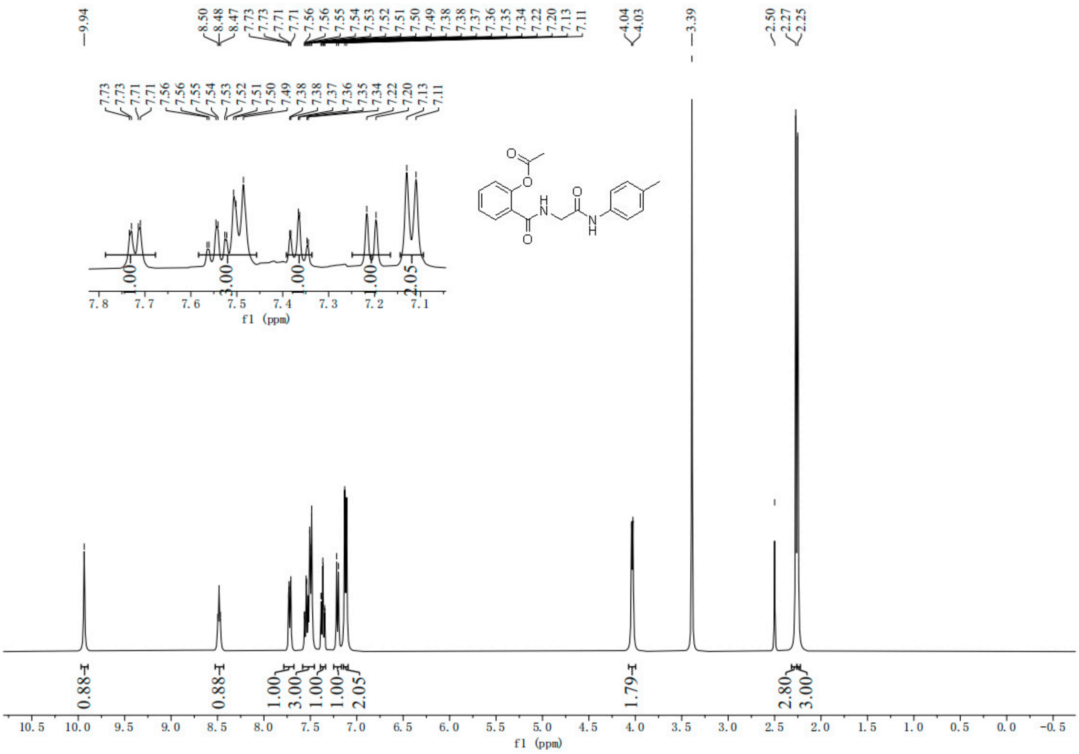

^{1}H NMR of compound **3d** (DMSO-d_6)

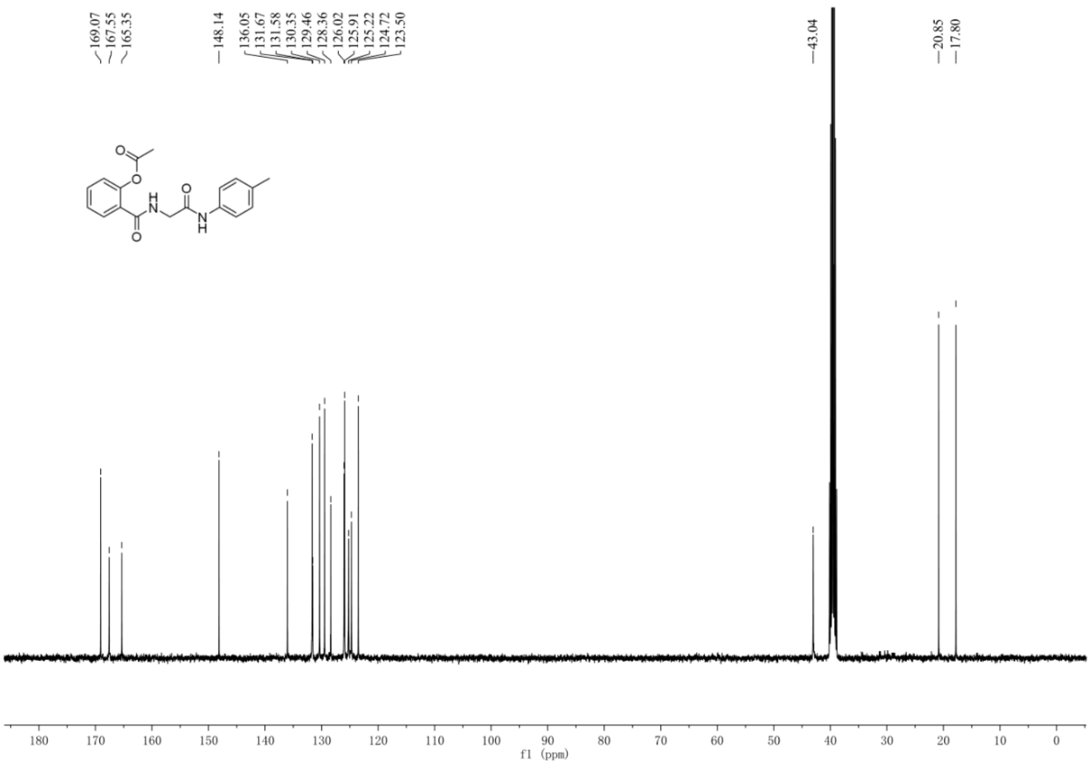

^{13}C NMR of compound **3d** (DMSO-d_6)

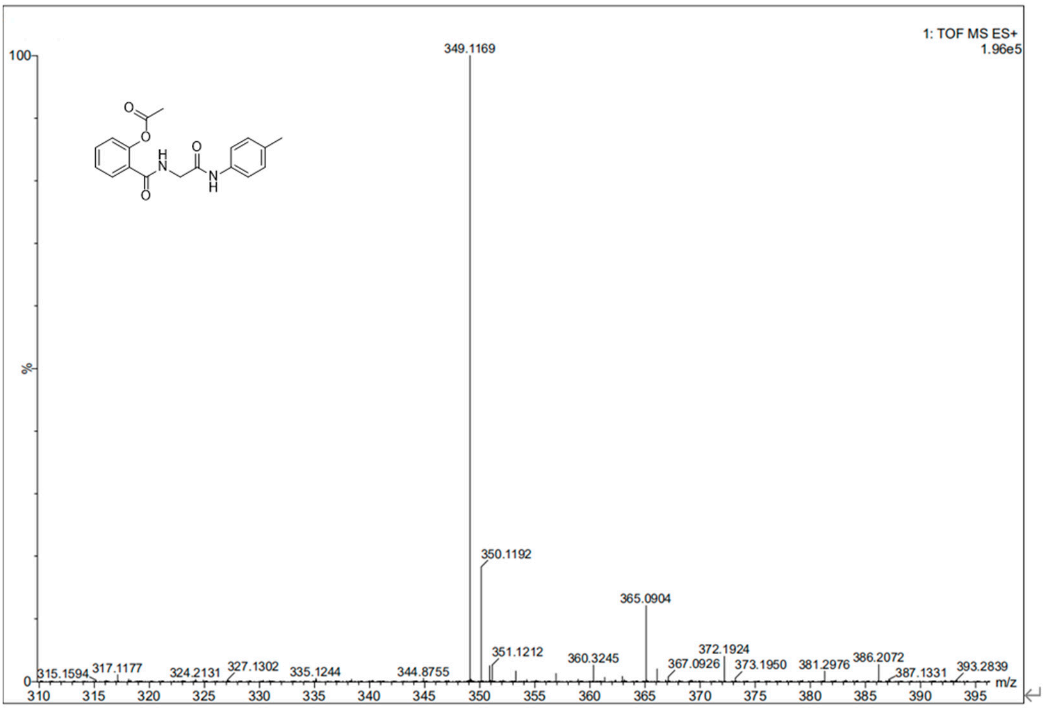

HRMS of compound **3d**

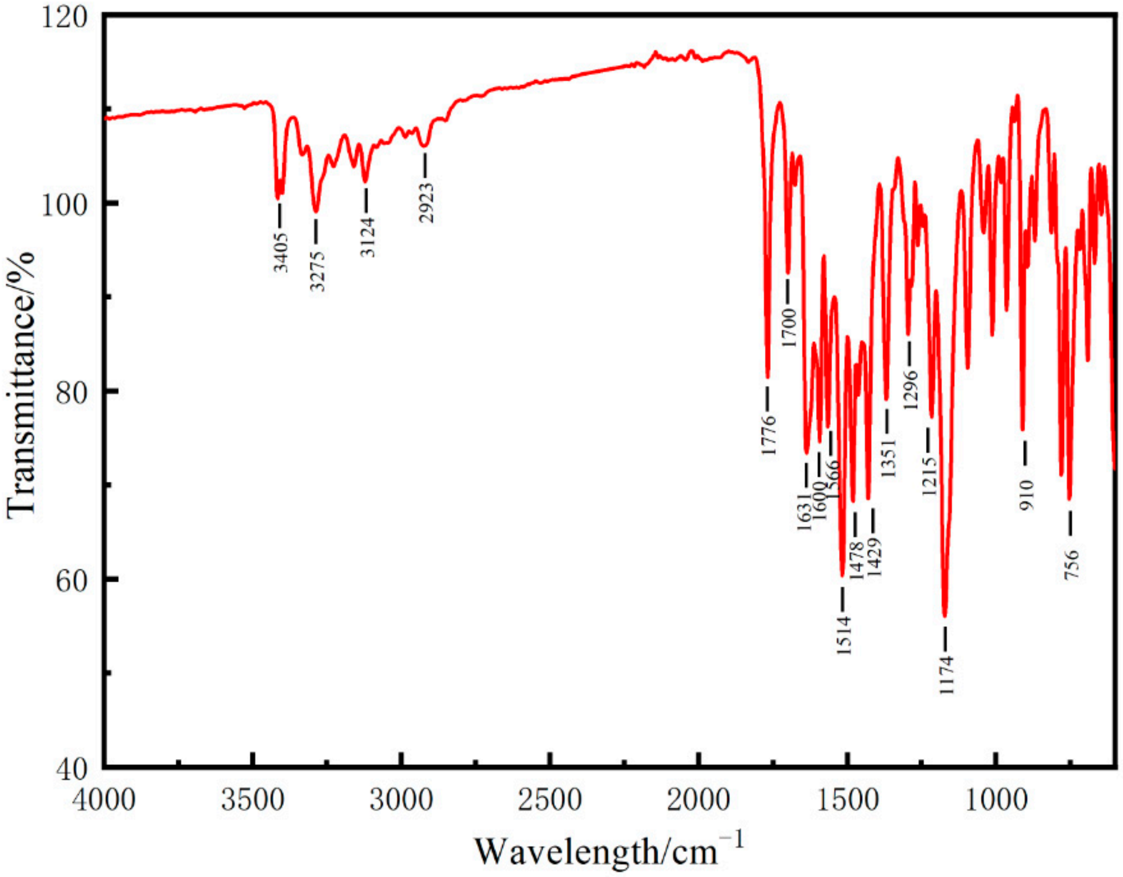

IR of compound **3d**

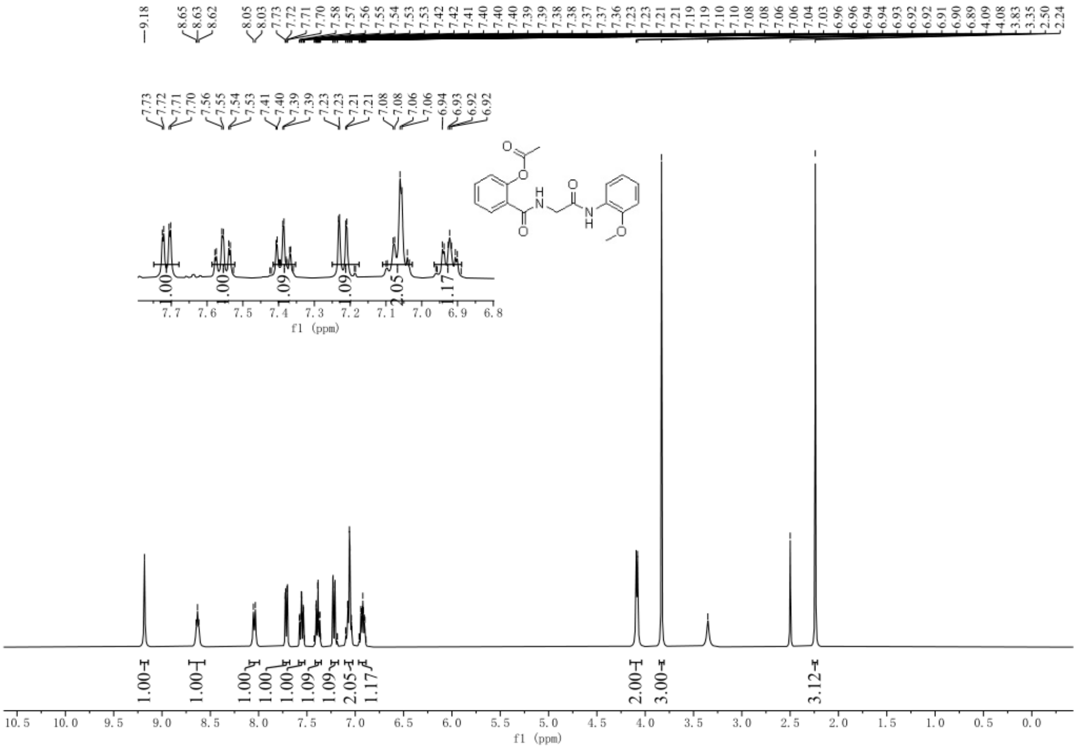

^{1}H NMR of compound **3e** (DMSO-d_6)

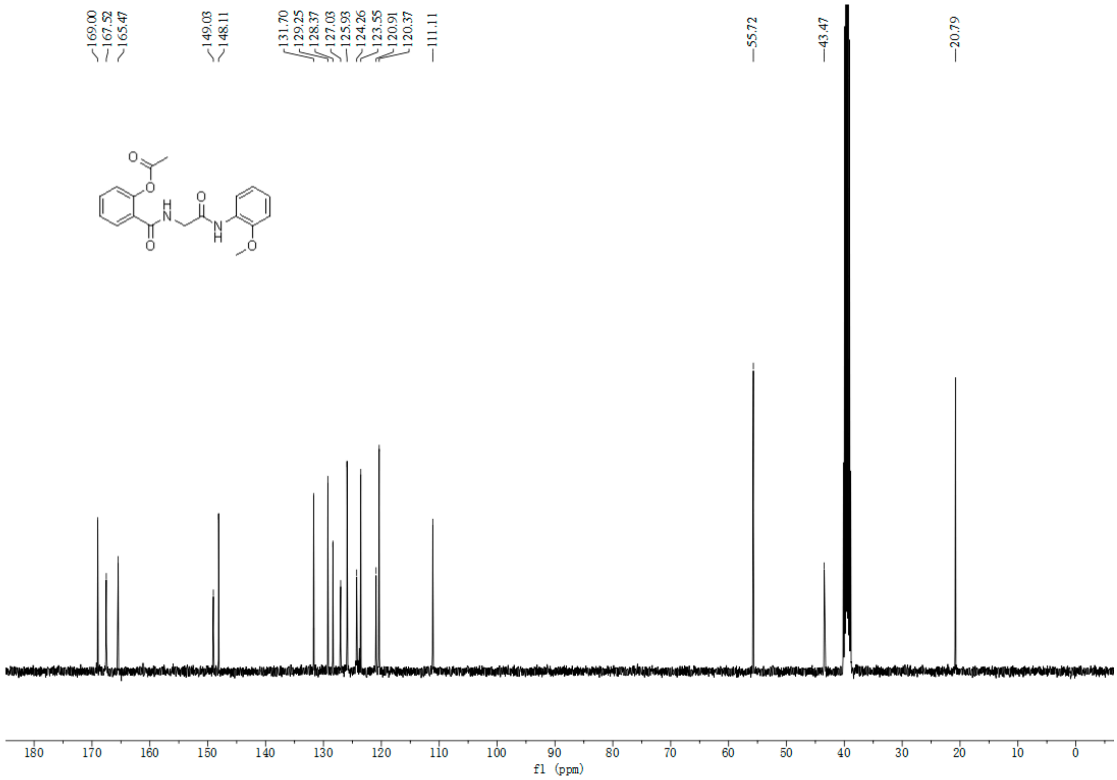

^{13}C NMR of compound **3e** (DMSO-d_6)

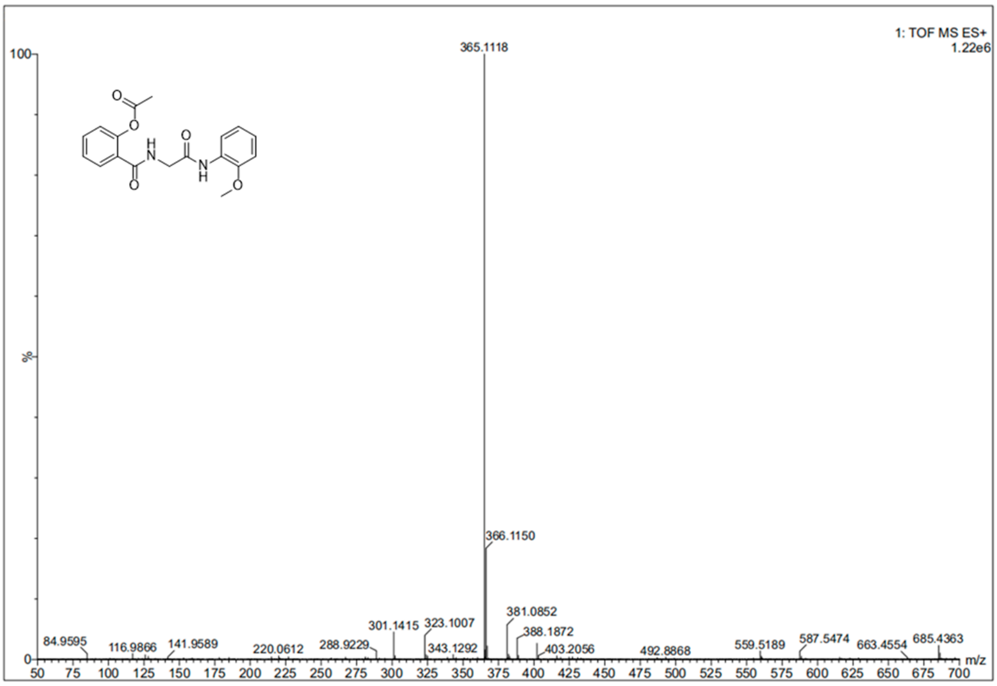

HRMS of compound **3e**

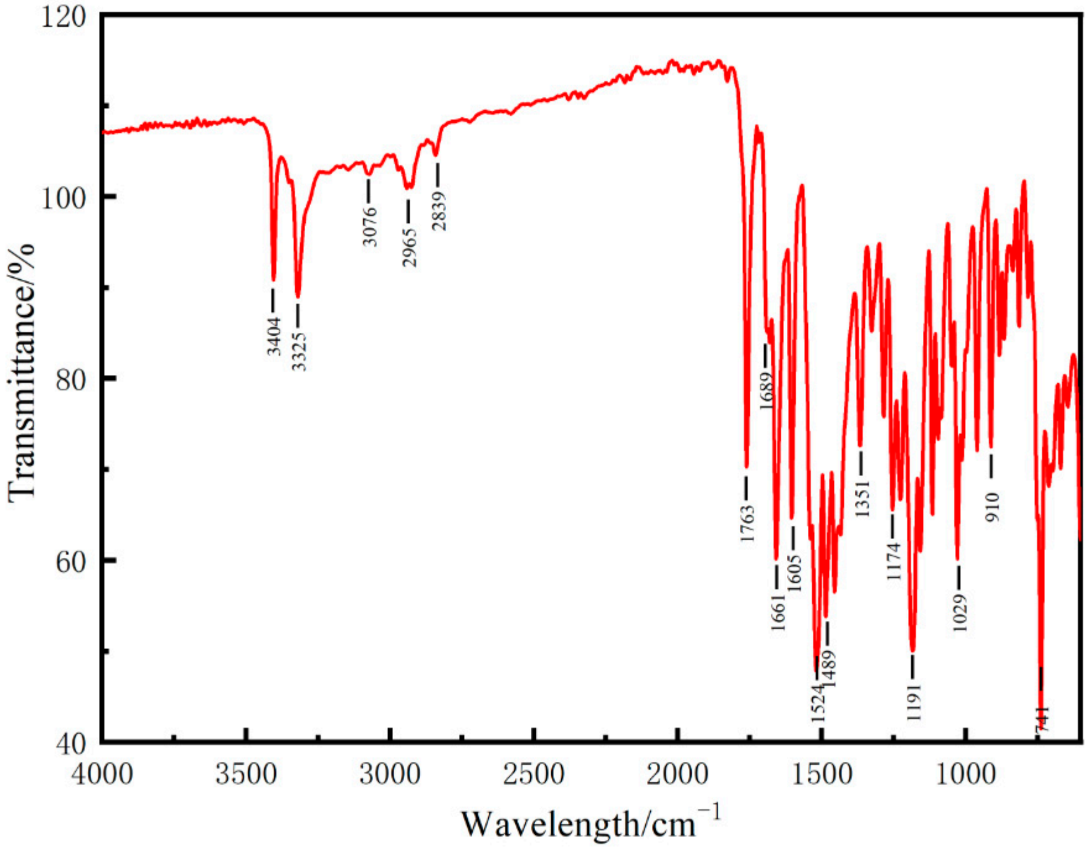

IR of compound **3e**

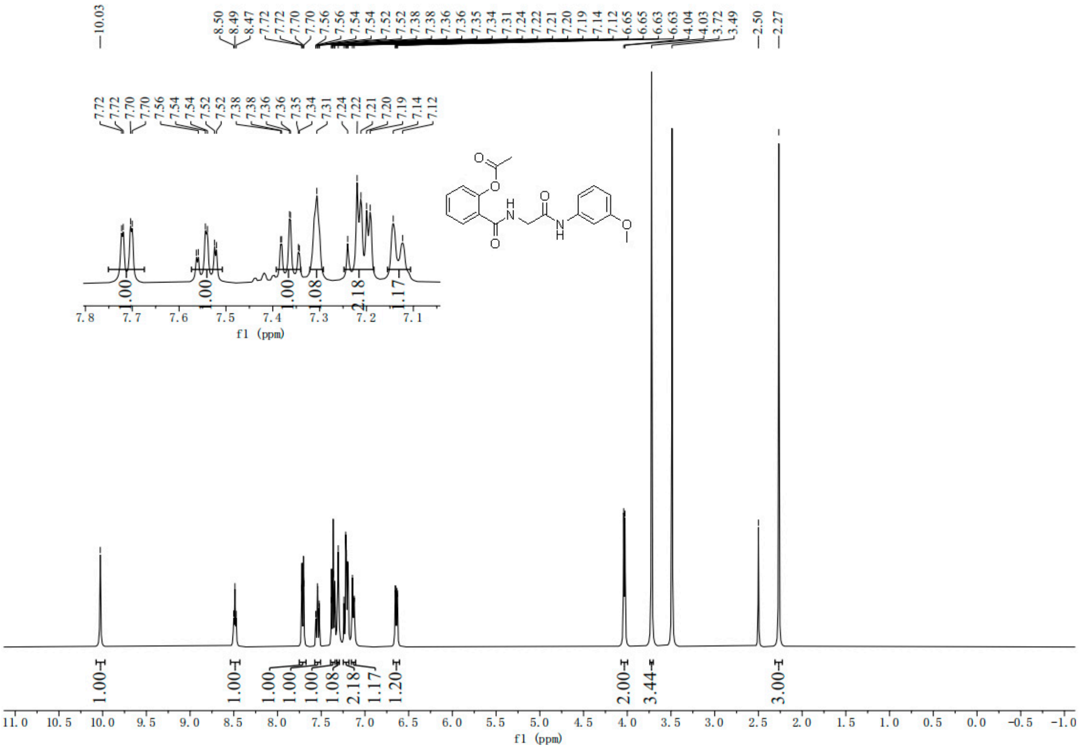

^{1}H NMR of compound **3f** (DMSO-d_6)

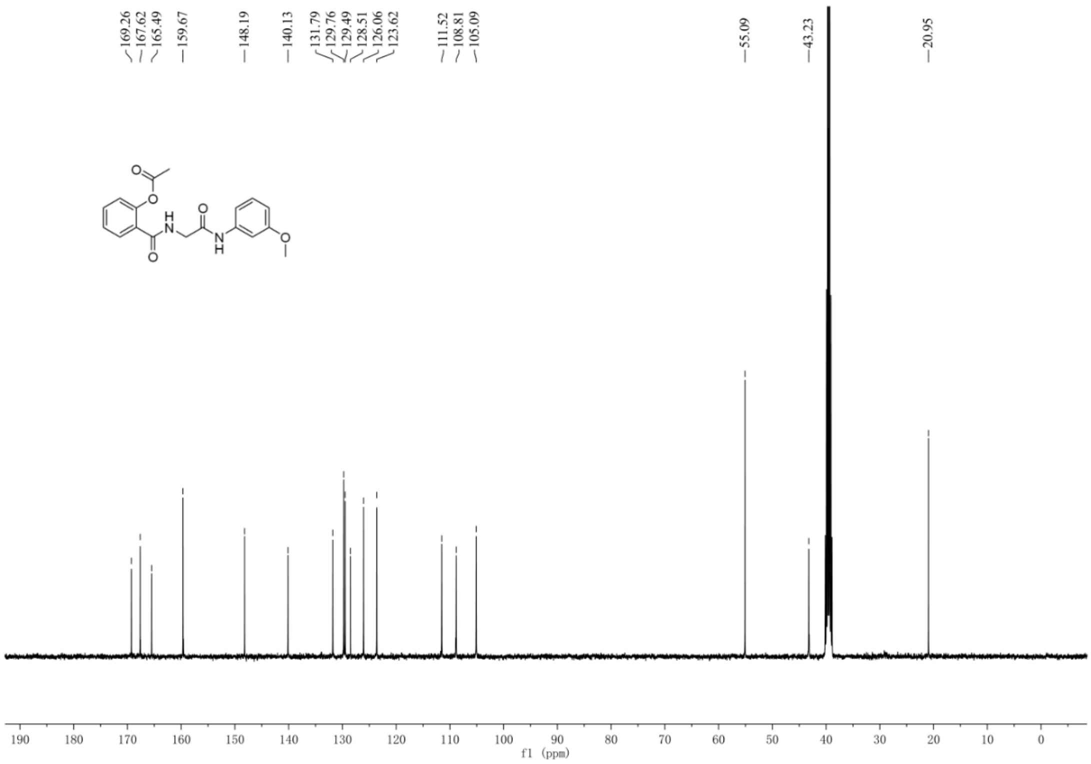

^{13}C NMR of compound **3f** (DMSO-d_6)

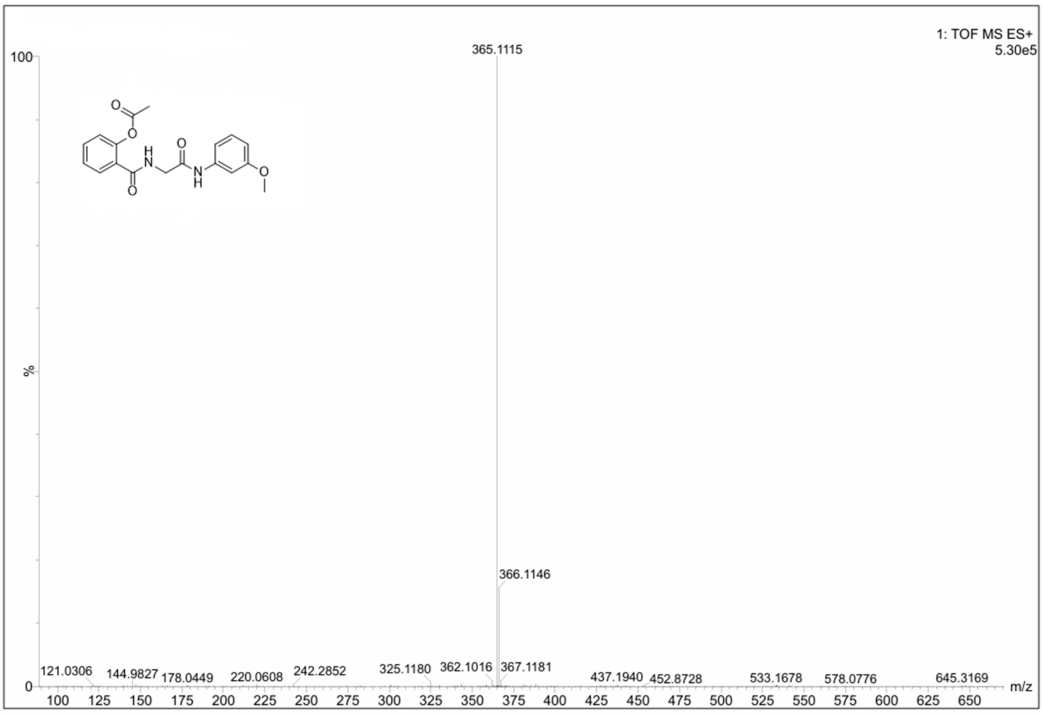

HRMS of compound **3f**

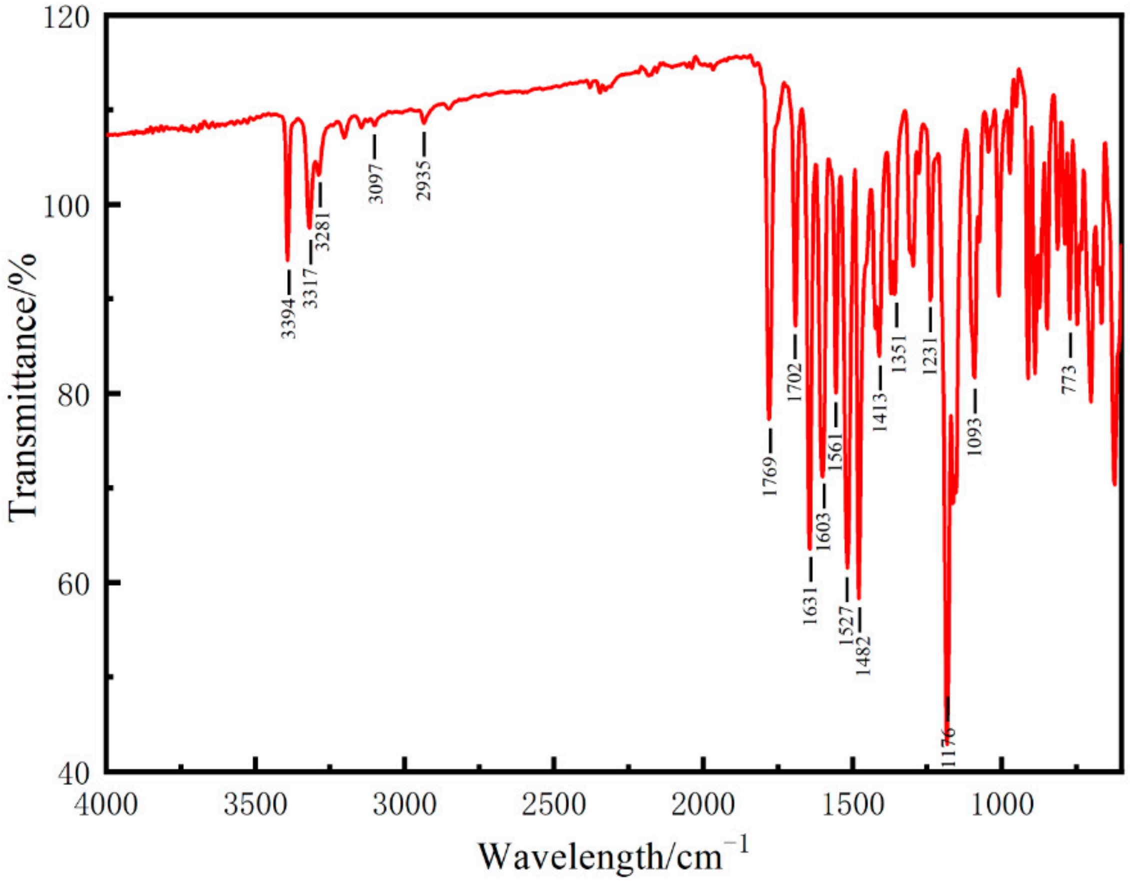

IR of compound **3f**

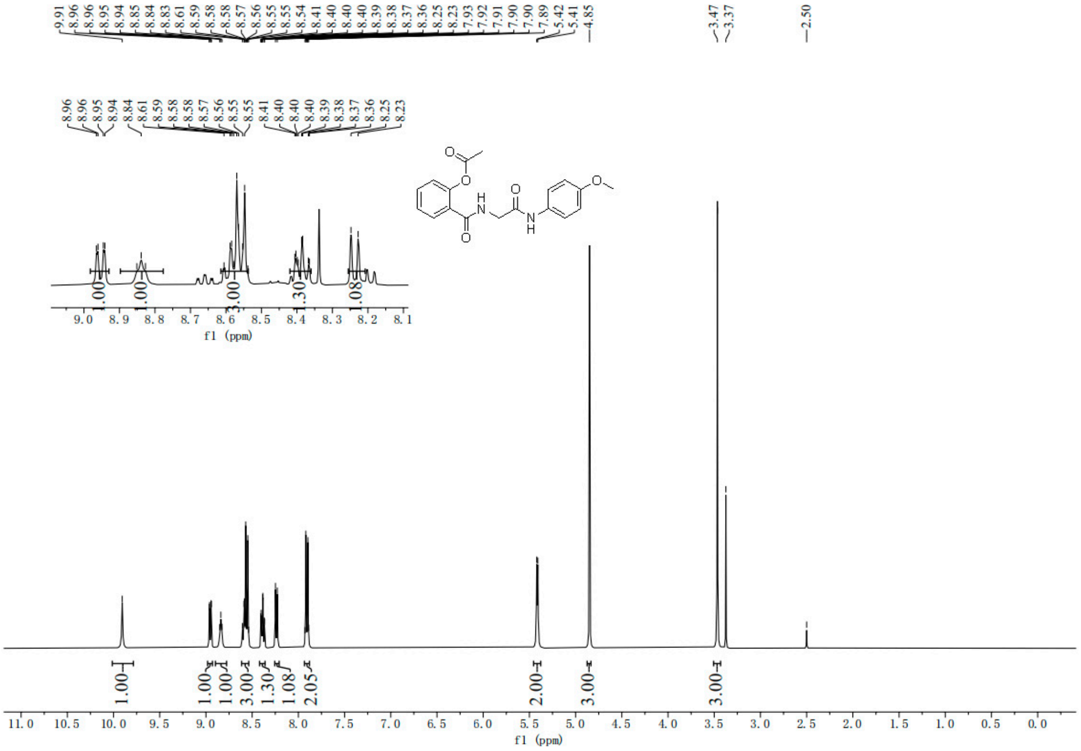

^{1}H NMR of compound **3g** (DMSO-d_6)

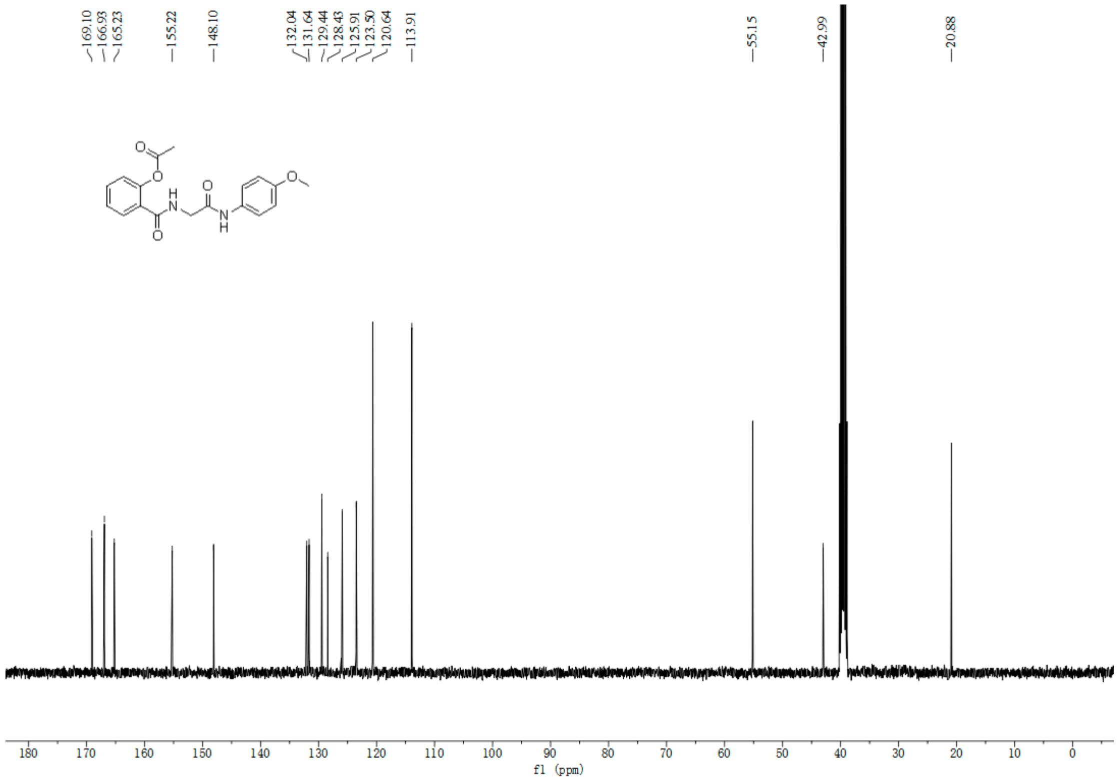

^{13}C NMR of compound **3g** (DMSO-d_6)

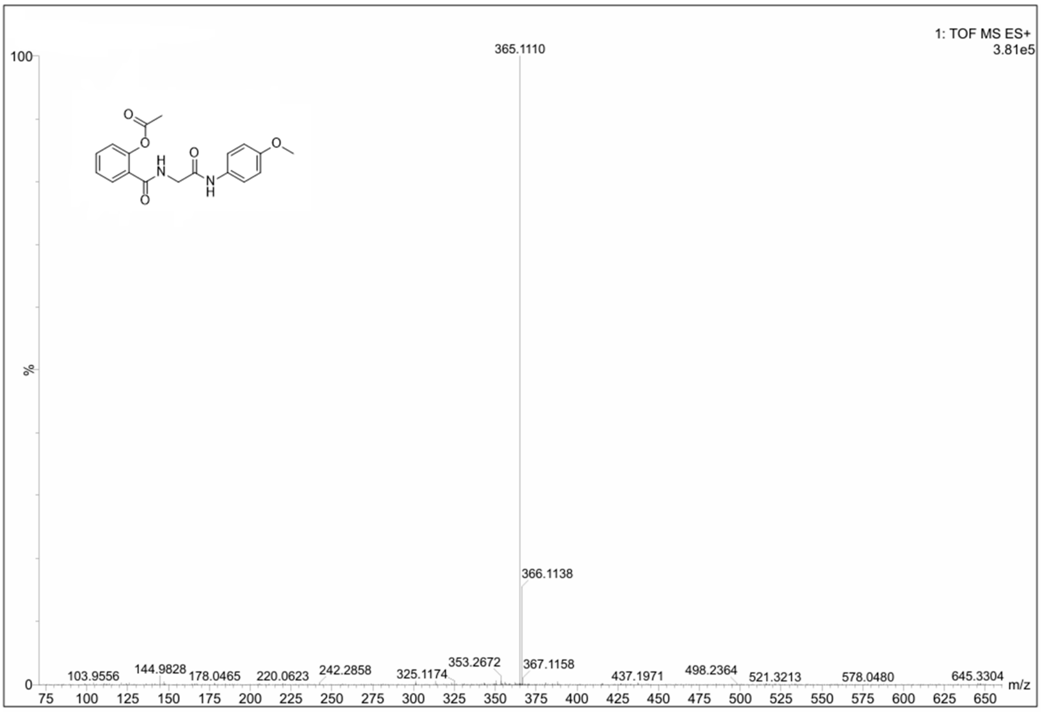

HRMS of compound **3g**

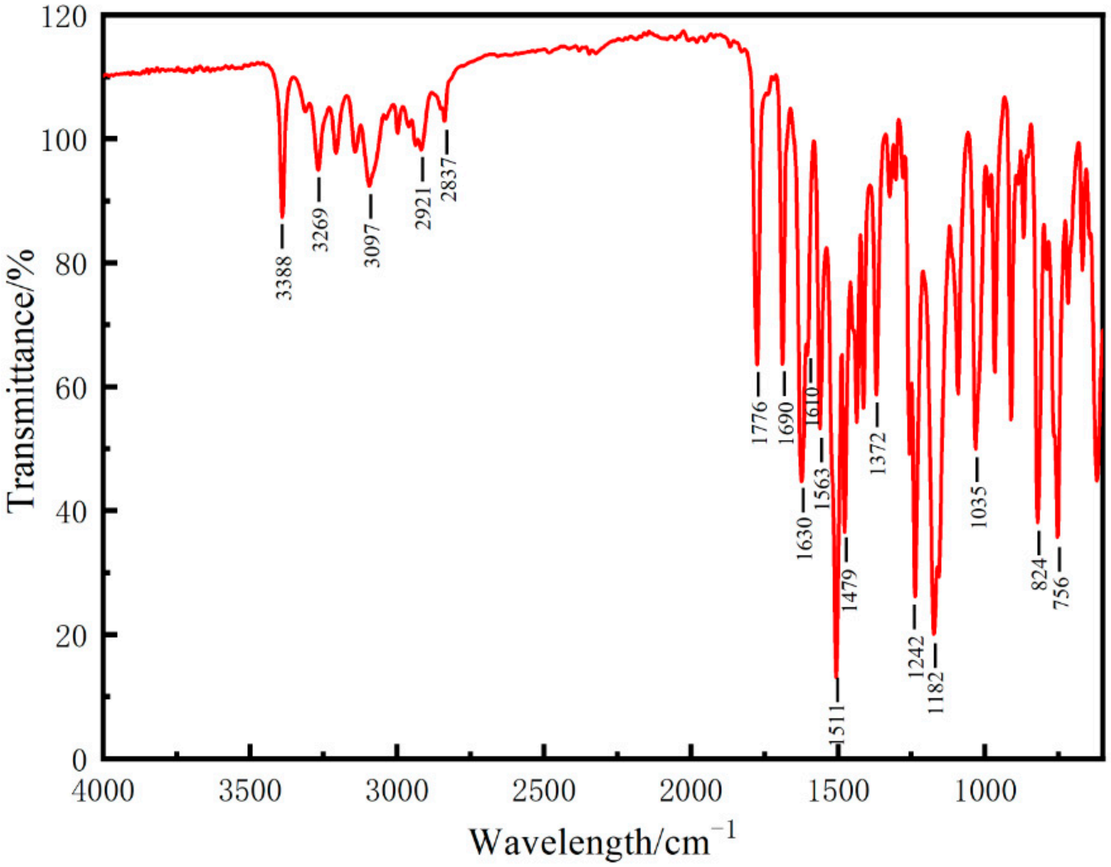

IR of compound **3g**

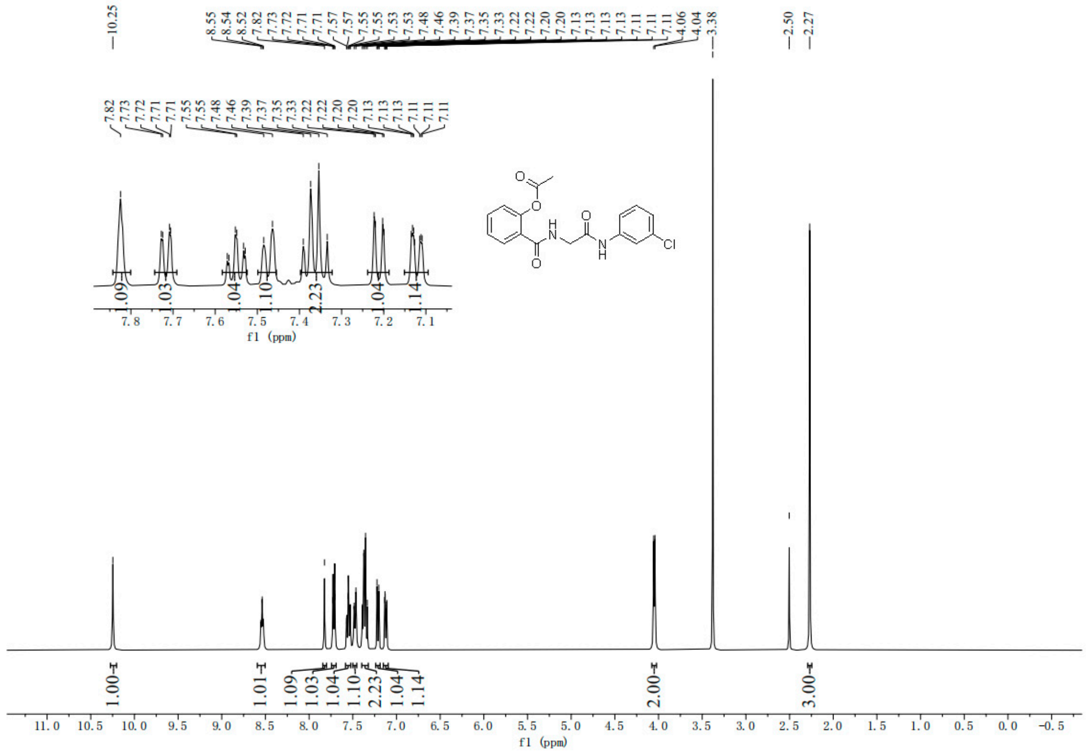

^{1}H NMR of compound **3h** (DMSO-d_6)

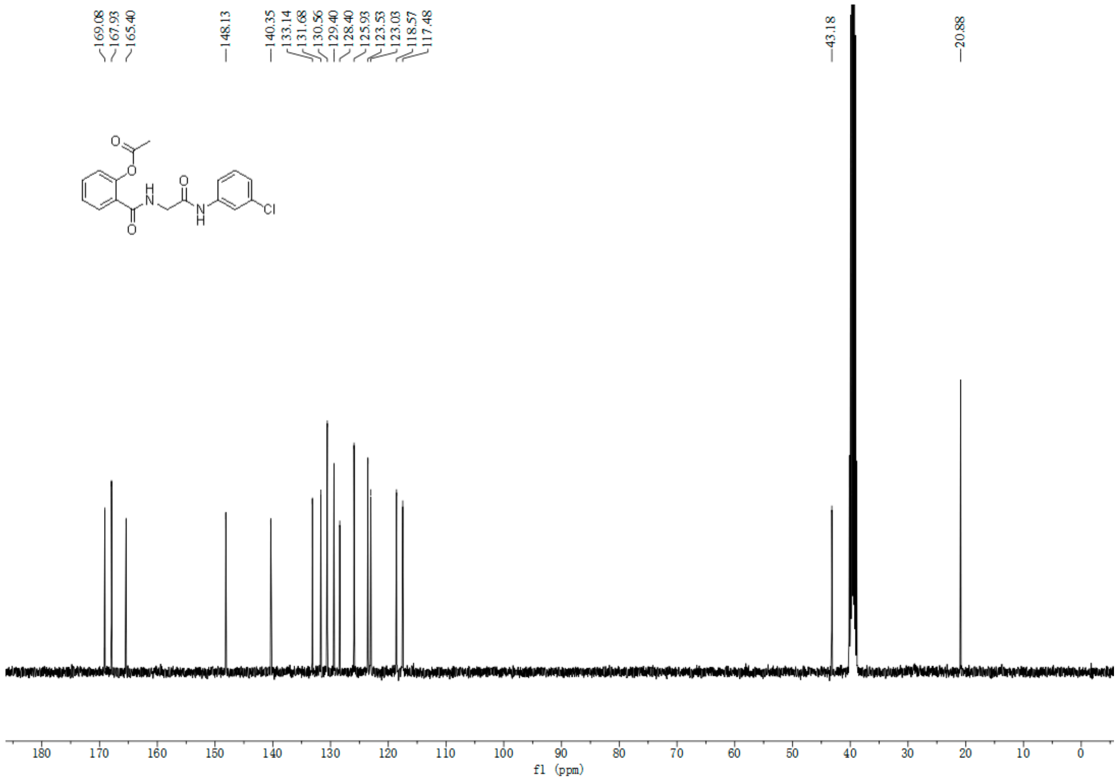

^{13}C NMR of compound **3h** (DMSO-d_6)

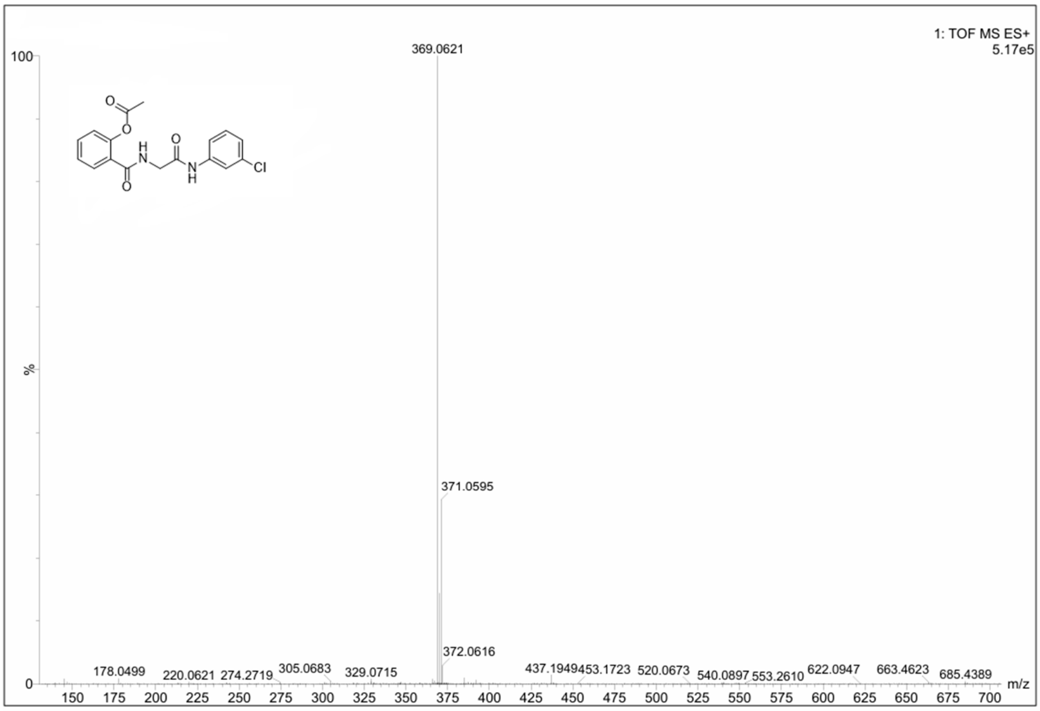

HRMS of compound **3h**

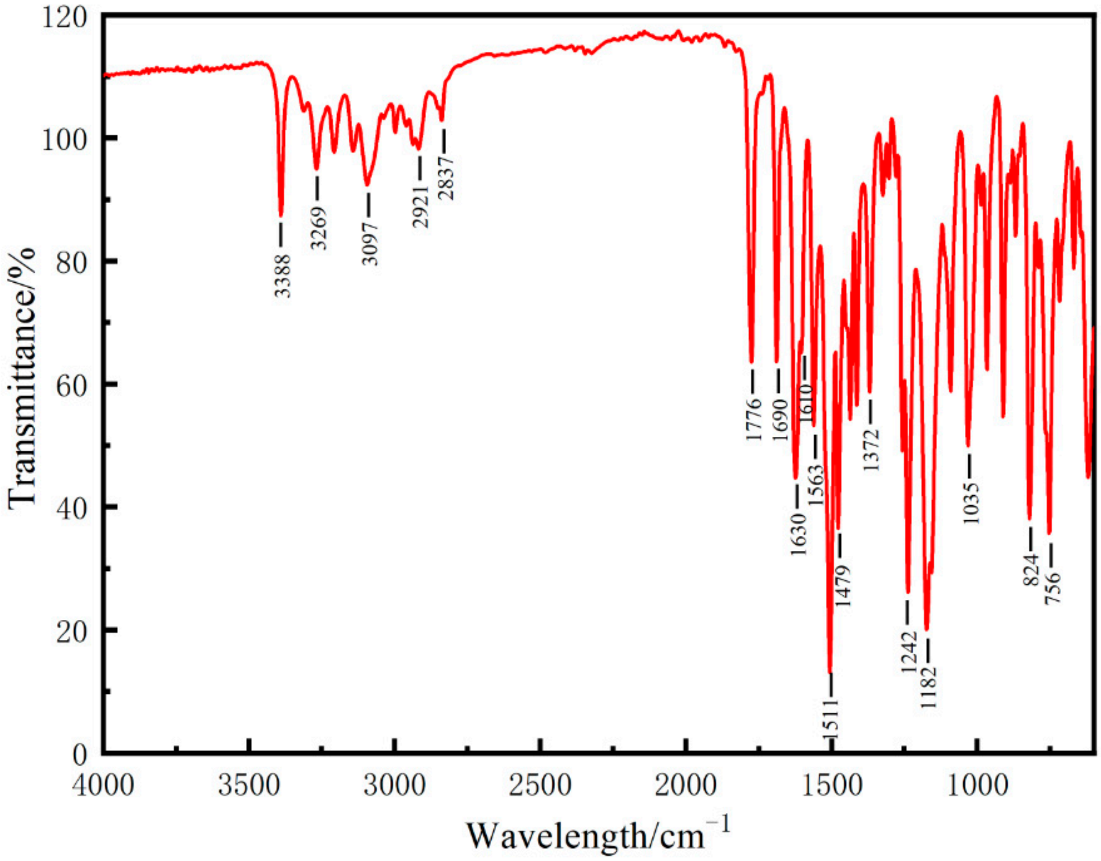

IR of compound **3h**

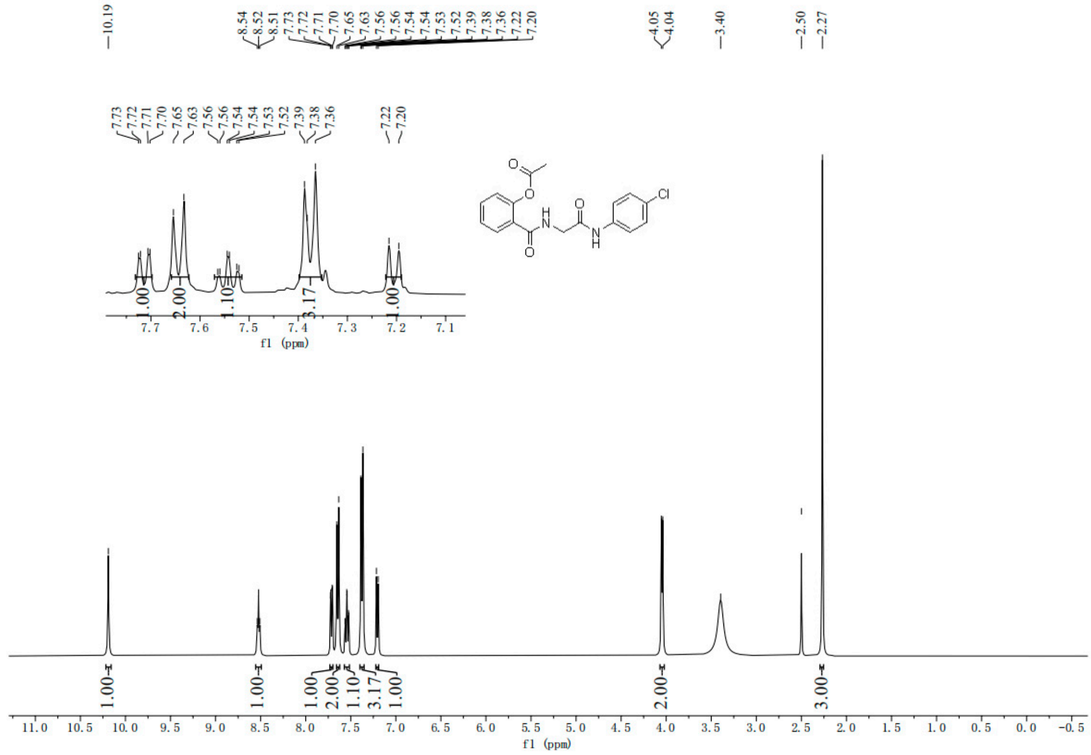

^{1}H NMR of compound **3i** (DMSO-d_6)

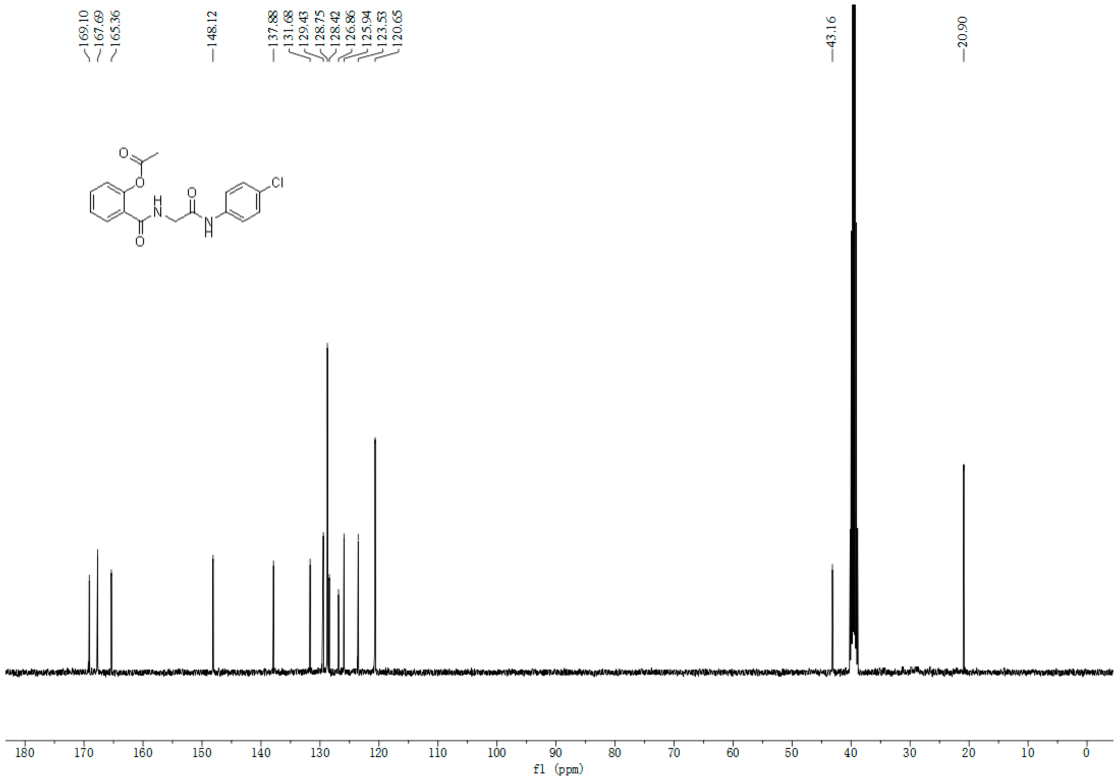

^{13}C NMR of compound **3i** (DMSO-d_6)

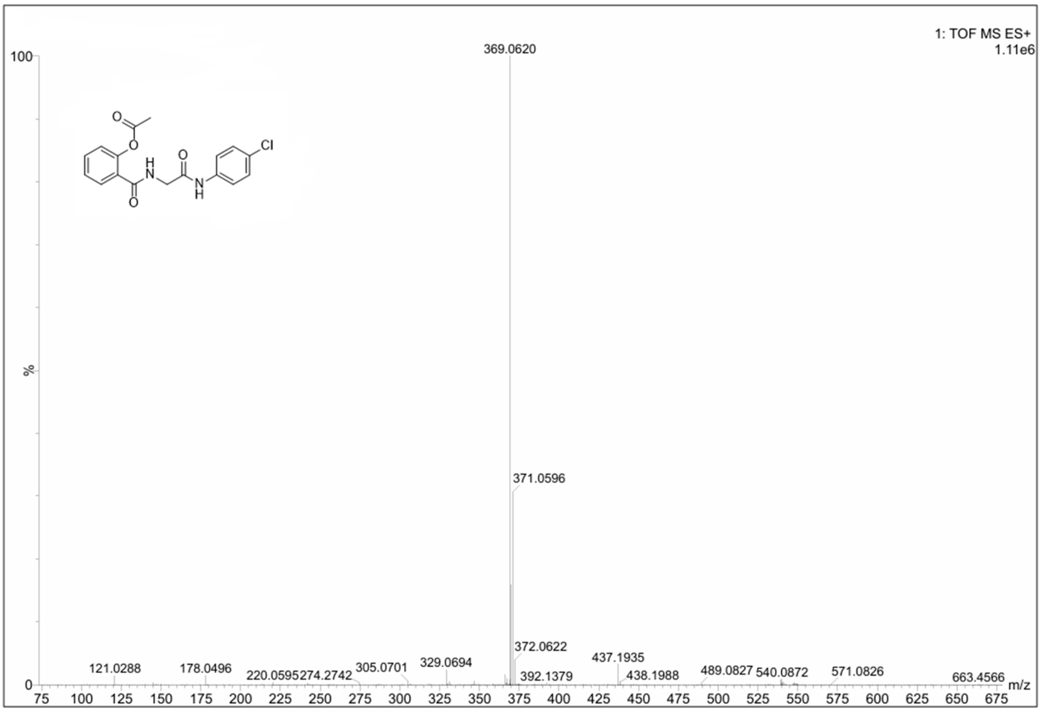

HRMS of compound **3i**

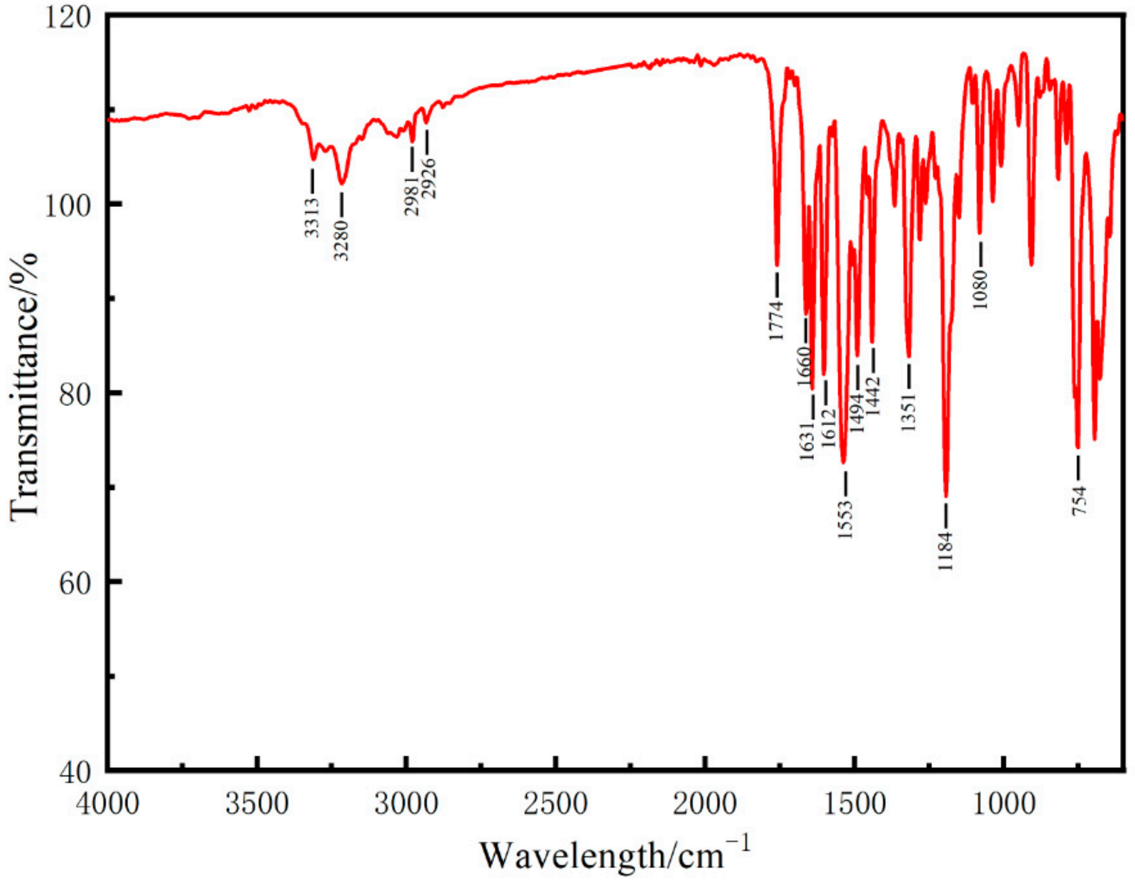

IR of compound **3i**

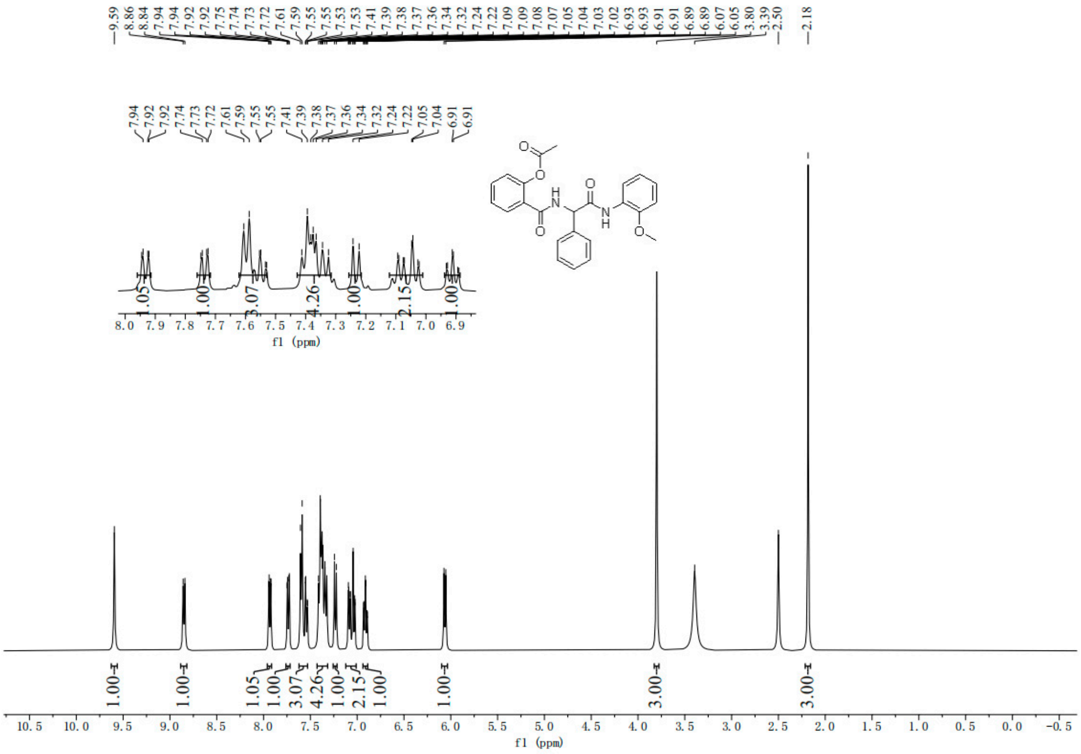

^{1}H NMR of compound **3j** (DMSO-d_6)

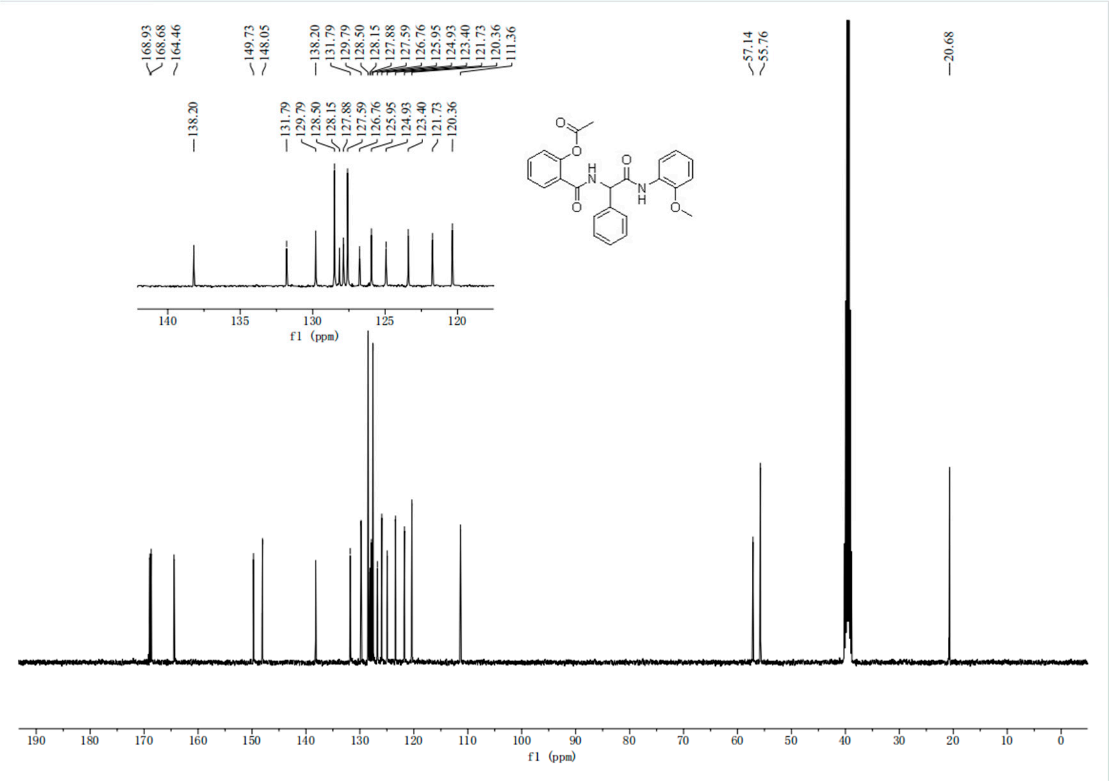

^{13}C NMR of compound **3j** (DMSO-d_6)

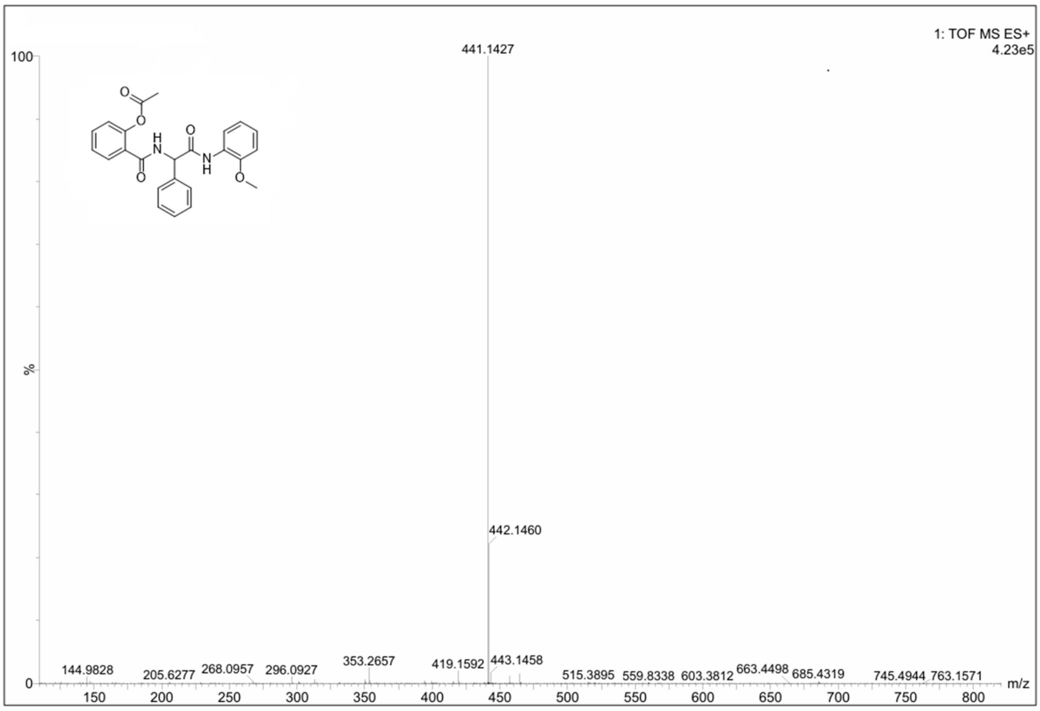

HRMS of compound **3j**

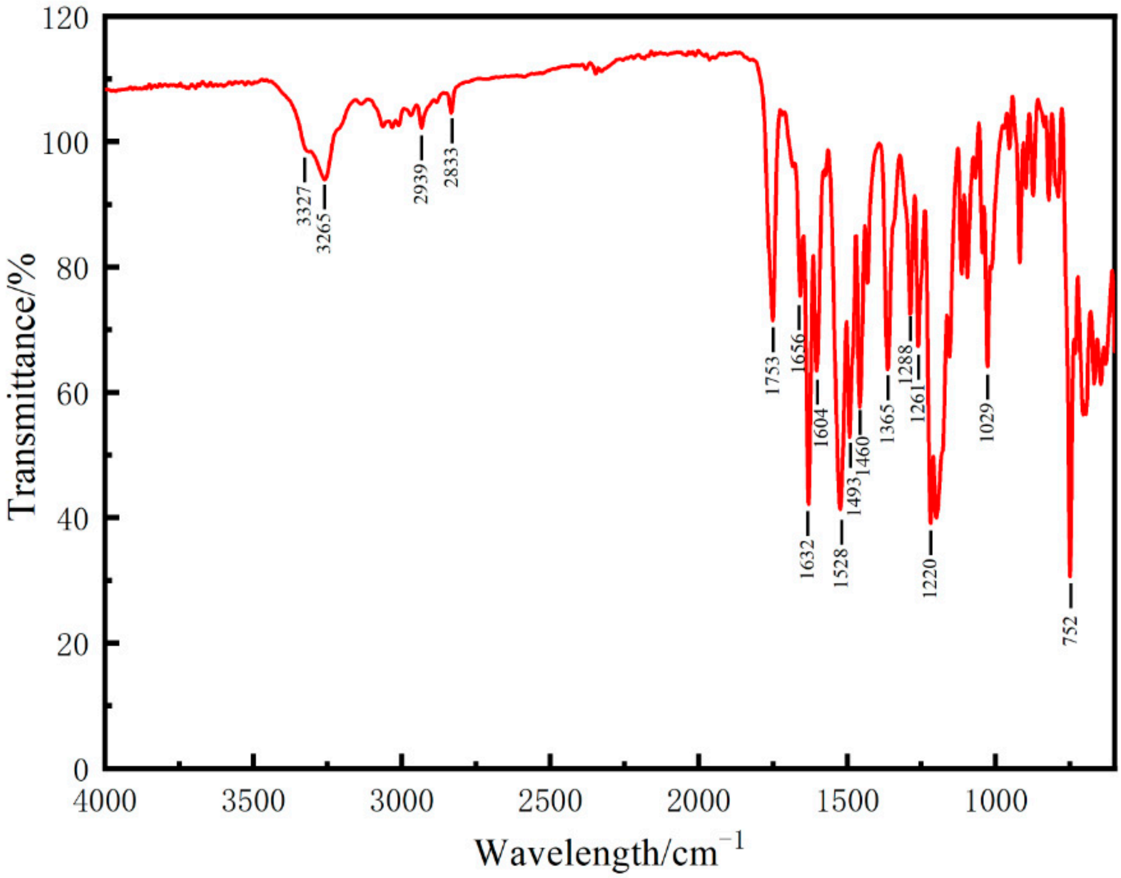

IR of compound **3j**

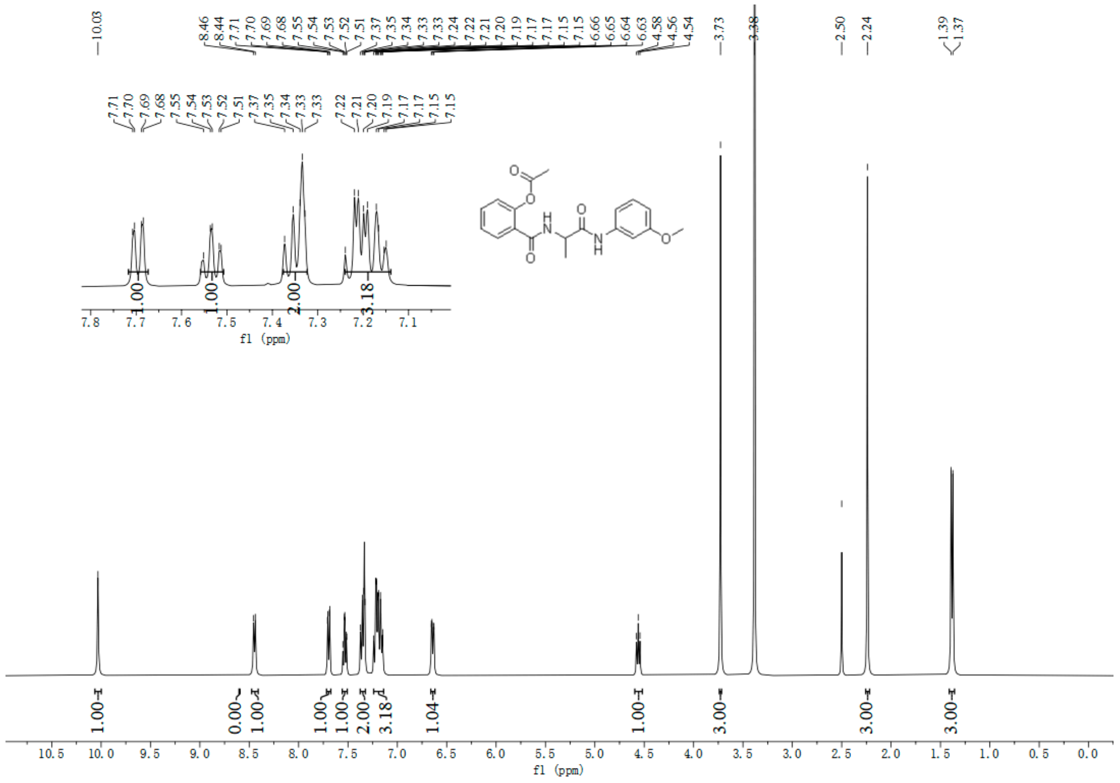

^{1}H NMR of compound **3k** (DMSO-d_6)

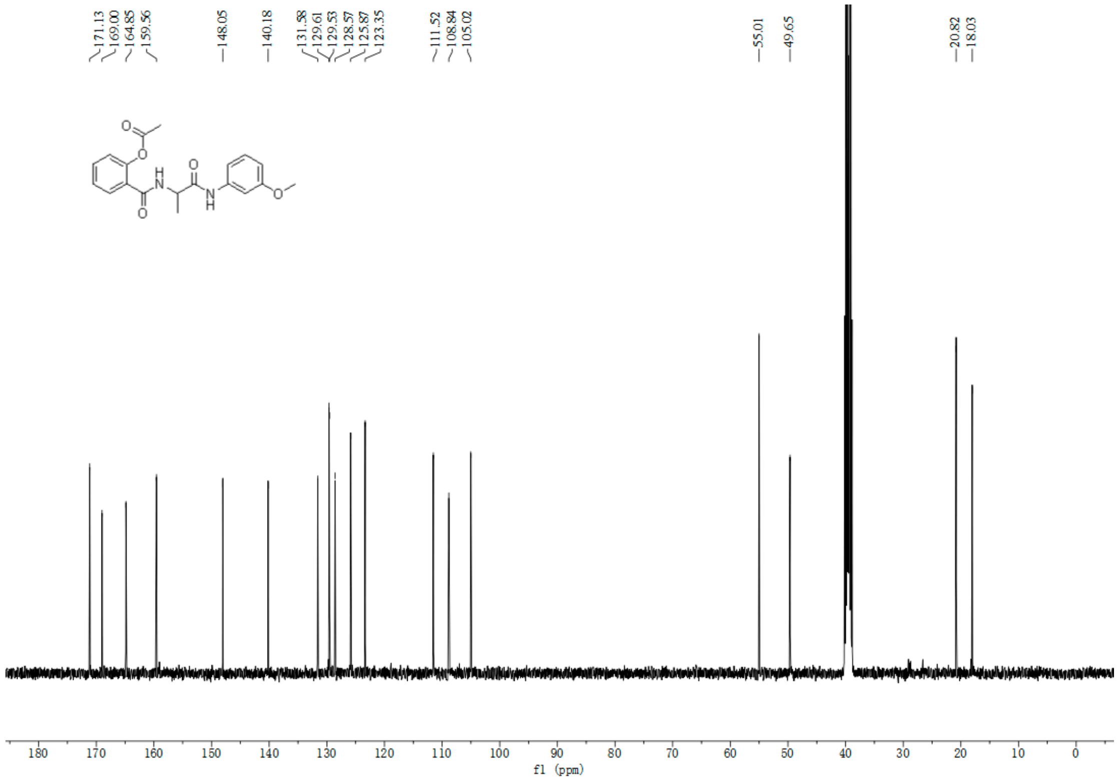

^{13}C NMR of compound **3k** (DMSO-d_6)

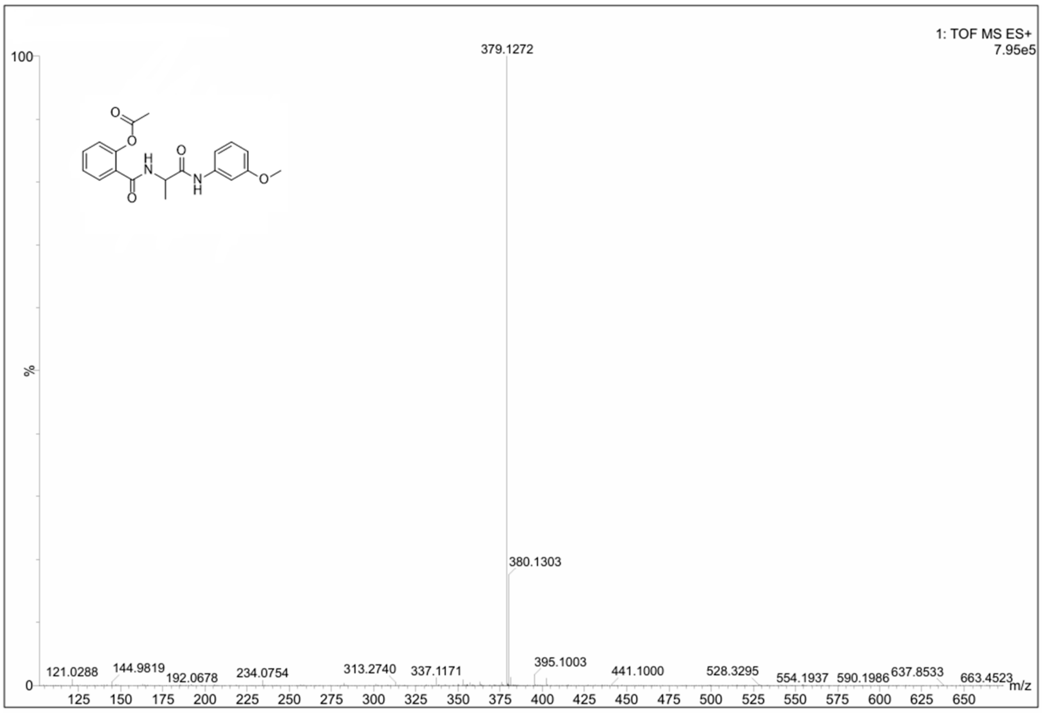

HRMS of compound **3k**

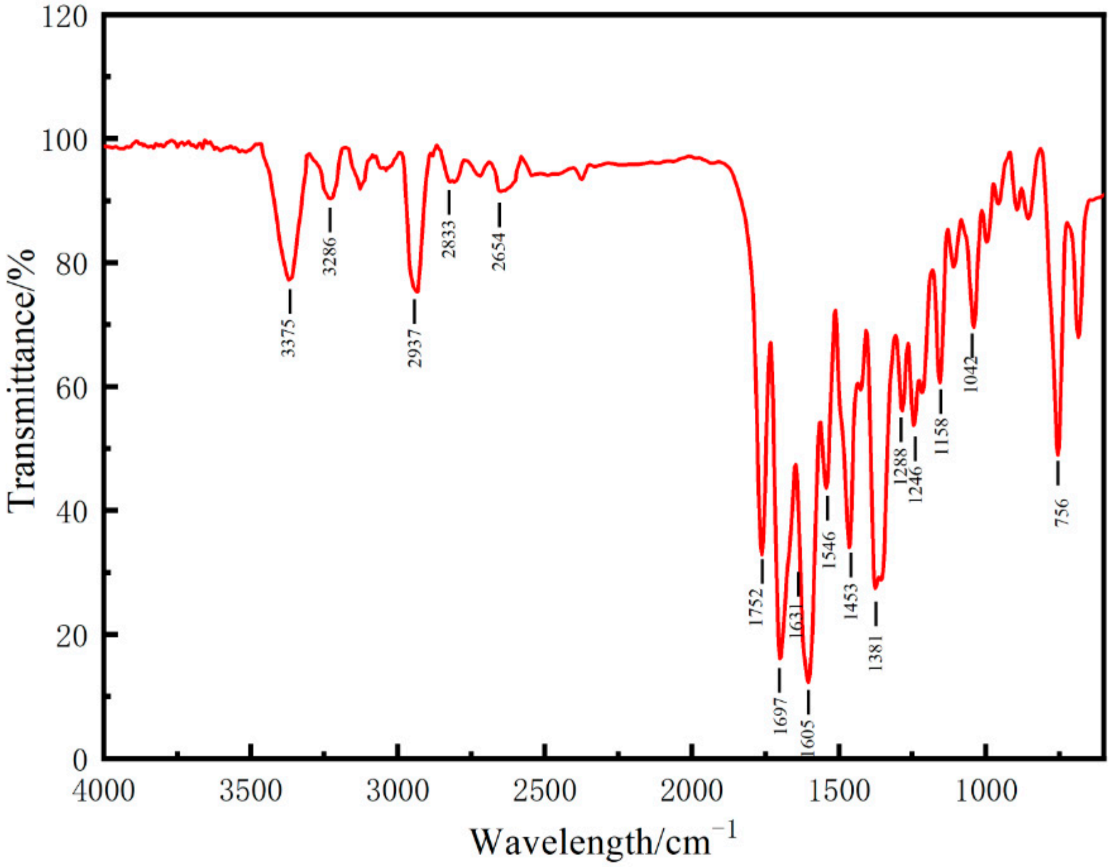

IR of compound **3k**

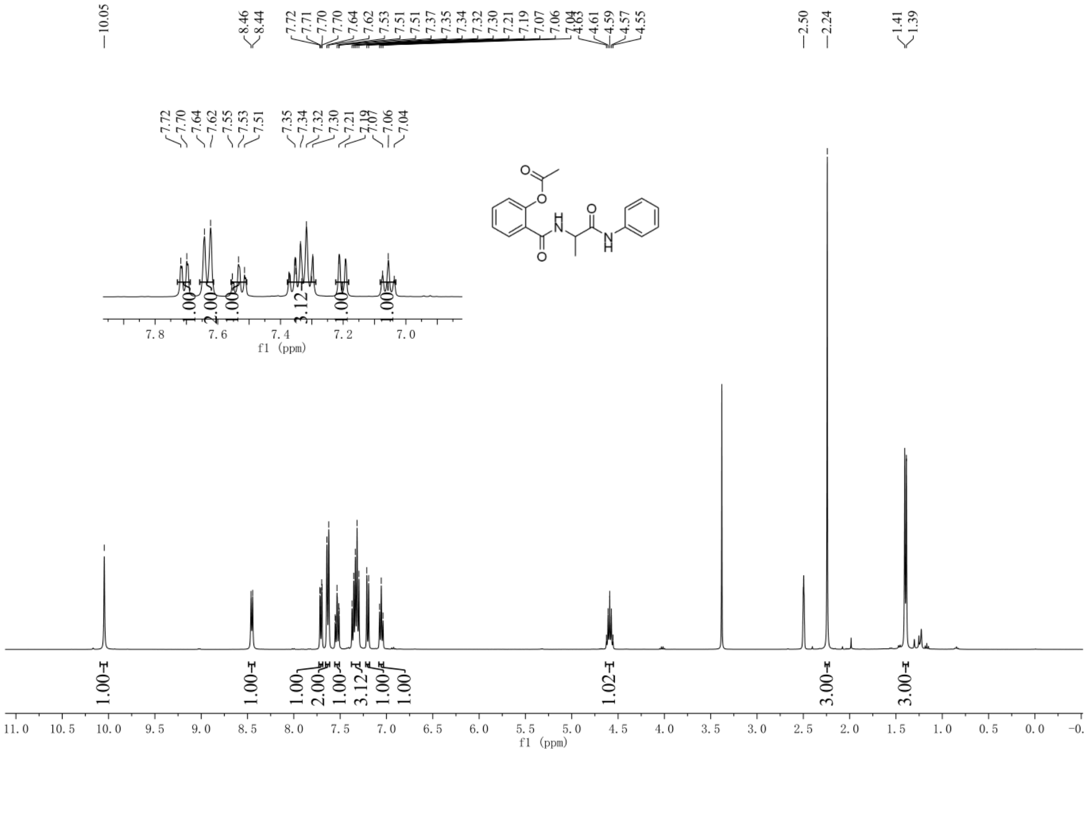

^{1}H NMR of compound **3l** (DMSO-d_6)

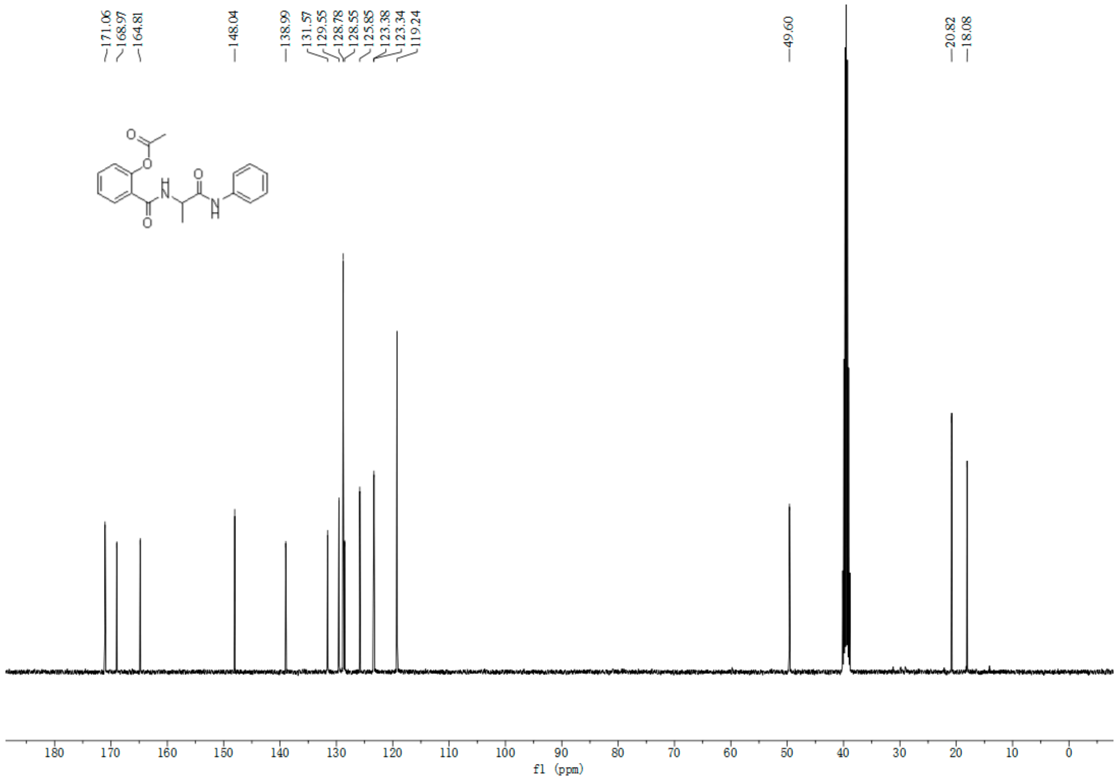

^{13}C NMR of compound **3l** (DMSO-d_6)

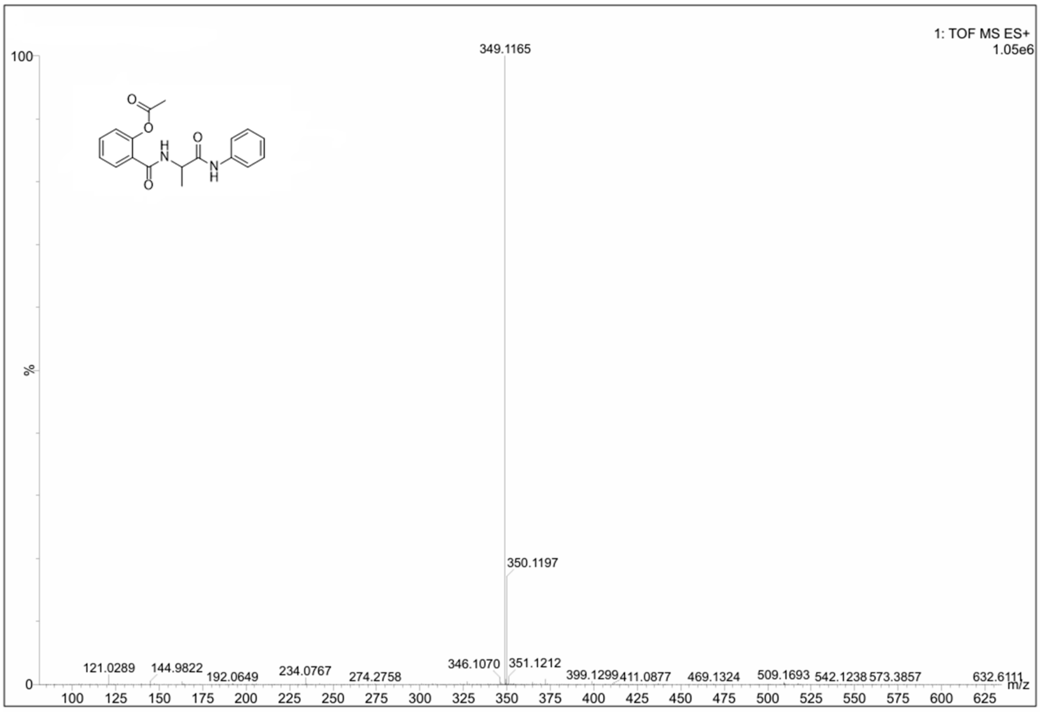

HRMS of compound **3l**

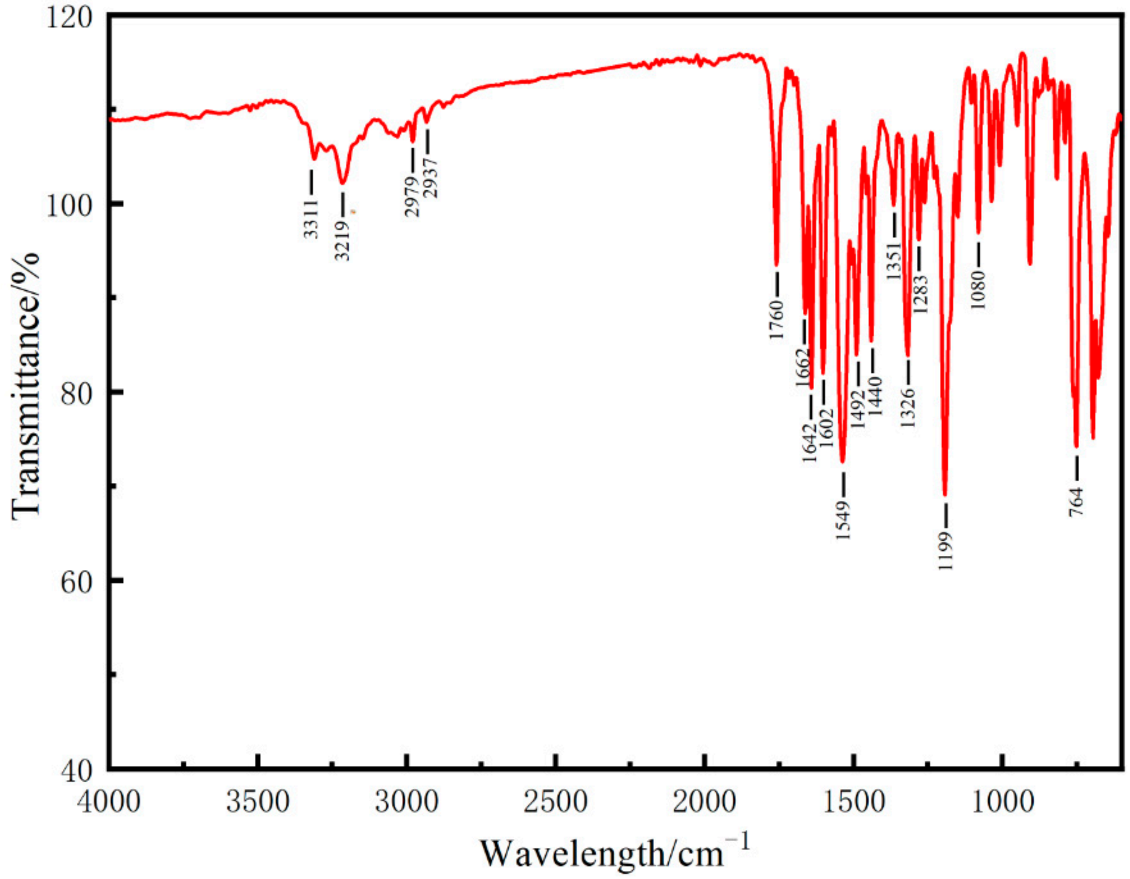

IR of compound **3l**

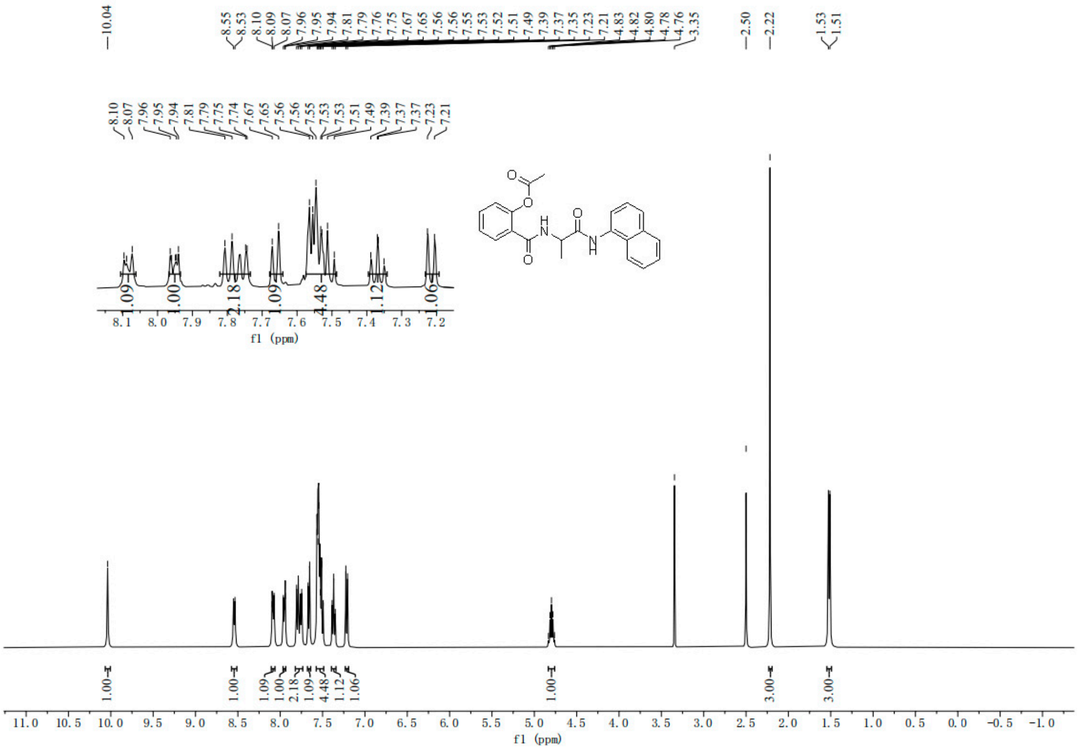

^{1}H NMR of compound **3m** (DMSO-d_6)

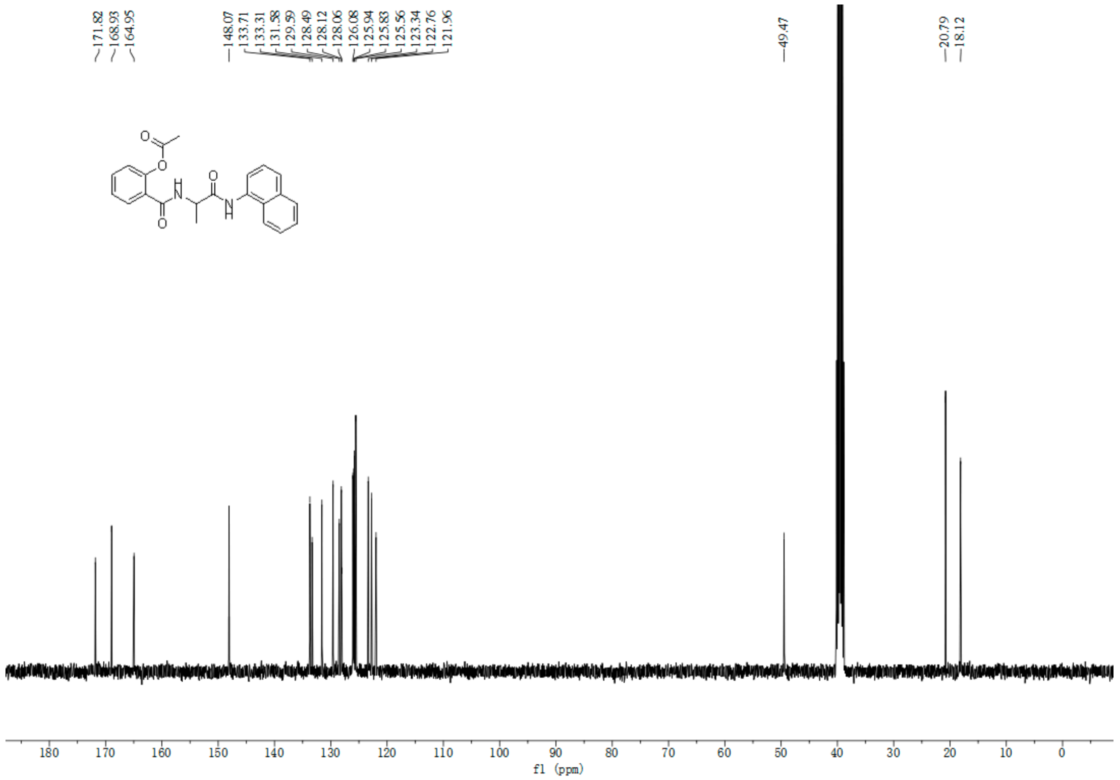

^{13}C NMR of compound **3m** (DMSO-d_6)

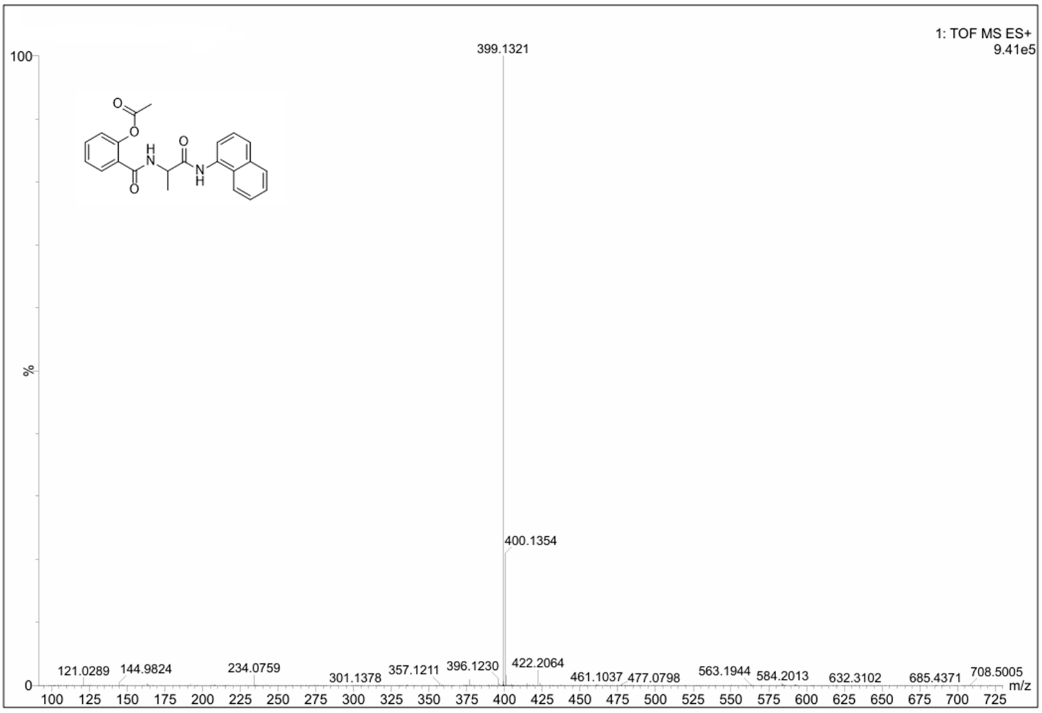

HRMS of compound **3m**

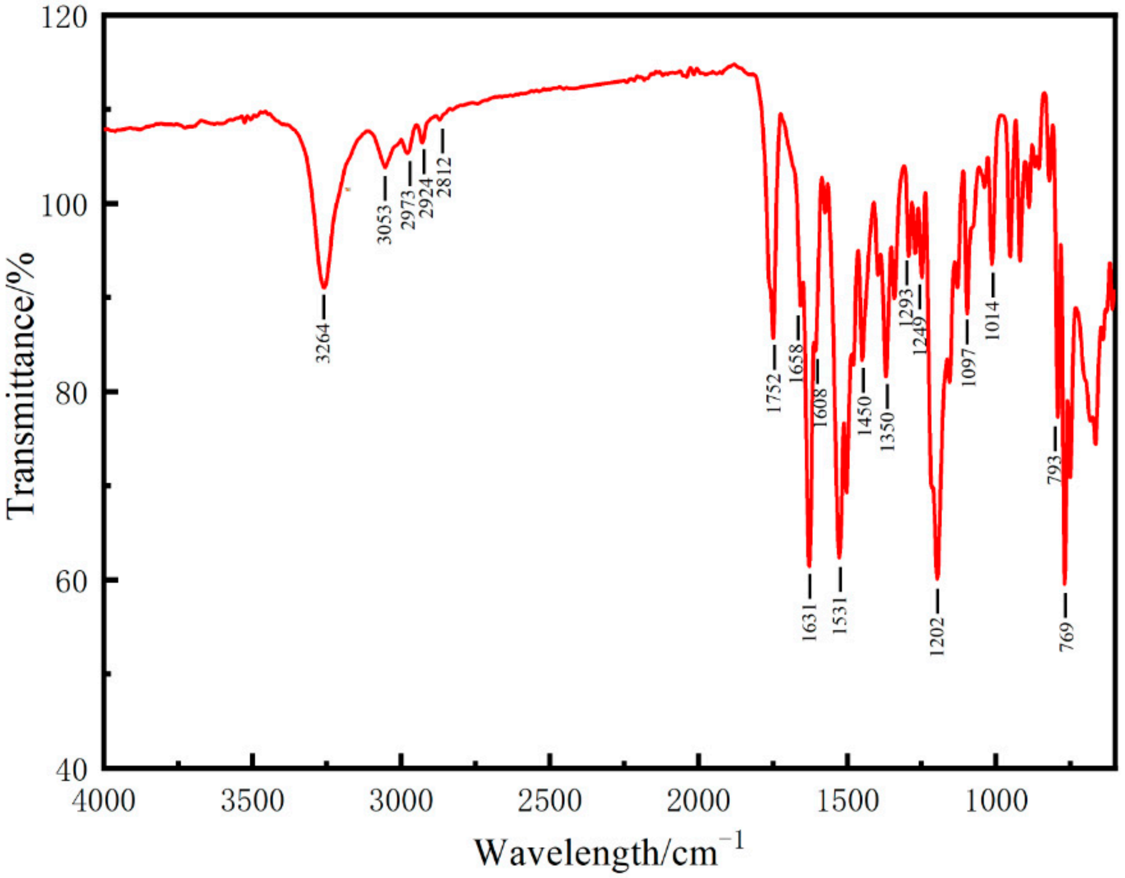

IR of compound **3m**

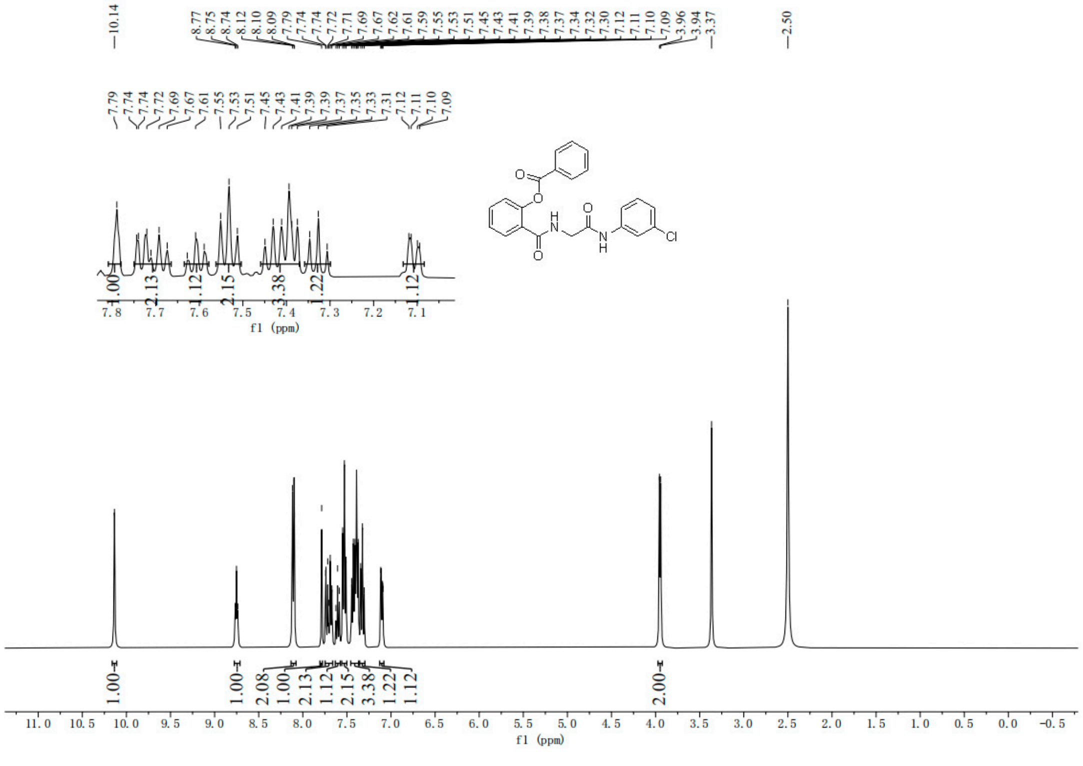

^{1}H NMR of compound **5a** (DMSO-d_6)

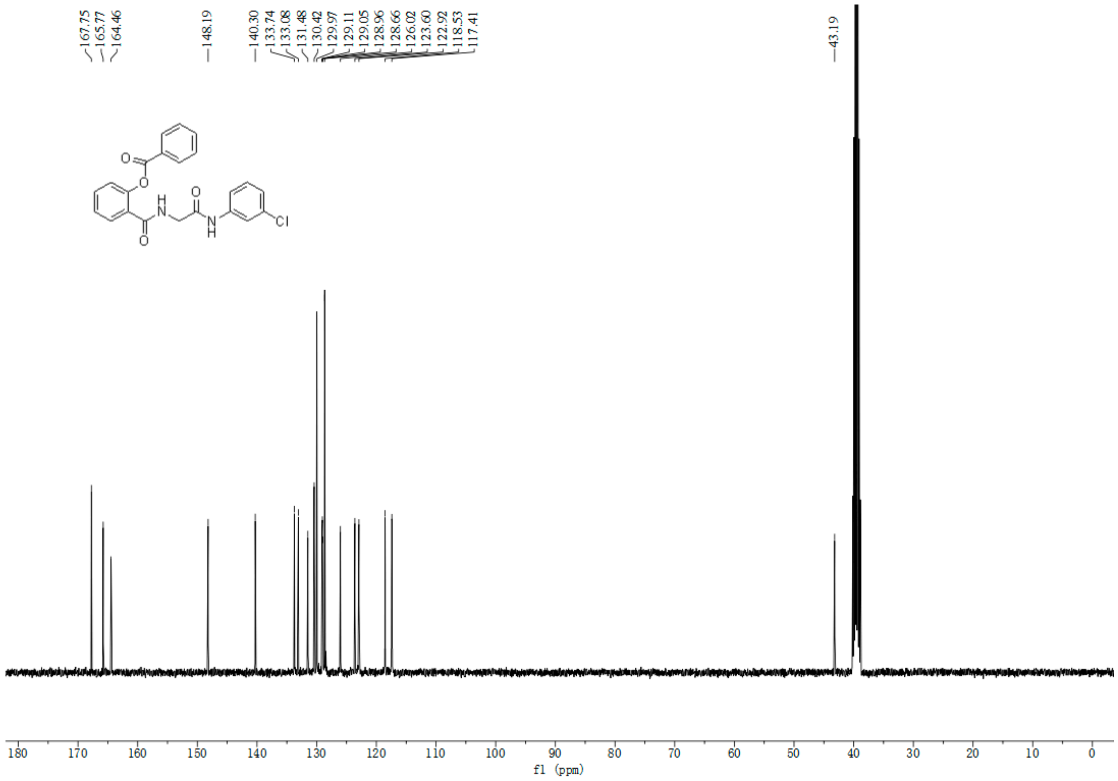

^{13}C NMR of compound **5a** (DMSO-d_6)

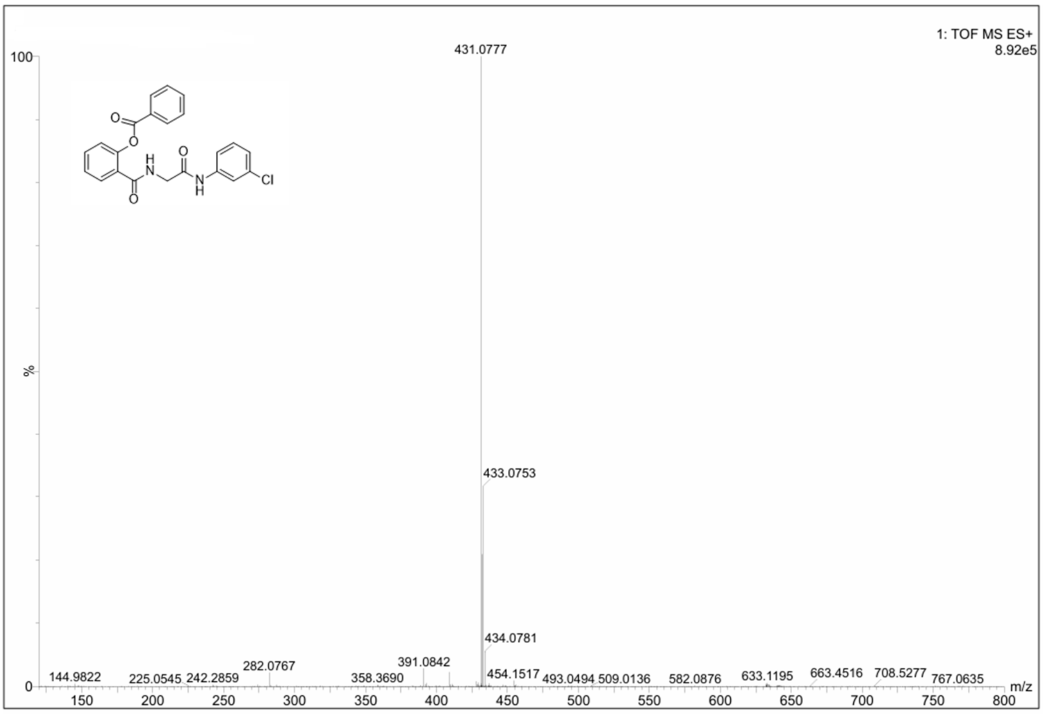

HRMS of compound **5a**

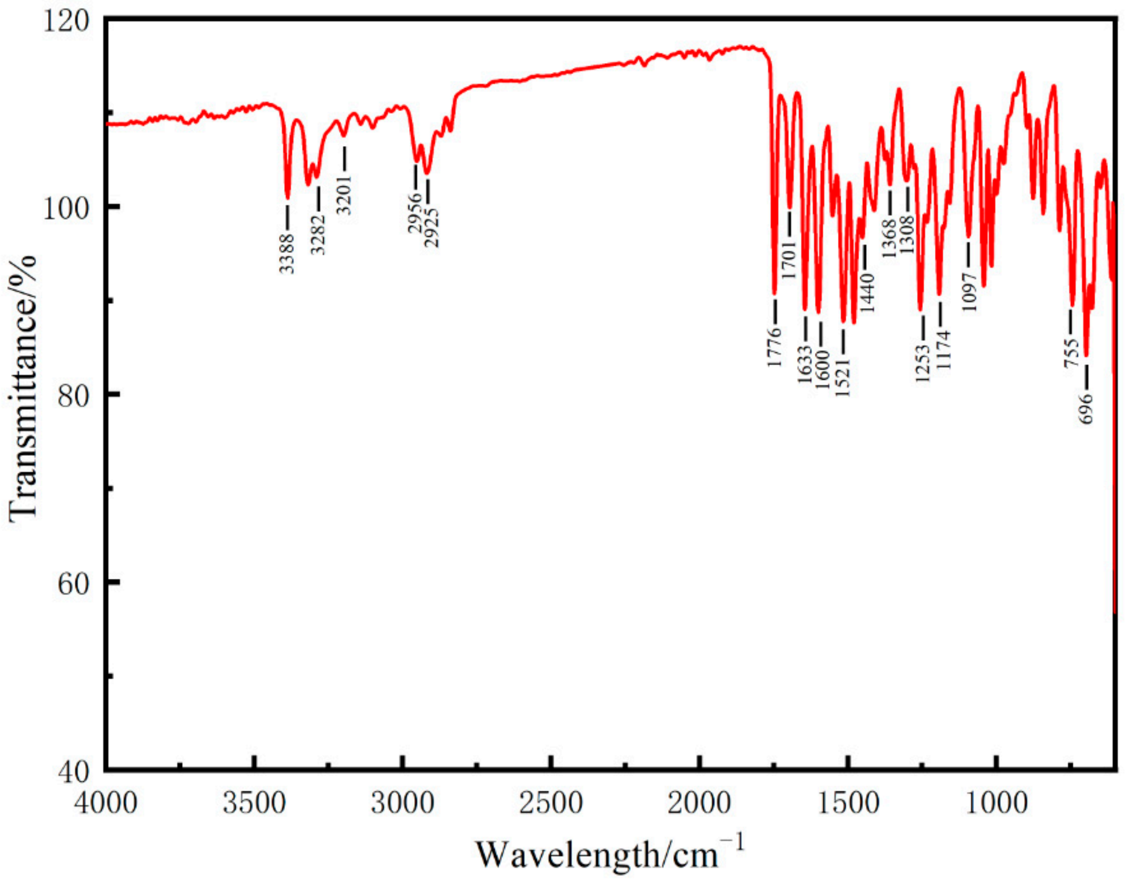

IR of compound **5a**

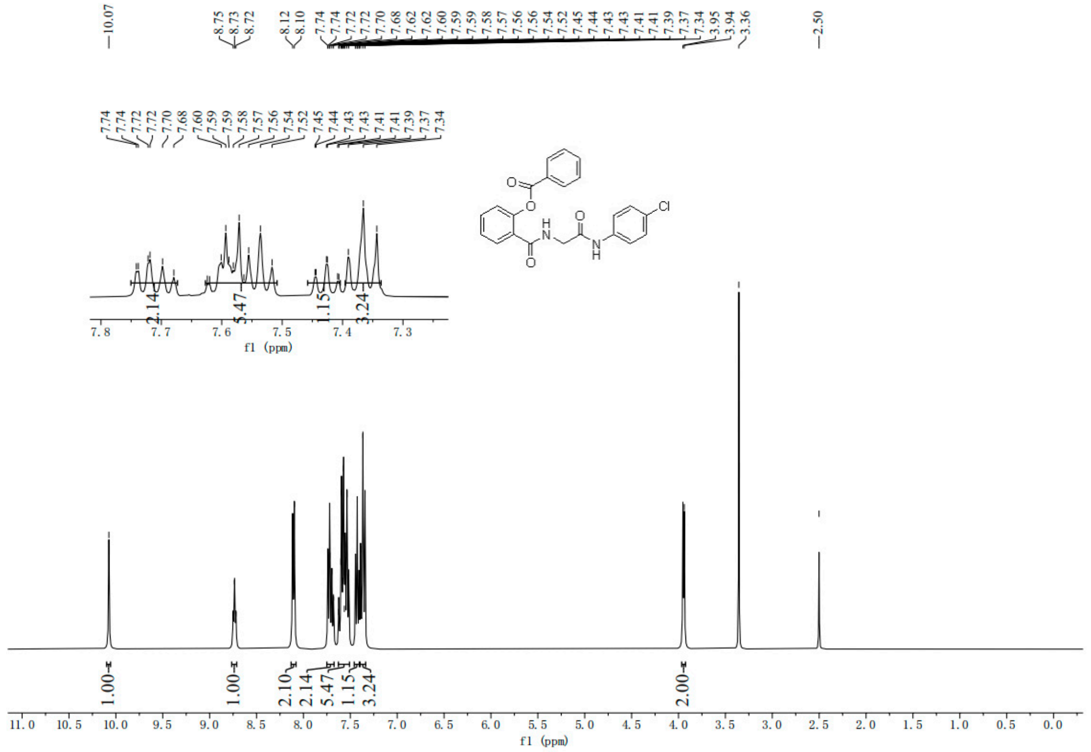

^{1}H NMR of compound **5b** (DMSO-d_6)

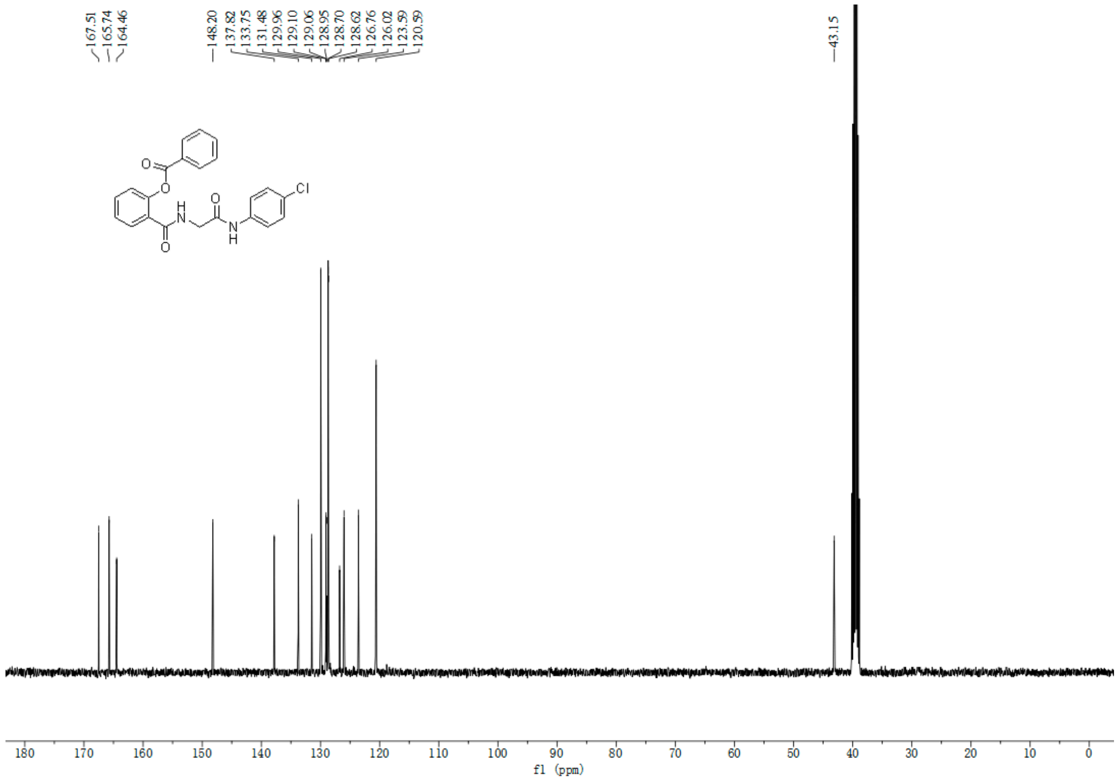

^{13}C NMR of compound **5b** (DMSO-d_6)

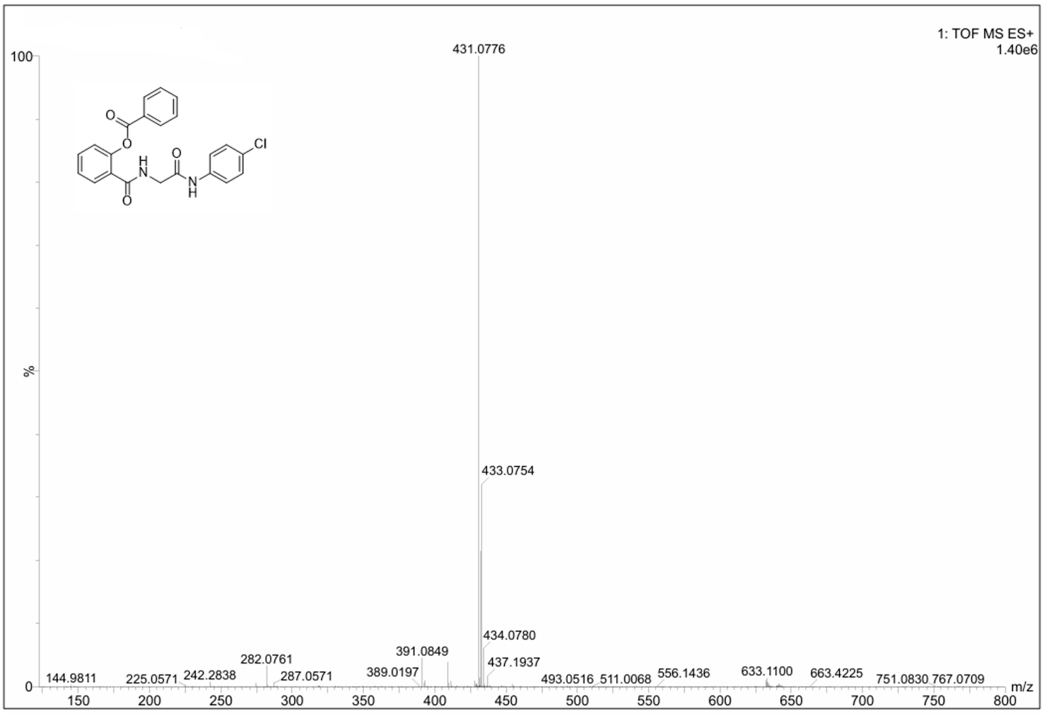

HRMS of compound **5b**

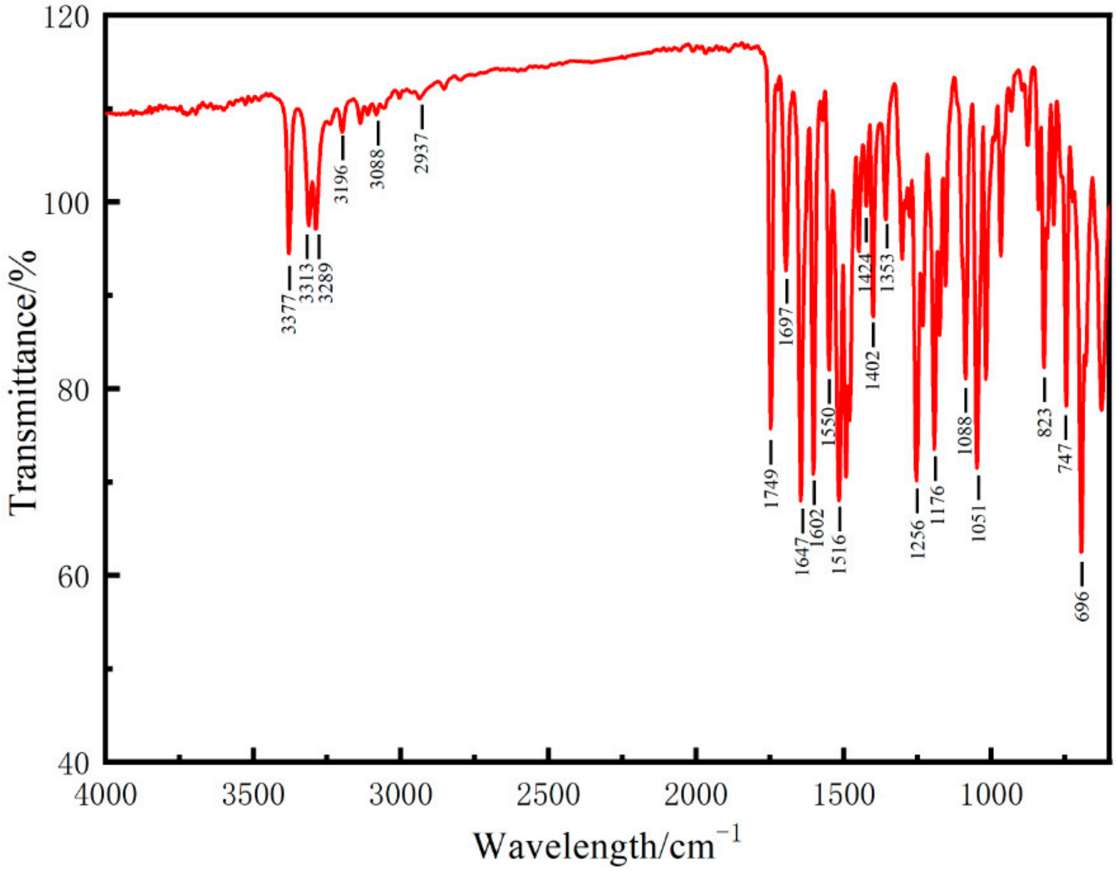

IR of compound **5b**

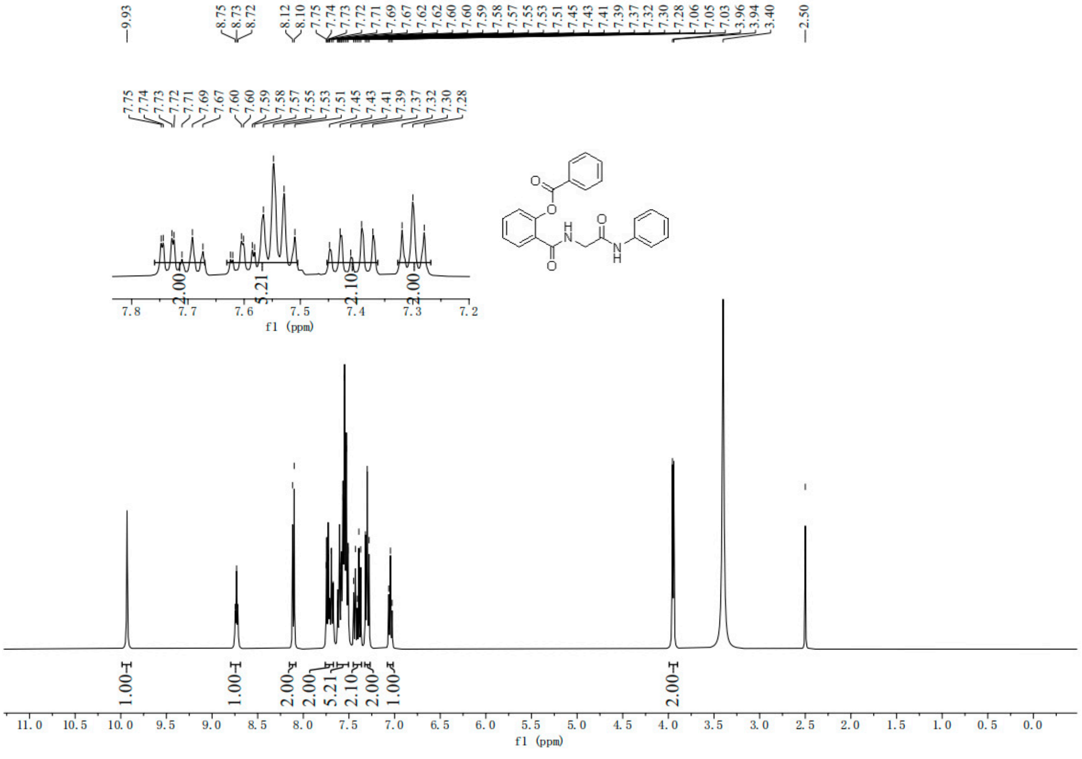

^{1}H NMR of compound **5c** (DMSO-d_6)

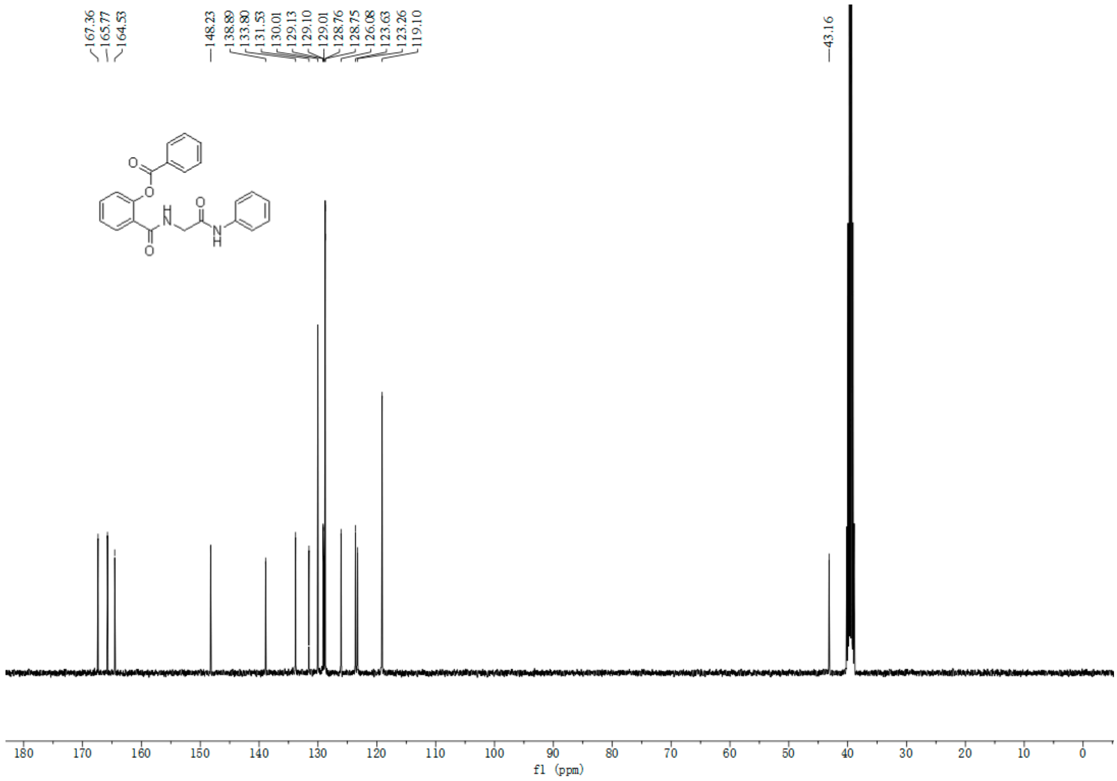

^{13}C NMR of compound **5c** (DMSO-d_6)

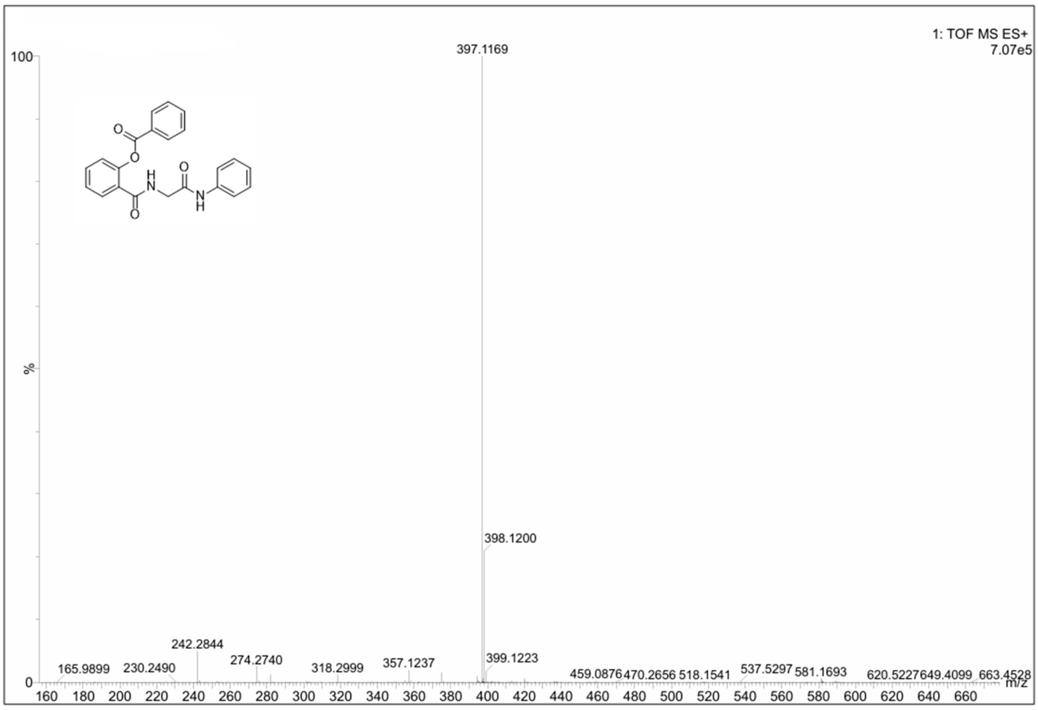

HRMS of compound **5c**

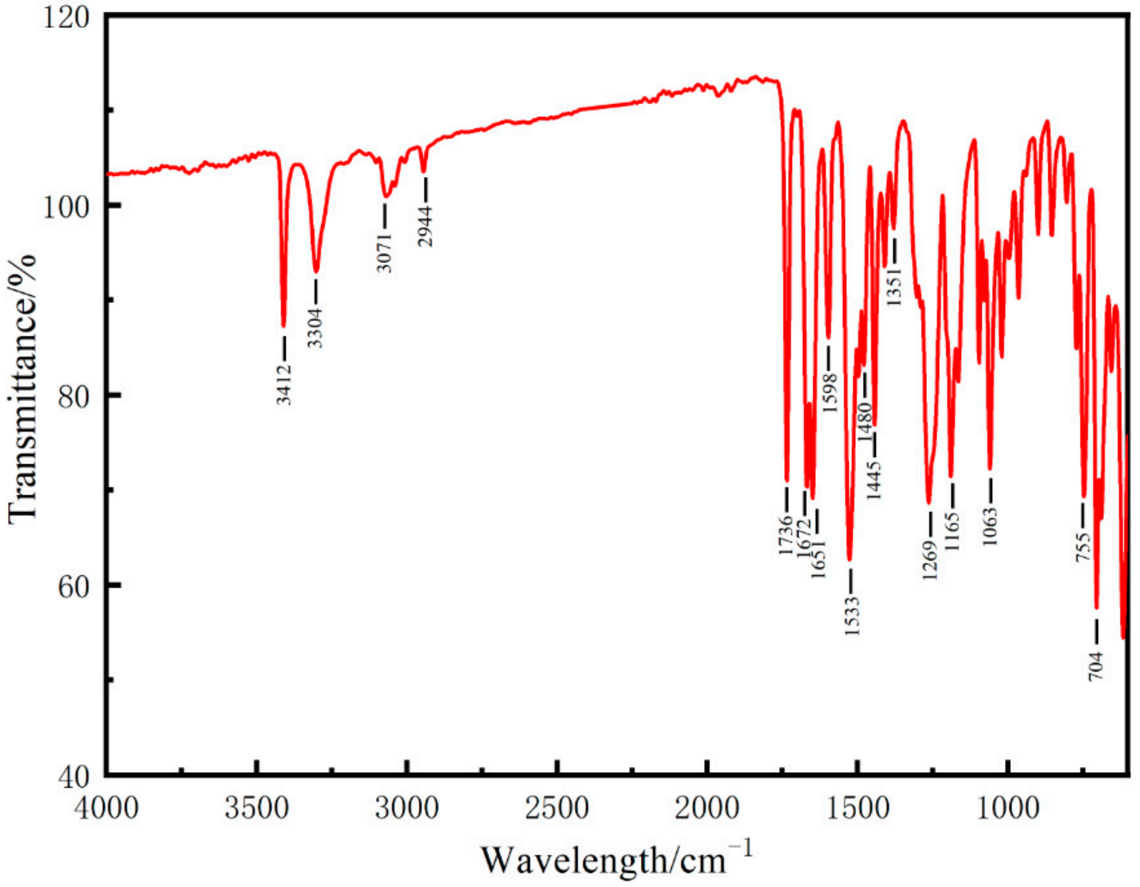

IR of compound **5c**

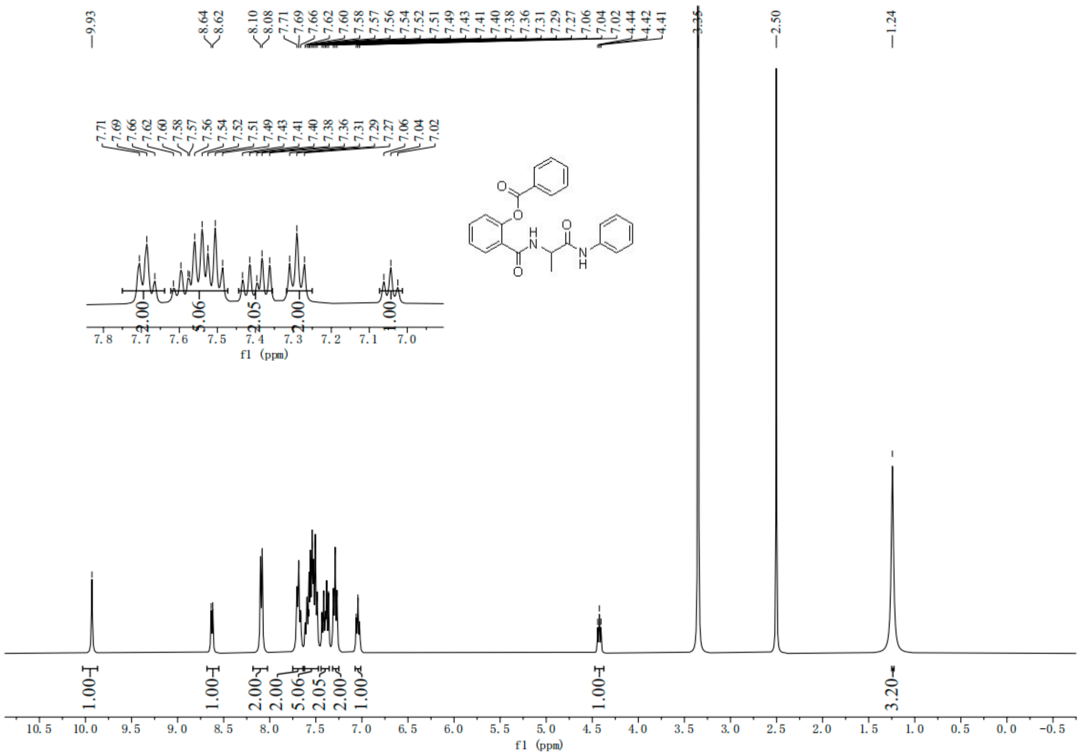

^{1}H NMR of compound **5d** (DMSO-d_6)

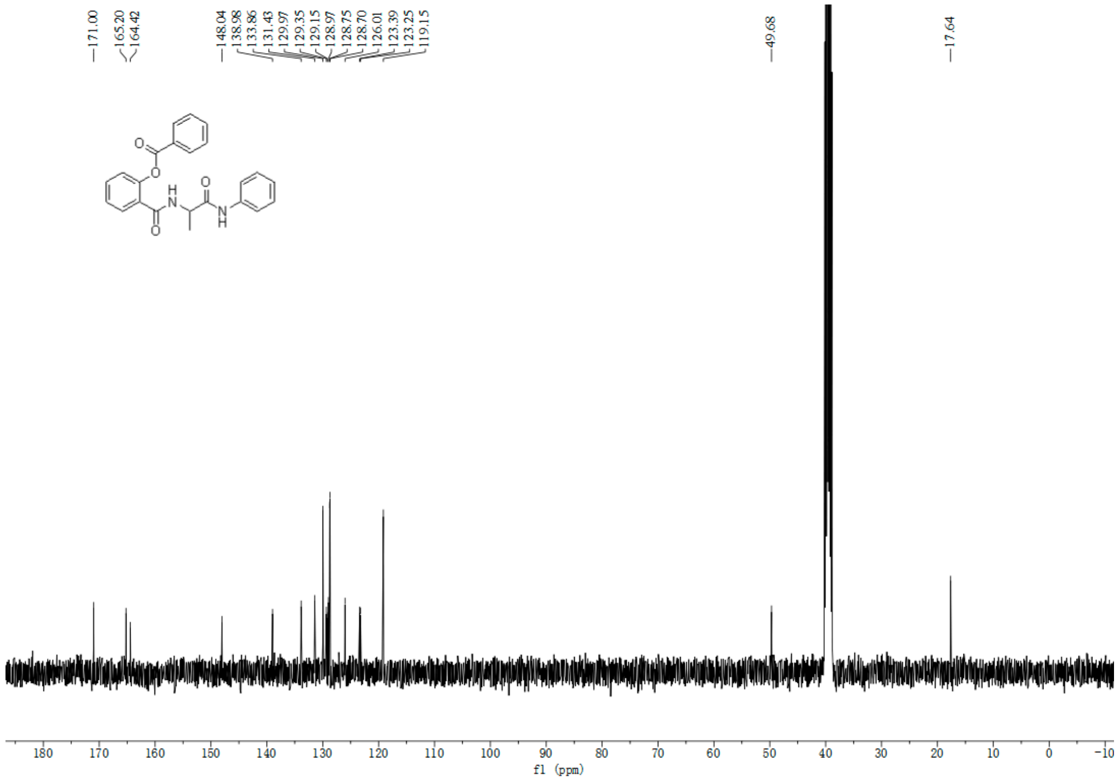

^{13}C NMR of compound **5d** (DMSO-d_6)

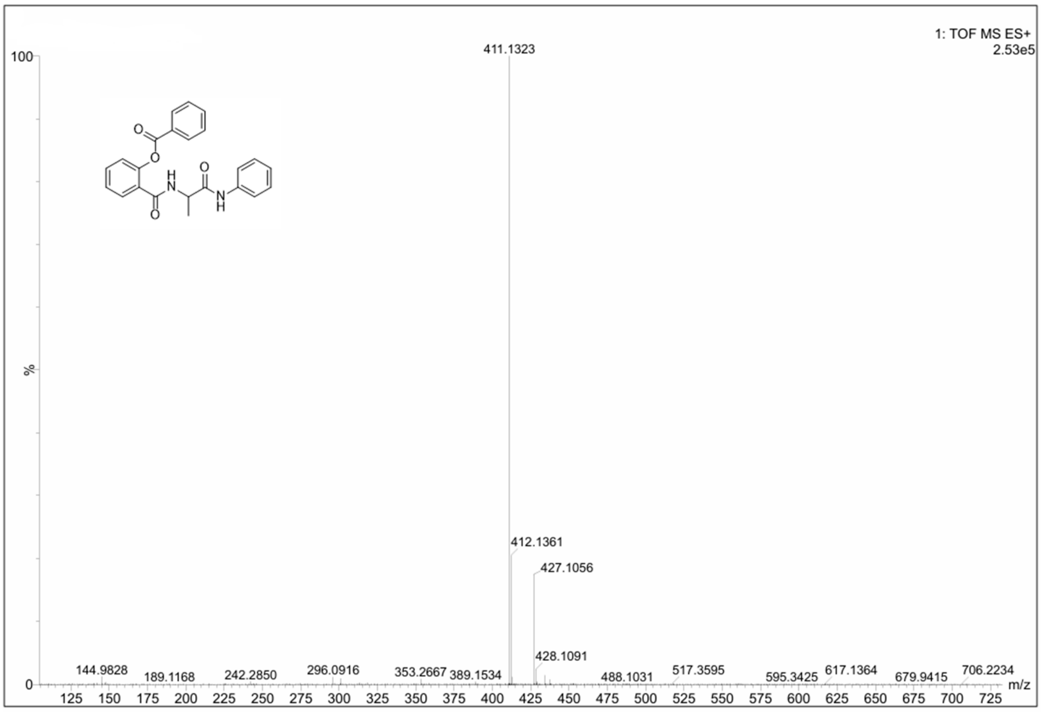

HRMS of compound **5d**

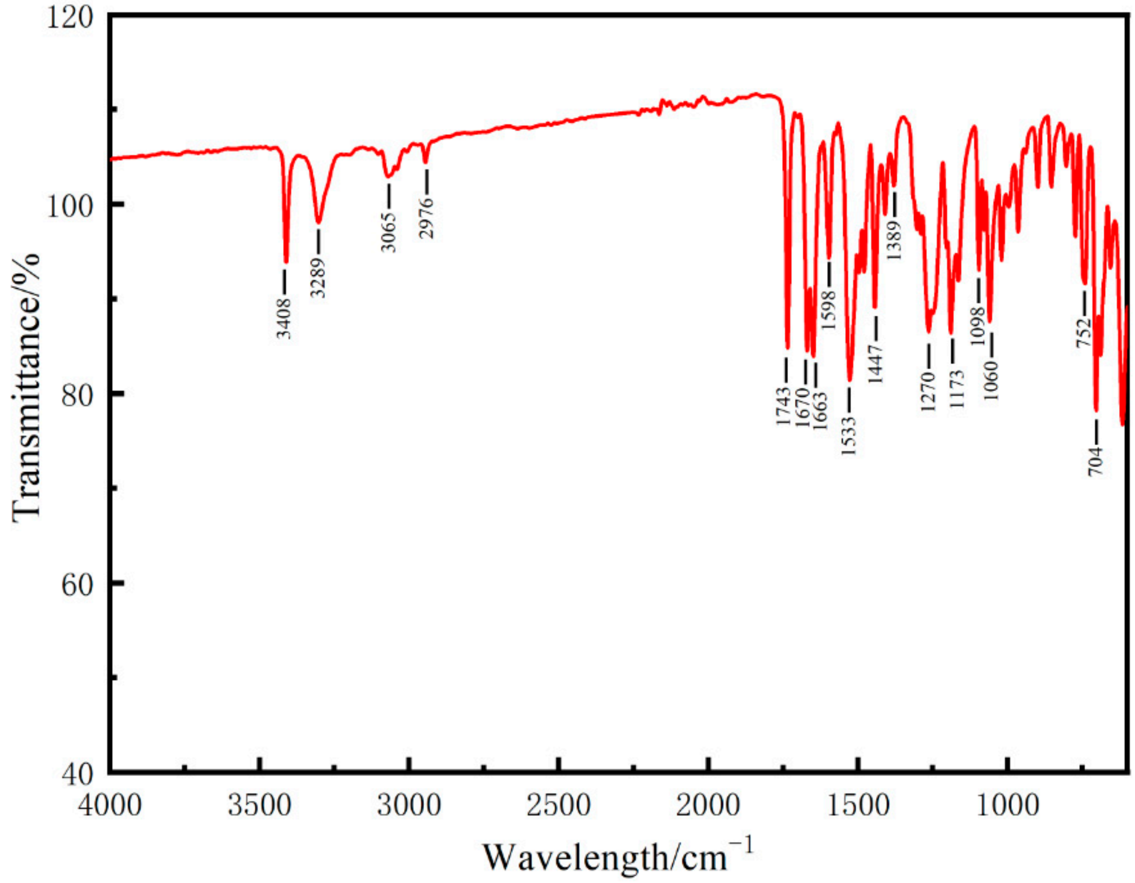

IR of compound **5d**

4. Molecular docking studies of compounds **3a** and **3h**

Compound **3a** exhibited π–π stacking interactions with Phe53 and Phe162, and formed direct hydrogen bonds with Asn131. Compound **3h** showed π–π stacking interactions with TYR50, Phe53, and Phe162.

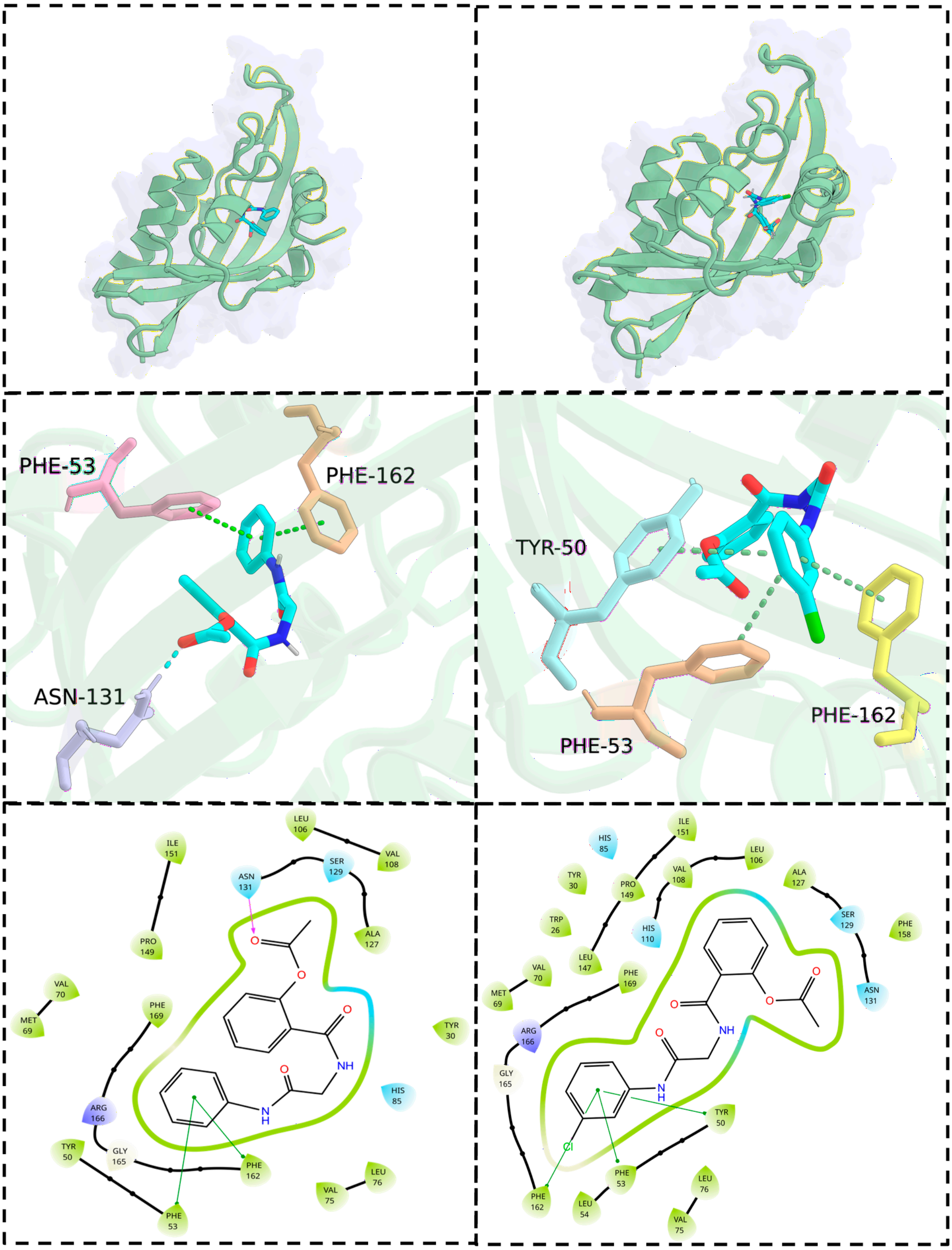

Figure S1. Molecular docking studies of compounds **3a** and **3h**.
